# Features of Variable Number of Tandem Repeats in *Yersinia pestis* and the Development of a Hierarchical Genotyping Scheme

**DOI:** 10.1371/journal.pone.0066567

**Published:** 2013-06-21

**Authors:** Yanjun Li, Yujun Cui, Baizhong Cui, Yanfeng Yan, Xianwei Yang, Haidong Wang, Zhizhen Qi, Qingwen Zhang, Xiao Xiao, Zhaobiao Guo, Cong Ma, Jing Wang, Yajun Song, Ruifu Yang

**Affiliations:** 1 State Key Laboratory of Pathogen and Biosecurity, Beijing Institute of Microbiology and Epidemiology, Beijing, China; 2 Laboratory Department, Navy General Hospital, Beijing, China; 3 Qinghai Institute for Endemic Diseases Prevention and Control, Xining, China; 4 Institute of Health Quarantine, Chinese Academy of Inspection and Quarantine, Beijing, China; Institut National de la Recherche Agronomique, France

## Abstract

**Background:**

Variable number of tandem repeats (VNTRs) that are widely distributed in the genome of *Yersinia pestis* proved to be useful markers for the genotyping and source-tracing of this notorious pathogen. In this study, we probed into the features of VNTRs in the *Y. pestis* genome and developed a simple hierarchical genotyping system based on optimized VNTR loci.

**Methodology/Principal Findings:**

Capillary electrophoresis was used in this study for multi-locus VNTR analysis (MLVA) in 956 *Y. pestis* strains. The general features and genetic diversities of 88 VNTR loci in *Y. pestis* were analyzed with BioNumerics, and a “14+12” loci-based hierarchical genotyping system, which is compatible with single nucleotide polymorphism-based phylogenic analysis, was established.

**Conclusions/Significance:**

Appropriate selection of target loci reduces the impact of homoplasies caused by the rapid mutation rates of VNTR loci. The optimized “14+12” loci are highly discriminative in genotyping and source-tracing *Y. pestis* for molecular epidemiological or microbial forensic investigations with less time and lower cost. An MLVA genotyping datasets of representative strains will improve future research on the source-tracing and microevolution of *Y. pestis.*

## Introduction

Plague, one of the most devastating infectious diseases in human history, is a reemerging infectious disease causing outbreaks in several areas since the early 1980s. *Yersinia pestis*, the causative agent of plague, has killed hundreds of millions of people in the three major historical plague pandemics [1], [2]. *Y. pestis* was listed as one of four Category A selected bacterial agents by the USA Center for Disease Control and Prevention, and it could be potentially used as a war weapon or bioterrorism agent in the future, posing significant threats on the health and safety of human beings [3]. The demand for preparedness for biological terrorism threats and natural plague outbreaks has renewed interest in the detection, identification, and source-tracing of *Y. pestis*, especially by methods that can determine the origin of the outbreak strain.

*Y. pestis* evolved from *Yersinia pseudotuberculosis* no later than 2,600 years ago [4], [5]. The relatively short evolutionary history of *Y. pestis* accounts for its limited phenotypic and genetic diversity [6], [7]. Traditionally, *Y. pestis* was classified into three biovars by western scientists [8], according to their ability to reduce nitrate and utilize glycerol: Antiqua (positive for both), Medievalis (negative for nitrate reduction and positive for glycerol utilization), and Orientalis (positive for nitrate reduction and negative for glycerol utilization). Microtus, a new biovar, are usually rhamnose-positive and of low virulence or avirulent for guinea pigs and has been proposed based on whole genome sequencing and genetic analysis [9], [10]. The Mcirotus biovar also includes strains from the FSU, which were called Pestoides by Russian scientists [11]. However, this biovar-based system provide little information for tracing the origin of the organism, and some biovars had been proven genetically heterogeneous [12]. Several methods have been developed for *Y. pestis* genotyping, including ribotyping, multi-locus variable number of tandem repeats (VNTRs) analysis (MLVA), clustered regularly interspaced short palindromic repeats (CRISPRs), different regions (DFRs), insertion sequence (IS) and single nucleotide polymorphisms (SNPs) [12]–[20].

SNPs can be used as gold markers for genotyping certain bacteria [21]. Utilizing next-generation sequencing technology and phylogenomic analysis, we portrayed the SNP profiles from 133 *Y. pestis* strains and constructed a full parsimony phylogenetic tree of this species based on 2,298 SNPs [22]. Morelli et al. defined 24 subpopulations in a global collection of 286 *Y. pestis* strains, by using 933 SNPs that identified from 17 whole genome sequences. [5]. Two sets of VNTR markers which include 25 and 46 loci respectively, were used by Pourcel et al. [4]. The whole genome-wide SNPs provide phylogenetic analysis of extremely high discrimination power, with almost every strain in one individual genotype. However, the time and cost of next-generation sequencing technology make it unfeasible for routine applications in most laboratories.

VNTR usually has higher mutation rate than SNP, and MLVA assay could provide higher discrimination power when multiple loci are used in genotyping, compared to SNP tests including only limited number of pre-identified SNPs [23]. Two sets of VNTR markers which include 25 and 46 loci respectively, were employed by Pourcel et al. [17] and Klevytska et al. [18], [24] to genotype *Y. pestis*. In addition to *Y. pestis*, other genetically monomorphic species, including *Bacillus anthracis*, *Acinetobacter baumannii, Mycobacterium tuberculosis*, and *Francisella tularensis*, were analyzed by MLVA to obtain fine-scaled genotyping results [25]–[28]. However, the high mutation rates of VNTR loci also increase the possibility of reversions and convergent mutations, which may lead to homoplasies and bias in phylogenetic reconstruction [21].

In this study, we investigated the polymorphisms and features of 88 VNTRs in *Y. pestis*, including 24 newly identified ones and 64 previously reported ones, in 97 strains with global diversity. We then developed a simple hierarchical MLVA genotyping scheme based on “14+12” selected loci from the 88 VNTRs, which is consistent with SNP analysis, with significant time- and cost-saving. A total of 956 strains were screened using this scheme to generate a genetic diversity datasets to investigate the source-tracing ability of this method for *Y. pestis.*

## Materials and Methods

### Strains and DNA

We selected 79 Chinese *Y. pestis* strains, 18 publicly available *Y. pestis* whole genome sequences, and 4 genomes of *Y. pseudotuberculosis* to evaluate the genome-wide polymorphisms of VNTR loci (Table S1). Additionally, 859 diverse *Y. pestis* strains, including 842 from 18 plague foci of China, isolated between1943 and 2005, and 17 from Mongolia, were selected and screened using the newly established MLVA genotyping method. Of the 956 *Y. pestis* strains, 909 were previously reported in DFR genotyping analysis [16]. All the Chinese isolates were collected by the Qinghai Institute for Endemic Diseases Prevention and Control, the Center for Disease Control and Prevention of Xinjiang Uygur Autonomous Region, and the Yunnan Institute for Endemic Disease Control and Prevention. The DNAs of the Mongolian isolates were kindly provided by Dr. Jing Wang from the Institute of Health Quarantine, Chinese Academy of Inspection and Quarantine. The bacteria were cultivated in nutrient agar at 26°C for 48 h, and the genomic DNAs were extracted by conventional sodium dodecyl sulfate lysis and phenol-chloroform extraction.

### VNTR Loci

Pourcel et al. [17] and Klevytska et al. [18], [24] independently developed two sets of MLVA typing protocols that contained 25 and 46 VNTRs loci, respectively. Given the fact that seven loci were shared by both protocols, 64 previously defined were re-evaluated in combination with the newly selected VNTR loci in this study. Tandem Repeats Finder 4.0 [29] was also used to find tandem repeats (TRs) in the chromosomes of five *Y. pestis* genomes (CO92, Nepal516, Antiqua, KIM, and 91001) [30]–[33], with alignment parameters of 2, 7, and 7 (match, mismatch, and indel), a minimum alignment score of 80, and a maximum period size of 200. A total of 280 VNTR loci were identified. Twenty-four of these loci that exhibited variations across the five genomes but were missed by previous studies were also selected in the present study for further analysis (Table 1). For consistency with previous nomenclatures, we did not change the names of the reported loci. Accordingly, loci named “M+number” were from Klevytska [18], [24], and those named “yp+number+ms+number” from Pourcel [17]. The newly identified loci in this research were named “N+number” (Table 1).

**Table 1:** Characteristics of the 88 VNTR loci.

VNTR ID	In ORF	ML$(bp)	CO92 Position	Alleles	DI	Reference
yp0120ms01	Overlap	18	120612.120755	5	0.41	[17]
yp0559ms15	No	15	559948.559977	2	0.12	[17]
yp0581ms40	No	17	581354.581472	4	0.54	[17]
yp0718ms41^#^	No	17	718786.718904	3	0.53	[17]
yp1018ms44	No	17	1018653.1018737	3	0.08	[17]
yp1118ms69	No	16	1118998.1119093	4	0.49	[17]
yp1335ms46	No	7	1335252.1335286	15	0.87	[17]
yp1580ms70	Yes	9	1580109.1580153	11	0.82	[17]
yp1895ms21	Yes	18	1895349.1895510	3	0.08	[17]
yp1925ms71	No	14	1925713.1925782	4	0.33	[17]
yp2058ms51	Yes	21	2058747.2058788	3	0.18	[17]
yp2769ms06	Overlap	60∼	2769354.2769833	16	0.85	[17]
yp3057ms09^#^	Yes	18	3058032.3058625	16	0.73	[17]
yp3060ms56^#^	Overlap	16	3060574.3060653	6	0.73	[17]
yp3236ms73^#^	Yes	18	3236823.3236912	4	0.52	[17]
yp3245ms74	Yes	15	3245601.3245690	4	0.52	[17]
yp4042ms35	Yes	15	4042417.4042491	4	0.56	[17]
yp4425ms38^#^	No	16	4425343.4425470	7	0.68	[17]
M02	No	1	3122635.3122644	5	0.36	[18]
M06	No	2	462711.462722	7	0.66	[18]
M09	Yes	3	2871853.2871870	7	0.22	[18]
M12c	No	4	2325280.2325319	14	0.88	[18]
M15^#^	No	5	3696792.3696816	7	0.61	[18]
M18	No	6	2771484.2771519	12	0.86	[18]
M21^#^	No	7	427497.427531	7	0.74	[18]
M22^*^	No	7	1343780.1343870	23	0.93	[18]
M23^*^	No	7	3458682.3458730	16	0.89	[18]
M25^*^	No	7	2984639.2984792	21	0.93	[18]
M26	No	8	3358733.3358748	2	0.18	[18]
M27	No	8	3081535.3081686	26	0.94	[18]
M28^*^	Overlap	8	1553973.1554028	13	0.89	[18]
M29^*^	No	8	2238495.2238558	11	0.8	[18]
M31	No	8	2315966.2316029	12	0.88	[18]
M33^*^	Yes	9	845399.845632	15	0.85	[18]
M36	No	10	2878786.2878805	7	0.54	[18]
M41	Yes	12	1896606.1896629	2	0.02	[18]
M43^*^	No	12	174513.174572	9	0.78	[18]
M49	No	14	3158808.3158849	4	0.39	[18]
M52	Yes	15	1198069.1198113	2	0.18	[18]
M54	No	16	932708.932755	8	0.82	[18]
M55	No	16	1868893.1868924	4	0.27	[18]
M56	Overlap	17	2640246.2640279	5	0.26	[18]
M61^#^	No	18	2552583.2552672	7	0.6	[18]
M65	Overlap	19	2999986.3000042	4	0.14	[18]
M66	No	20	3555712.3555791	4	0.36	[18]
M68	No	20	150777.150816	7	0.27	[18]
M69	Yes	21	772786.772827	1	0	[18]
M71	Overlap	21	3768639.3768680	4	0.12	[18]
M73	Yes	30	2748784.2748843	3	0.13	[18]
M74	Yes	36	3114610.3114681	3	0.17	[18]
M76	Overlap	41	4366037.4366118	2	0.27	[18]
M77	Overlap	45	54675.54899	4	0.23	[18]
M79	No	8	3832848.3832927	14	0.88	[18]
M37(ms07)	No	10	2916163.2916232	7	0.56	[17] & [18]
M72(ms54)	No	22	2612799.2612842	3	0.51	[17] & [18]
M59(ms05)	Overlap	17	1935787.1935939	7	0.47	[17] & [18]
M58(ms04) ^#^	No	17	1290131.1290266	8	0.71	[17] & [18]
M51(ms20)	Yes	15	1814480.1814524	4	0.51	[17] & [18]
M42(ms45)	No	12	1108967.1109002	4	0.18	[17] & [18]
M34(ms62)^*^	Yes	9	4280839.4280892	22	0.93	[17] & [18]
M19	No	6	809405.809578	28	0.89	[24]
M24	No	7	808089.808130	6	0.69	[24]
M70	Yes	21	3793499.3793540	2	0.02	[24]
M75	Yes	18	802532.802675	7	0.62	[24]
N0171	Overlap	18	172086.172157	5	0.38	This study
N0320	Overlap	8	3208218.3208273	12	0.81	This study
N0322	No	7	322213.322254	12	0.86	This study
N0335	No	18	335291.335362	6	0.39	This study
N0865^#^	No	18	865723.865758	5	0.63	This study
N1093	Yes	18	1093917.1093970	3	0.1	This study
N1398	Overlap	17	1398764.1398814	4	0.25	This study
N1606^*^	No	9	1607058.1607093	7	0.81	This study
N1736	Yes	27	1736544.1736570	2	0.39	This study
N2117^*^	Yes	9	2117885.2117911	12	0.86	This study
N2486^#^	No	17	2486193.2486243	3	0.56	This study
N2577^*^	No	7	2577991.2578039	14	0.85	This study
N2896^#^	No	16	2896594.2896641	6	0.73	This study
N2976^#^	No	18	2976893.2976946	4	0.55	This study
N3322	No	19	3322024.3322099	4	0.42	This study
N3624	Yes	18	3624857.3624928	2	0.06	This study
N3634	Overlap	16	3634057.3634120	3	0.15	This study
N3743	No	8	3743960.3743999	12	0.85	This study
N3763	Yes	18	3763926.3763997	4	0.33	This study
N3773^*^	Yes	9	3773759.3773803	10	0.85	This study
N3779^#^	No	17	3780032.3780099	4	0.55	This study
N4268	No	7	4268345.4268414	25	0.95	This study
N4287	Overlap	19	4287530.4287567	5	0.37	This study
N4556	Overlap	19	4556736.4556792	4	0.17	This study

14+12 VNTRs used in rapid genotyping system were labeled with ^#^ and ^*^, separately.

$ ML: motif length, DI: Nei’s diversity index.

The primers used in this study, including 64 previously reported ones [17], [18], [24] and 24 for amplifying newly identified VNTRs, are listed in Table S2. All primer sets were labeled with a phosphoramidite fluorescent dye (6-Fam and Hex) on the 5′ terminus.

### PCR Amplification

A solution of 10 µL of PCR mixture contained 4 ng of DNA template, 0.5 µM of each primer, 0.4 unit of *Taq* DNA polymerase, 70 µM of dNTPs, and 10×PCR buffer (170 mM KCl, 35 mM Tris-HCl, 8 mM MgCl_2_, pH 8.3).The amplification was carried out in a DNA thermocycler (MJ Research PTC-225) with pre-denaturation at 95°C for 5 min, followed by 30 cycles of denaturation at 95°C for 40 s, annealing at 58°C for 40 s, and elongation at 72°C for 1 min. A final 5-min elongation at 72°C was performed after the last cycle to ensure complete amplicon extension. The DNA of strain 91001 was amplified together as reference in each run.

### GeneScan Fragment Analysis

The PCR products were diluted five times and mixed with formamide and Rox 500-labeled fragment standards at a ratio of 2∶7:1. Fluorescently labeled amplicons were visualized by capillary electrophoresis on ABI PRISM 310 Genetic Analyzer. Amplicon sizes were estimated with Applied Biosystems GeneScan analysis software, and the sizes of amplicons from strain 91001 were used as references to determine the copy number for each allele as previously described [17].

### Clustering Analysis

The copy numbers of all VNTRs were imported into the BioNumerics software package version 5.10 (Applied-Maths, Belgium) as character datasets. Clustering analysis of VNTR results was performed using the categorical multi-state coefficient and the Ward dendrogram. The minimal spanning tree (MSTree) was constructed from a similarity matrix.

## Results and Discussion

### General Features of VNTR Loci

**Figure 1:**
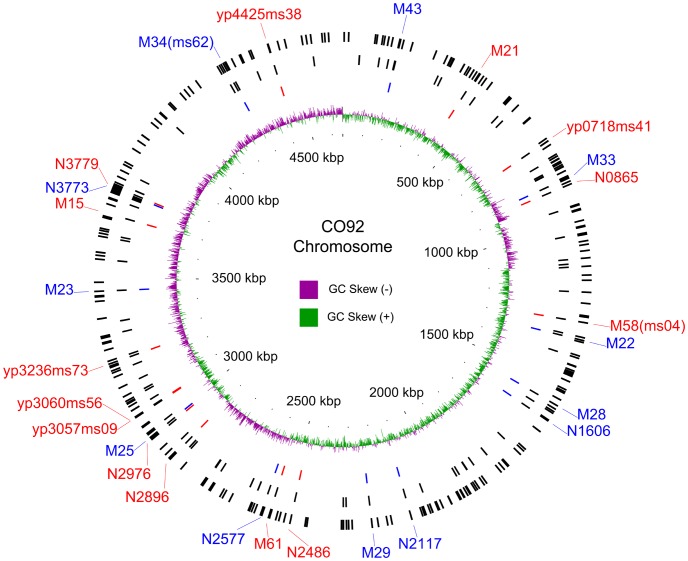
Distribution of the tandem repeats in CO92 genome. From outer to inner, the bars in circles displayed i) the genomic positions of 280 tandem repeats, ii) position of 88 VNTRs, and iii) position of 14+12 VNTRs used in the hierarchical genotyping system. Red font indicates the 14 VNTRs used in the first step typing, and blue indicates the 12 VNTRs in the second step.

TR regions are widely distributed in the *Y. pestis* genome, with 280 alleles of the TRs identified from five published genomes (Figure 1). In addition to the 64 previously defined loci, 24 new ones were selected because of their differences of repeat numbers across the five genomes. The 88 loci mentioned above were further analyzed in this study (Table 1). The motif lengths of these repeats varied from 1 bp to 60 bp, 84.1% ranging from 7 bp to 21 bp. More than half (48/88, 54.5%) of the VNTRs were located in the intergenic regions. Twenty-four (27.3%) VNTRs were located within the ORFs, and their repeat unit lengths were divisible by three, indicating in-frame insertion/deletions in the corresponding proteins (Table 1).

Sixteen VNTRs overlapped with the ORFs, with the start or stop codon of the corresponding ORF located within the repeat regions. Six of these VNTRs overlapped with the start codons (Table 2), the copy number variations of which can cause elongation/shortening or frame shifts and possibly lead to inactivation of the ORFs (Figure S1A). Ten VNTRs overlapped with the stop codons (Table 2). In contrast to that of the VNTRs with start codons, the copy number variation in these VNTRs might have less effect on the encoding of ORFs (Figure S1B).

**Table 2:** Repeat sequences and features of 16 VNTRs that overlapped with ORFs.

VNTR ID	Motif sequence of VNTRs (5′-3′)*	Position of overlapping	Gene ID	Production
N1398	AGTACGGAG**GTG**ATAAA	5′-end	YPO1239	putative bacteriophage protein
M77	CAACGTTGGCA**TTG**GATATCCAGGTGGGACCGTGGTCCCAATTAC	5′-end	pCD76	hypothetical protein
M71	TTTGCTCTA**TTG**ATCTTGCGC	5′-end	YPO3379	hypothetical protein
N3634	GTTAT**TTG**ATAGGACC	5′-end	YPO3262	hypothetical protein
M56	TATCCCT**ATG**ACGCTAT	5′-end	YPO2346	conserved hypothetical protein
yp2769ms06	TTTCTAAGCTGCCTGTGCAGCAGTGAAC+ spacer**	5′-end	YPO2469	hypothetical protein
yp3060ms56	AGATTGTATT**TGA**AAT	3′-end	YPO2727	conserved hypothetical protein
yp0120ms01	GCAGT**TAA**CCTTTTCACT	3′-end	YPO0112	conserved hypothetical protein
N0320	AG**TGA**CGC	3′-end	YPO2872	exodeoxyribonuclease VII large subunit
N4287	CGAGATGTT**TGA**TTAGGTC	3′-end	YPO3820	putative P-type cation-translocating membrane ATPase
N4556	GAG**TAG**TTGTCAAGAGTGC	3′-end	YPO4039	xylulose kinase
M28	GT**TAA**TTG	3′-end	YPO1379	seryl-tRNA synthetase
N0171	GT**TAA**GGTTAGCTATGCA	3′-end	YPO0158	siroheme synthase
M76	TGTGGTGATCTAAGCGCAACACAGTTAAAAACAGATTT**TAA**	3′-end	YPO3891	hypothetical protein
M65	TAACAAGTTCAATATG**TAA**	3′-end	YPO2674	putative exported protein
M59(ms05)	TTAGCCCATATCAG**TAA**	3′-end	YPO1697	putative chaperone protein; K07346 fimbrial chaperone protein

* Bold font indicates start and stop codons.

** yp2769ms06 was a CRISPR locus, the motif sequence of which was composed by “direct sequence+spacer.” The start codon of YPO2469 was located within the first spacer of this CRISPR.

### Diversity of VNTRs in*Y. pestis* Populations

According to the whole genome-wide SNPs analysis, the population structure of *Y. pestis* was described as including 5 main branches (Branch 0–4), 9 major populations and 23 sub-populations [4], [5]. We selected 97 strains that belonged to 21 sub-populations representing most of the genetic diversity of *Y. pestis* (Figure S2 and Table S1), the DNA from another two sub-populations, 3.ANT2 and 4.ANT1, were unavailable in this study for determining polymorphism of 88 VNTRs.

**Figure 2:**
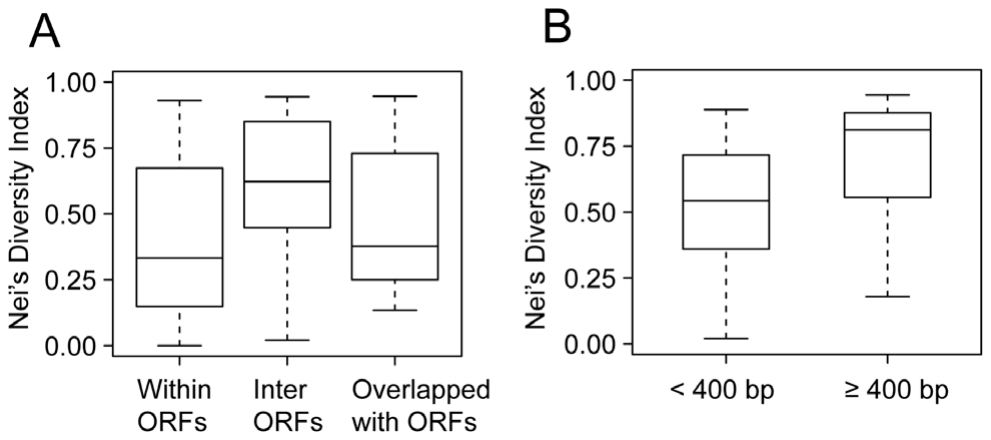
Nei’s DI across VNTRs in different genomic positions. A. DIs across three groups of 88 VNTRs according to their relative position to ORFs. B. DIs of 48 VNTRs located between ORFs. The two groups were defined by their distance to the 5′-end of the downstream ORF. Box plot indicates median (horizontal line), interquartile range (box), and minimum and maximum values (whiskers).

It was presumed that although VNTRs had high mutation rates [23], purifying selection would have purged the deleterious mutations, which resulted in VNTRs of different diversity according to their functional importance. To quantify the genetic diversity of VNTRs, we calculated the Nei’s diversity index [DI = 1−∑(allele frequency)^2^] of 97 strains for each VNTR locus. The VNTRs in *Y. pestis* exhibited high variability, with the DIs ranging from 0 to 0.95. M69, identified by Klevytska et al., revealed no diversity in our samples, with a DI of zero (Table 1). As expected, the VNTRs in the intergenic regions showed a significantly higher DI than those within ORFs (Figure 2A, Mann–Whitney *U* test, P = 0.0018). Interestingly, for the 48 VNTRs located between ORFs, the DIs were largely different according to their relative positions to the neighboring genes. The VNTRs located less than 400 bp from the 5′ end of the genes, the copy number variations of which were supposed to affect the binding of transcription factors to the promoter region, showed a lower DI (Figure 2B, Mann–Whitney *U* test, P = 0.0286). Thus, the polymorphisms of VNTRs in such genomic regions possibly influenced gene transcription regulation, thereby experiencing stronger purifying selection.

As mentioned above, six VNTRs overlapped with the 5′ end of the ORFs, and their variations were supposed to result in length variation or frame shift mutations of the corresponding genes (Table 2). Therefore, except for that of yp2769ms06, a CRISPR locus rather than a real VNTR, the DIs of the five other VNTRs were also constrained by purifying selection, presenting low values of 0.12 to 0.26 (Table 1).

The 10 VNTRs that overlapped with the 3′ end of the ORFs were supposed to have relatively higher DIs because they received less selection pressure. However, the loci among them still exhibited very low DIs. For example, the DI of locus M65, overlapping with an ORF that encoded putative exported protein, was 0.14. Another locus, N4556, which overlapped with a xylulose kinase encoding gene, revealed a DI value of 0.17. The low DIs suggest that such VNTR variations might also affect gene function and was therefore constrained by selection pressures. One possible explanation was that the motifs in these loci were not 100% identical, and the copy number variations would lead to frame shift mutations. Although the DIs implied a possible relationship between the polymorphism of VNTRs and the function of the associated genes, further investigation needs to be performed to provide more robust evidences on such correlations.

### Loci Selection for Hierarchical Genotyping

**Figure 3:**
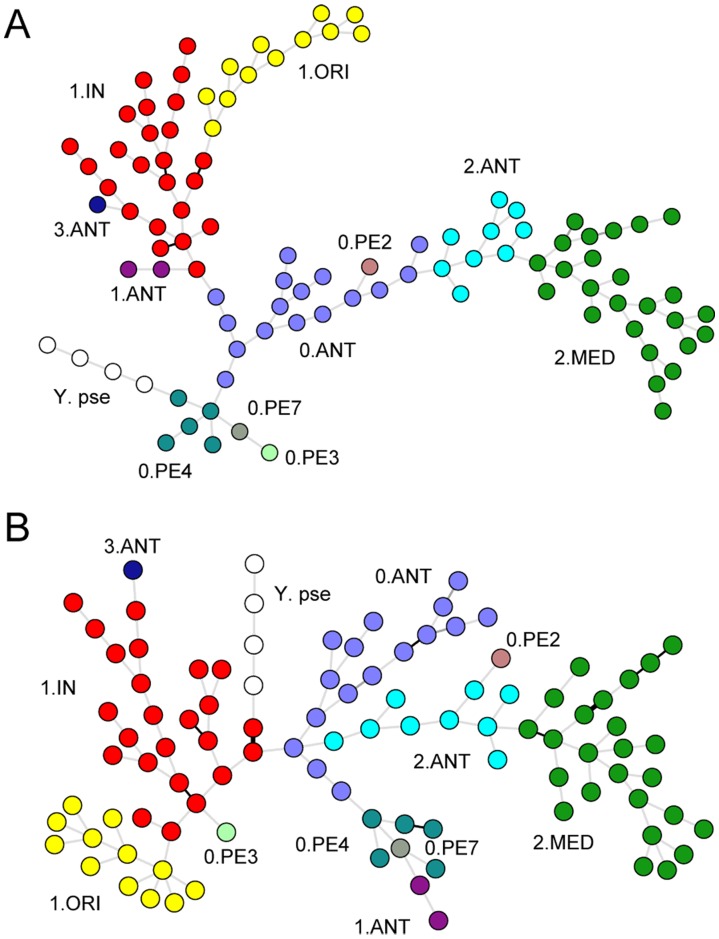
Clustering analysis of 97*Y. pestis* and four *Y. pseudotuberculosis* strains based on different sets of VNTRs. **A.** MSTree based on 88 loci, **B.** MSTree based on 39 loci. SNP-based populations were labeled with different colors.

Using all 88 VNTRs in genotyping *Y. pestis* could generate extremely high resolution, which distinguished all 97 strains into unique genotypes for each (Figure 3A). However, using too many loci would largely increase operation time and workload, which will not satisfy the need for rapid genotyping and source-tracing when facing plague natural outbreak or bioterrorism attack. Therefore, we optimized the combination of VNTRs (Figure S3), which used a small portion of loci to reduce the time and costs, without significantly decreasing the discrimination power or distorting the accuracy of the genotyping results (Figure S2).

Firstly, we removed the VNTRs in the known accessory genome to avoid the bias caused by possible missing data in genotyping work. Twenty-three large genomic regions (termed as Different regions, DFRs) were previously reported missing in certain *Y. pestis* strains (i.e., located in accessory genome) [16], [34], [35], and six of the 88 VNTRs were located or overlapped with these DFRs. The absence of genomic regions led to failed amplifications of alleles in some strains, such as M75. Seventeen strains (17.5%) obtained no amplicon for this locus, which resulted in a large amount of missing data. Therefore, these six VNTRs were excluded from the genotyping scheme.

Secondly, we removed yp2769ms06, which was actually a CRISPR rather than a VNTR locus. Although CRISPR is an alternative genotyping marker for bacteria [13], [36], [37], we still excluded it from the MLVA typing system for two reasons: a). The CRISPR had different variation patterns and more complicated mutation mechanisms compared with normal VNTRs. Therefore, the cluster analysis that combined the variations generated from the two types of repeat regions could lead to bias; b). Yp2769ms06, defined as “YPa” in previous researches [13], [14], has approximately 14 repeat units, corresponding to *ca*. 1 kbp of amplicons for this locus. According to our observation, the large size and specific arrangement of the nucleotide sequence of the YPa would cause difficult in amplification and copy number determination.

Thirdly, as the loci with low DI values contributed relatively less to the discrimination power of the whole scheme, we removed 42 VNTRs with DI less than 0.5. Based on the remaining 39 VNTRs, we built an MSTree for the 97 *Y. pestis* strains using four *Y. pseudotuberculosis* strains as the outgroup (Figure 3B). Although 49 VNTRs were removed, the remaining ones provided similar discrimination and could still distinguish all strains into unique genotypes for each. However, the phylogenetic relationship across the strains revealed by 39-loci MLVA deviated slightly from the population structure defined by SNP analysis (Figure S2) [4], [5]. For example, 0.PE2 in Branch 0 was clustered with 2.ANT in Branch 2, and the 1.ANT of Branch 1 was grouped with strains of Branch 0. The high mutation rate of VNTRs increases the rate of homoplasies in phylogenetic reconstruction and confounds the phylogenetic relationship across the strains [6]. Considering that all 39 VNTRs, which were supposed to possess higher mutation rates and homoplasy rates [23], revealed higher diversities, the confound phylogenetic relationships across these strains based on those loci are understandable.

**Figure 4:**
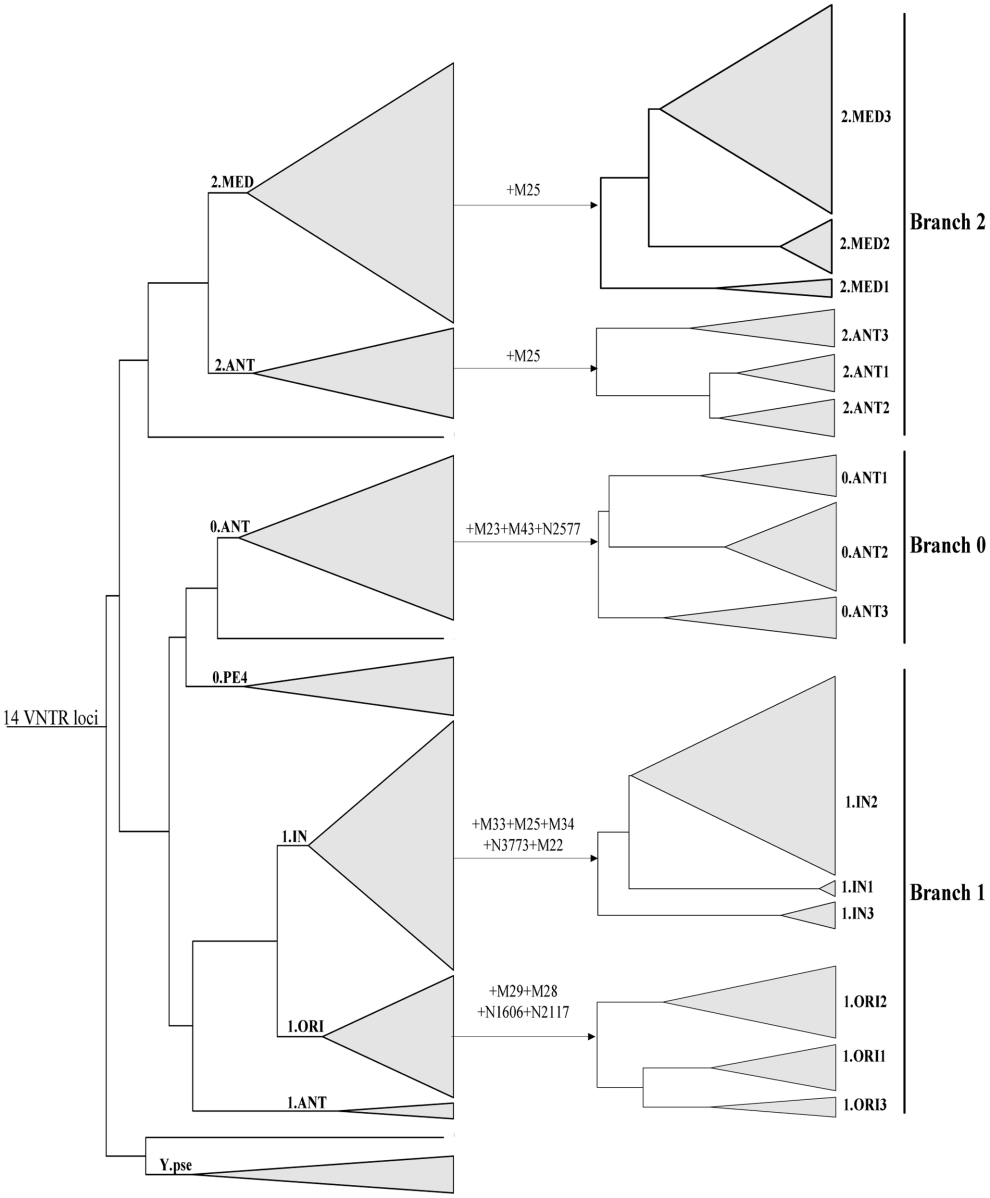
Genotyping results of 97*Y. pestis* and four *Y. pseudotuberculosis* strains based on 14+12 VNTRs. The trees were built by Ward. The twelve VNTRs used for defining the sub-populations were indicated on the arrows.

Fourthly, for further reducing the number of loci and acquiring a genotyping result as consistent as possible with phylogeny based on SNPs, we investigated the phylogenies constructed by all of the possible combination of the subsets from these 39 VNTRs. Unfortunately, no VNTR combination could perfectly reconstruct the population structure defined by SNPs. A 14-VNTR scheme produced a phylogeny that mostly approximated the SNP-based analysis, in which all strains were clustered into the major populations correctly (Figure 4). However, the discrimination power of 14 VNTRs was not high enough to resolve the strains into subpopulation level. Therefore, in addition to these 14 VNTR loci, we used another 12 VNTRs from the previous 39 ones to further distinguish the strains of major populations into different sub-populations. As shown in Figure 4, with the loci M23, M43, and N2577, the major population 0.ANT could be divided into sub-population 0.ANT1, 0.ANT2, and 0.ANT3. Five additional loci M33, M25, M34, N3773, and M22 could resolve the 1.IN into 1.IN1, 2, and 3. 1.ORI could be divided into sub-population 1.ORI1, 2, and 3 by adding four loci M29, M28, N1606, and N2117. M25 could classify 2.MED into 2.MED1, 2, and 3 and 2.ANT into 2.ANT1, 2, and 3.

Finally, a hierarchical genotyping scheme was established using “14+12” VNTR loci, which could define the *Y. pestis* strains into sub-populations. Briefly, a 14-VNTR locus analysis designates isolates into major populations, and then additional one to five loci could classify them into sub-populations. Notably, if only a limited number of strains need to be genotyped, it would be more convenient to determine the polymorphism of all 26 VNTRs simultaneously before performing a hierarchical analysis.

### Establishment of Extensive MLVA Datasets for Source-tracing Plague Outbreaks

The accuracy of source-tracing would depend not only on the robust typing method but also on extensive datasets including samples with as much diversity as possible. Accordingly, we determined the profiles of “14+12” VNTR loci in additional 859 *Y. pestis* strains. Combining these with the MLVA results of 97 strains used for scheme development (Table S1), the datasets including genotyping information on 956 *Y. pestis* strains was obtained.

As most of these strains had detailed information on isolation time and location, these data consisted of valuable resources for tracing the source of plague outbreaks. Based on the primary 14 VNTRs, we reconstructed the phylogeny of these strains. The geographical clustering patterns of the MLVA genotypes, with the strains isolated from the same or neighboring region, were grouped together in a manner consistent with the SNP analysis (Figure S4) [4]. Then, with the secondary 12 loci, we separately constructed phylogenetic trees of the strains of five major populations to validate the discrimination power of these loci. A total of 139 strains, including 25 pre-defined 2.MED ones, were clustered based on 14 loci and M25 (Figure S5). All strains were group into three major branches with 2.MED3, 2.MED2, and 2.MED1. A total of 137 strains, including nine pre-defined 2.ANT ones, were clustered based on the same set of VNTRs used in 2.MED (Figure S6). Except for one 2.ANT2 strain 351001 grouped with three 2.ANT1 ones, the other two sub-populations in 2.ANT were clustered with their relatives. Sixteen pre-defined 0.ANT1, 2, and 3 strains clustered with their close-related ones, respectively, with 14 primary VNTRs and M23, M43, as well as N2577 (Figure S7). For population 1.IN, 207 isolates were divided into three clusters, 1.IN1, 2 and 3, with their respective closely related ones together based on 14 loci and M34, M33, M25, M22, as well as N3773 (Figure S8).

Biovar Orientalis is a relatively young group, and fewer variations were accumulated in their genomes. Thus, strains of the 1.ORI population did not perfectly cluster into 1.ORI1, 2, and 3 sub-populations (Figure S9). For example, strain IP 275 (pre-defined as 1.ORI3) grouped with a strain of 1.ORI2, and another strain of 1.ORI3, MG05-1020, grouped with four 1.ORI1 strains. As the copy number data of IP275 and MG05-1020 were deciphered from their draft genome sequences, the distorted clustering results were possibly caused by sequencing or assembly errors.

Although certain strains from several sub-populations were not clustered perfectly by the hierarchical scheme of “14+12” VNTR loci, this scheme would provide a high-resolution population structure of *Y. pestis*, which is compatible with SNP-based phylogeny.

### Conclusions

In this study, we investigated the features of 88 diversified VNTRs in *Y. pestis* and developed a simple genotyping scheme based on the selected “14+12” VNTRs. This hierarchical genotyping method not only provides enough discrimination power but also reduces time and cost substantially. This scheme also reduces the possible distortion of homoplasies caused by rapid mutations of VNTR loci. Meanwhile, an MLVA genotyping datasets including 956 strains was obtained for future source-tracing attempts. We noticed that Microtus strains showed heterogeneous typing results by this scheme. Other MLVA assays also failed to reconstruct reliable phylogenetic topology for the ancient Microtus (Pestoides) strains [19], [38]. This is might because of the fact that, some VNTR loci mutated so fast that they got saturated during the evolution and led to homoplasies in different lineages [23]. An alternative typing scheme will be a hierarchical assay consisting of canonical SNP analysis to determine the major phylogenetic groups, and subsequent specific MLVA to determine detailed position in the terminals of the phylogenetic tree, as what had been achieved in *Bacillus anthracis* [39].

The large amount of genetic diversity information provided by this study could also benefit the microevolution research on *Y. pestis*. However, most strains in this datasets were from China, and MLVA information on more *Y. pestis* strains from other countries by international collaborations is necessary to enrich and validate this datasets for global microbial forensics investigation of this pathogen [29].

## Supporting Information

Figure S1 **The possible influence on gene coding by variations of VNTRs that overlapped with ORFs.** The ORF was indicated by grey, and motifs of VNTR were colored by blue. A. The motifs that contained start codon. The insertion or deletion of motif would possibly result in length variation of the ORF. B. The motifs that contained end codon. The variation of copy number wouldn’t change the coding of ORF.
(PDF)

Figure S2 **SNP-defined phylogenetic relationship of 97** ***Y. pestis*** **strains.** The tree was plotted according to previously reported phylogenies [4], [5]. Each circle represents one sub-population, and the size indicates the number of strains. The major populations are indicated by colored circles. The strains from 3.ANT2 and 4.ANT1 were unavailable, and the branches leading to these two populations are indicated by dot lines.
(PDF)

Figure S3 **Procedure of locus selection for rapid genotyping system.**
(PDF)

Figure S4 **Dendrogram of 956 strains based on 14 primary VNTR loci.**
(PDF)

Figure S5 **Dendrogram of** ***Y. pestis*** **strains clustered with 2.MED population based on 14+1 VNTR loci.** A total of 139 strains were analyzed according to the profiles of 14 primary VNTRs and of the locus M25.
(PDF)

Figure S6 **Dendrogram of** ***Y. pestis*** **strains clustered with 2.ANT population based on 14+1 VNTR loci.** A total of 137 strains were analyzed according to the profiles of 14 primary VNTRs and the locus M25.
(PDF)

Figure S7 **Dendrogram of** ***Y. pestis*** **strains clustered with 0.ANT population based on 14+3 VNTR loci.** A total of 186 strains were analyzed according to the profiles of 14 primary VNTRs and the loci M23, N2577, and M43
(PDF)

Figure S8 **Dendrogram of** ***Y. pestis*** **strains clustered with 1.IN population based on 14+5 VNTR loci.** A total of 207 strains were analyzed according to the profiles of 14 primary VNTRs and the loci M34, N3773, M33, M25, and M22.
(PDF)

Figure S9 **Dendrogram of** ***Y. pestis*** **strains clustered with 1.ORI population based on 14+4 VNTR loci.** A total of 196 strains were analyzed according to the profiles of 14 primary VNTRs and the loci M29, M28, N1606, and N2117.
(PDF)

Table S1 **Information and repeat numbers in 14+12 loci of 97 representative** ***Y. pestis*** **and 4** ***Y. pseudotuberculosis*** **strains**
(XLSX)

Table S2 **Primers of 88 VNTR loci used in this study.** The locus name was described as “locus name_unit length_amplicon size_repeat numbers” in CO92 genome
(XLSX)
