# Supplementary figures and images for: Features of Variable Number of Tandem Repeats in Yersinia pestis and the Development of a Hierarchical Genotyping Scheme
Source: PLoS One. 2013 Jun 21;8(6):e66567. doi: 10.1371/journal.pone.0066567 (PMC3689786; doi:10.1371/journal.pone.0066567)

A

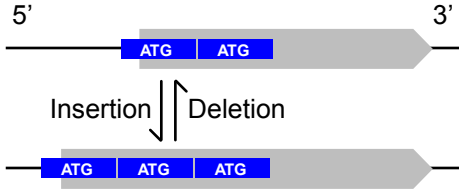

B

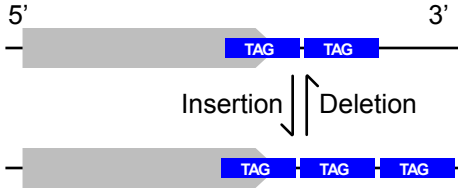

Supplement: Figure S1 — The possible influence on gene coding by variations of VNTRs that overlapped with ORFs. The ORF was indicated by grey, and motifs of VNTR were colored by blue. A. The motifs that contained start codon. The insertion or deletion of motif would possibly result in length variation of the ORF. B. The motifs that contained end codon. The variation of copy number wouldn’t change the coding of ORF. (PDF) [file pone.0066567.s001.pdf]

Branch 1

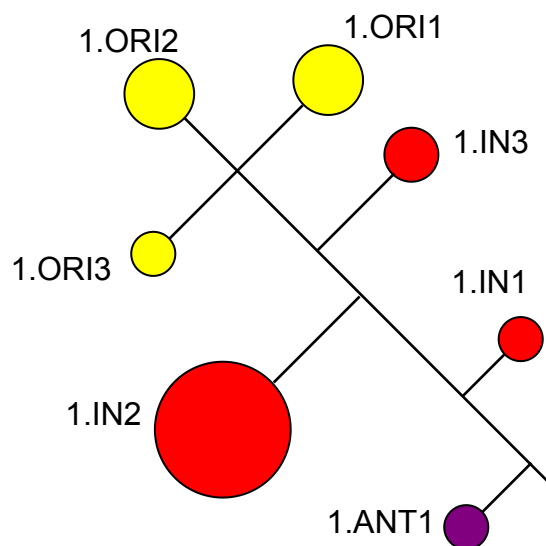

Branch 2

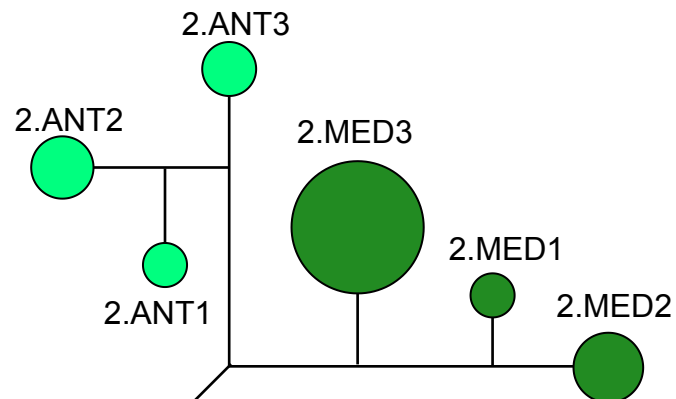

Branch 3

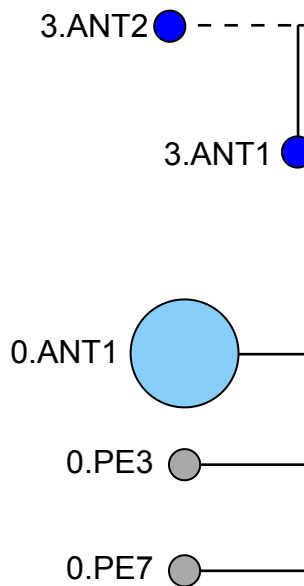

Branch 4

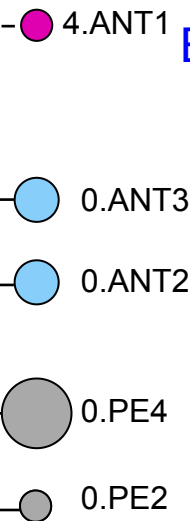

Branch 0

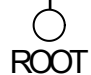

Supplement: Figure S2 — SNP-defined phylogenetic relationship of 97 Y. pestis strains. The tree was plotted according to previously reported phylogenies [4], [5]. Each circle represents one sub-population, and the size indicates the number of strains. The major populations are indicated by colored circles. The strains from 3.ANT2 and 4.ANT1 were unavailable, and the branches leading to these two populations are indicated by dot lines. (PDF) [file pone.0066567.s002.pdf]

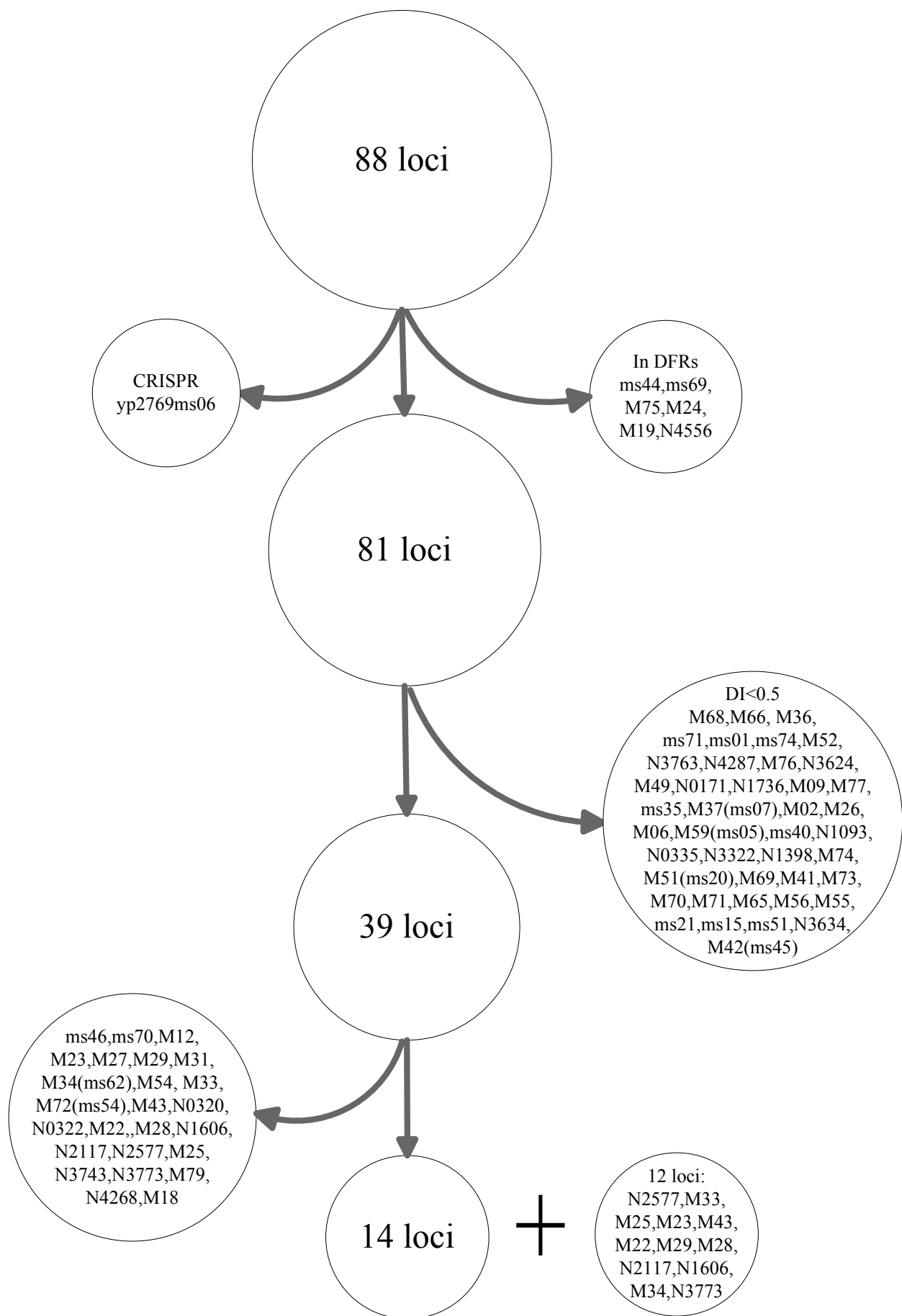

Supplement: Figure S3 — Procedure of locus selection for rapid genotyping system. (PDF) [file pone.0066567.s003.pdf]

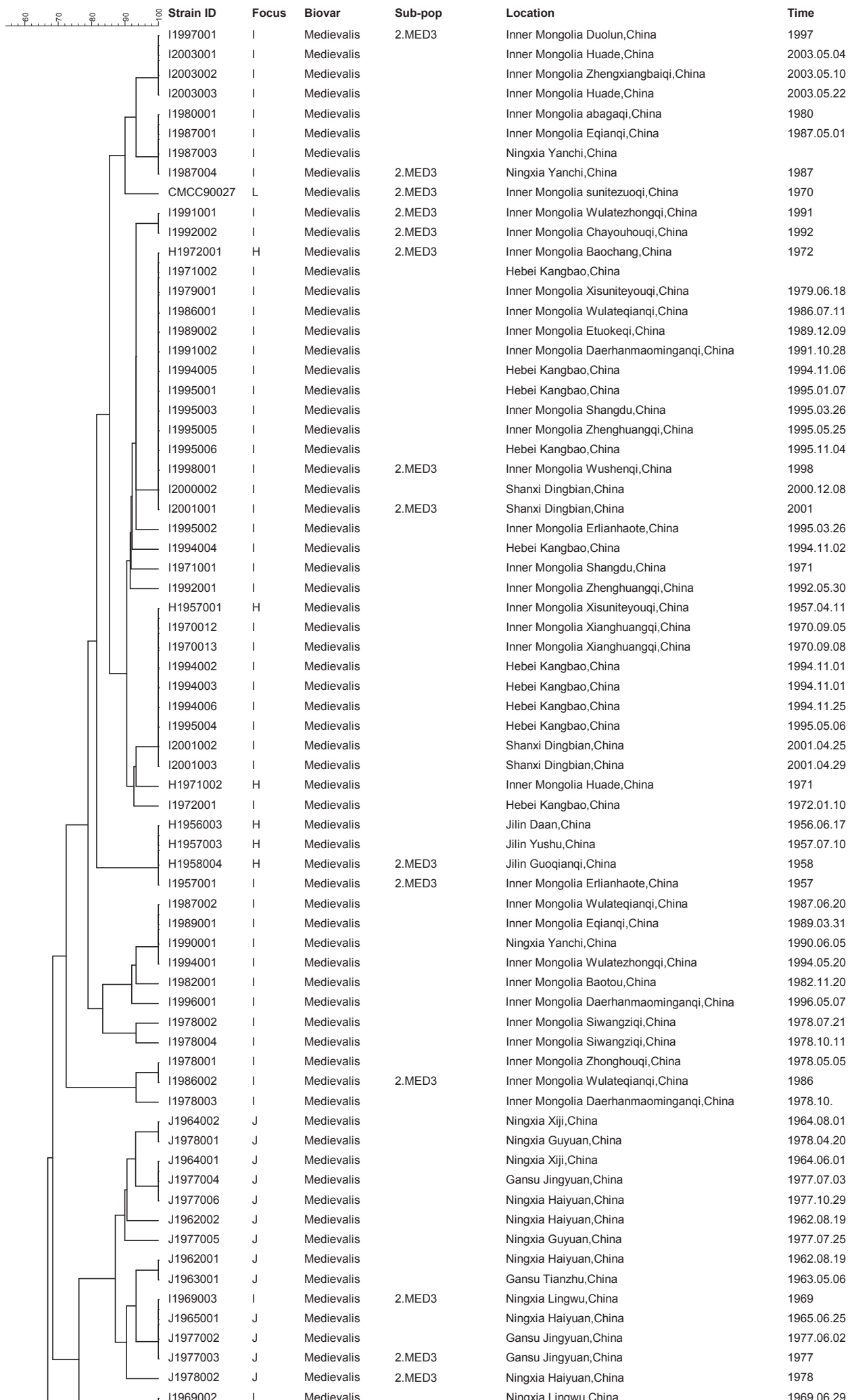

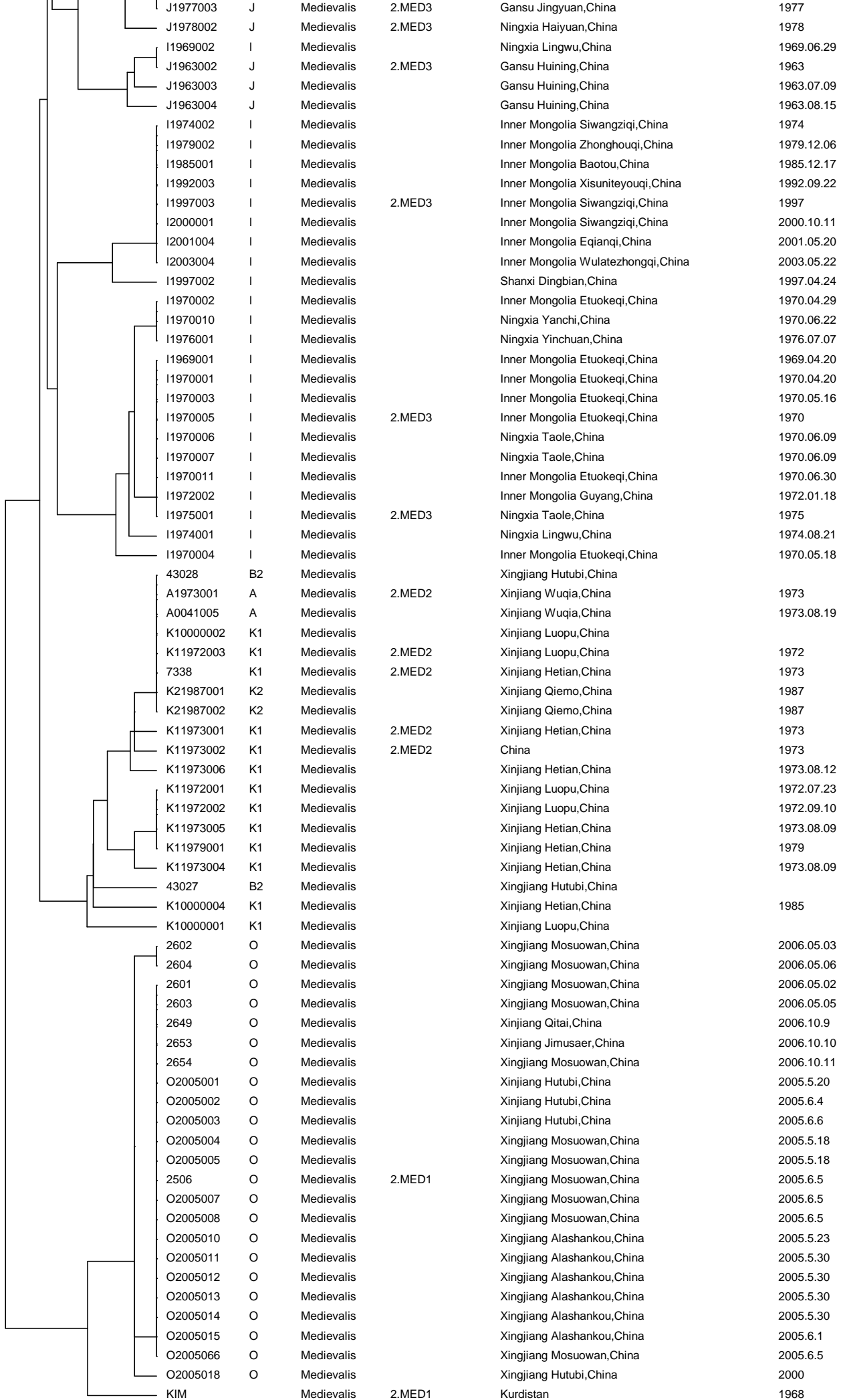

Supplement: Figure S5 — Dendrogram of Y. pestis strains clustered with 2.MED population based on 14+1 VNTR loci. A total of 139 strains were analyzed according to the profiles of 14 primary VNTRs and of the locus M25. (PDF) [file pone.0066567.s005.pdf]

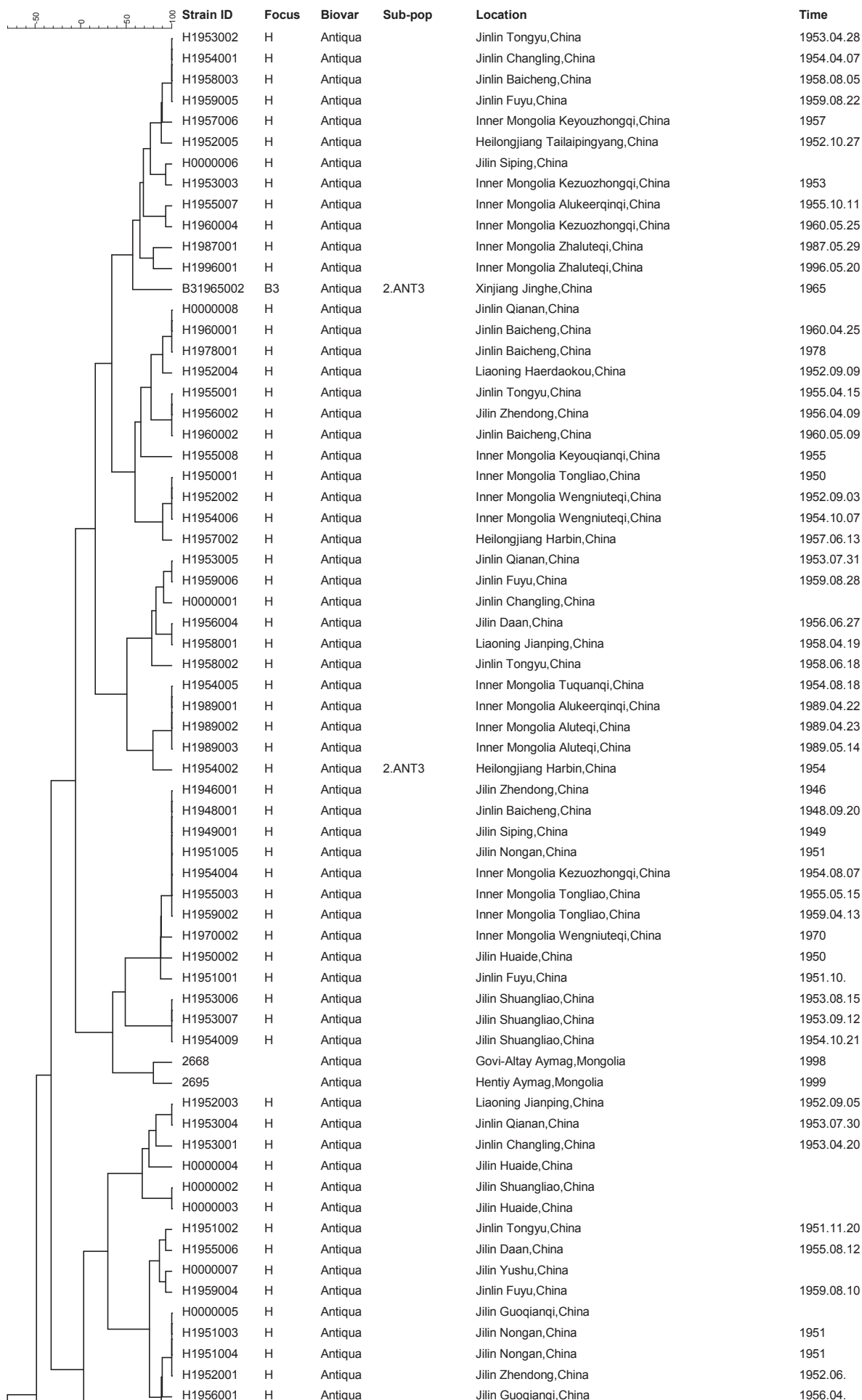

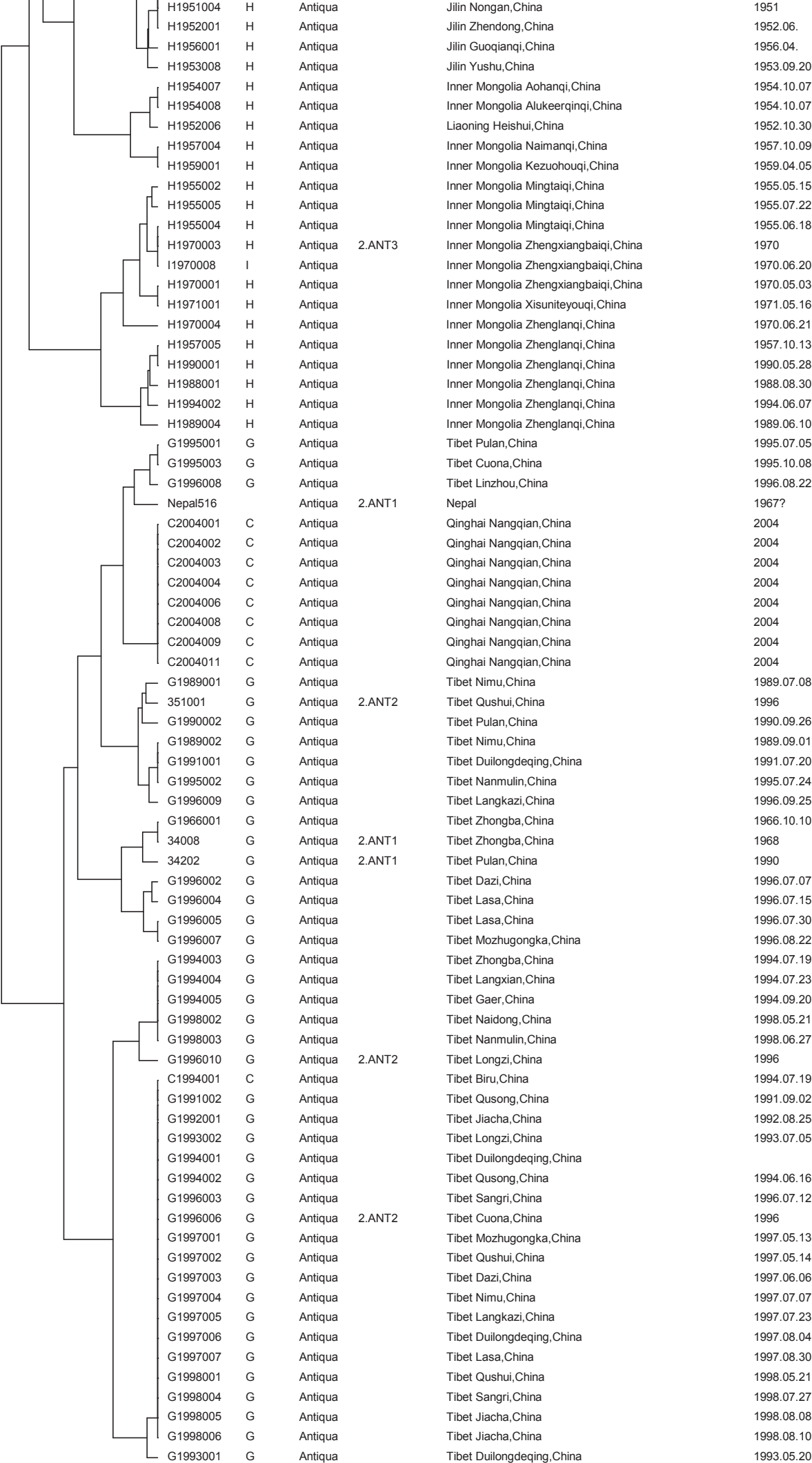

Supplement: Figure S6 — Dendrogram of Y. pestis strains clustered with 2.ANT population based on 14+1 VNTR loci. A total of 137 strains were analyzed according to the profiles of 14 primary VNTRs and the locus M25. (PDF) [file pone.0066567.s006.pdf]
