# Supplementary material for: Features of Variable Number of Tandem Repeats in Yersinia pestis and the Development of a Hierarchical Genotyping Scheme
Source: PLoS One. 2013 Jun 21;8(6):e66567. doi: 10.1371/journal.pone.0066567 (PMC3689786; doi:10.1371/journal.pone.0066567)
Supplement: Figure S4 — Dendrogram of 956 strains based on 14 primary VNTR loci. (PDF) [file pone.0066567.s004.pdf]

Categorical

956 strains with 14 loci

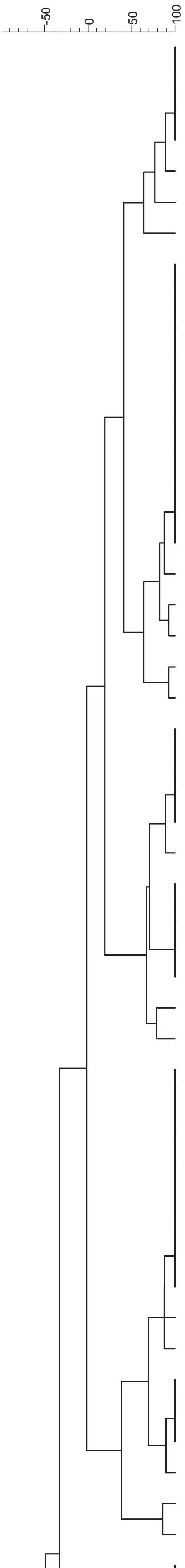

Strain ID	Focus	Biovar	Sub-pop	Location	Time
H0000008	H	Antiqua		Jinlin Qianan,China	
H1952004	H	Antiqua		Liaoning Haerdaokou,China	1952.09.09
H1960001	H	Antiqua		Jinlin Baicheng,China	1960.04.25
H1978001	H	Antiqua		Jinlin Baicheng,China	1978
H1951001	H	Antiqua		Jinlin Fuyu,China	1951.10.
H1955008	H	Antiqua		Inner Mongolia Keyouqianqi,China	1955
B31965002	B3	Antiqua	2.ANT3	Xinjiang Jinghe,China	1965
H1950001	H	Antiqua		Inner Mongolia Tongliao,China	1950
H1952002	H	Antiqua		Inner Mongolia Wengniuteqi,China	1952.09.03
H1953002	H	Antiqua		Jinlin Tongyu,China	1953.04.28
H1954001	H	Antiqua		Jinlin Changling,China	1954.04.07
H1954006	H	Antiqua		Inner Mongolia Wengniuteqi,China	1954.10.07
H1955001	H	Antiqua		Jinlin Tongyu,China	1955.04.15
H1956002	H	Antiqua		Jilin Zhendong,China	1956.04.09
H1958003	H	Antiqua		Jinlin Baicheng,China	1958.08.05
H1959005	H	Antiqua		Jinlin Fuyu,China	1959.08.22
H1960002	H	Antiqua		Jinlin Baicheng,China	1960.05.09
H1957006	H	Antiqua		Inner Mongolia Keyouzhongqi,China	1957
H1952005	H	Antiqua		Heilongjiang Tailaipingyang,China	1952.10.27
H1957002	H	Antiqua		Heilongjiang Harbin,China	1957.06.13
H1955007	H	Antiqua		Inner Mongolia Alukeerqinqi,China	1955.10.11
H1960004	H	Antiqua		Inner Mongolia Kezuozhongqi,China	1960.05.25
H1954005	H	Antiqua		Inner Mongolia Tuquanqi,China	1954.08.18
H1989001	H	Antiqua		Inner Mongolia Alukeerqinqi,China	1989.04.22
H1989002	H	Antiqua		Inner Mongolia Aluteqi,China	1989.04.23
H1989003	H	Antiqua		Inner Mongolia Aluteqi,China	1989.05.14
H1954002	H	Antiqua	2.ANT3	Heilongjiang Harbin,China	1954
H1953005	H	Antiqua		Jinlin Qianan,China	1953.07.31
H1956004	H	Antiqua		Jilin Daan,China	1956.06.27
H1958001	H	Antiqua		Liaoning Jianping,China	1958.04.19
H1959006	H	Antiqua		Jinlin Fuyu,China	1959.08.28
H1987001	H	Antiqua		Inner Mongolia Zhaluteqi,China	1987.05.29
H1996001	H	Antiqua		Inner Mongolia Zhaluteqi,China	1996.05.20
H0000006	H	Antiqua		Jilin Siping,China	
H1946001	H	Antiqua		Jilin Zhendong,China	1946
H1948001	H	Antiqua		Jinlin Baicheng,China	1948.09.20
H1949001	H	Antiqua		Jilin Siping,China	1949
H1951005	H	Antiqua		Jilin Nongan,China	1951
H1954004	H	Antiqua		Inner Mongolia Kezuozhongqi,China	1954.08.07
H1955003	H	Antiqua		Inner Mongolia Tongliao,China	1955.05.15
H1959002	H	Antiqua		Inner Mongolia Tongliao,China	1959.04.13
H1970002	H	Antiqua		Inner Mongolia Wengniuteqi,China	1970
H1950002	H	Antiqua		Jilin Huaide,China	1950
H1953006	H	Antiqua		Jilin Shuangliao,China	1953.08.15
H1953007	H	Antiqua		Jilin Shuangliao,China	1953.09.12
H1954009	H	Antiqua		Jilin Shuangliao,China	1954.10.21
H1953003	H	Antiqua		Inner Mongolia Kezuozhongqi,China	1953
2668		Antiqua		Govi-Altay Aymag,Mongolia	1998
2695		Antiqua		Hentiy Aymag,Mongolia	1999
H0000005	H	Antiqua		Jilin Guoqianqi,China	

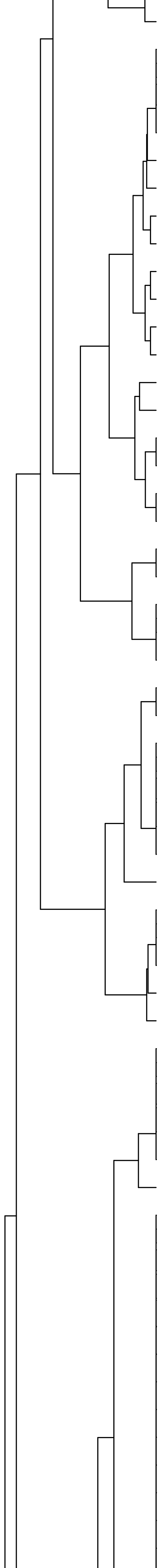

[illegible]				[illegible]	
2695		Antiqua		Hentiy Aymag,Mongolia	1999
H0000005	H	Antiqua		Jilin Guoqianqi,China	
H1951003	H	Antiqua		Jilin Nongan,China	1951
H1951004	H	Antiqua		Jilin Nongan,China	1951
H1952001	H	Antiqua		Jilin Zhendong,China	1952.06.
H1956001	H	Antiqua		Jilin Guoqianqi,China	1956.04.
H1953008	H	Antiqua		Jilin Yushu,China	1953.09.20
H0000001	H	Antiqua		Jinlin Changling,China	
H1958002	H	Antiqua		Jinlin Tongyu,China	1958.06.18
H1951002	H	Antiqua		Jinlin Tongyu,China	1951.11.20
H1955006	H	Antiqua		Jilin Daan,China	1955.08.12
H0000007	H	Antiqua		Jilin Yushu,China	
H1959004	H	Antiqua		Jinlin Fuyu,China	1959.08.10
H0000004	H	Antiqua		Jilin Huaide,China	
H1953001	H	Antiqua		Jinlin Changling,China	1953.04.20
H1952003	H	Antiqua		Liaoning Jianping,China	1952.09.05
H1953004	H	Antiqua		Jinlin Qianan,China	1953.07.30
H0000002	H	Antiqua		Jilin Shuangliao,China	
H0000003	H	Antiqua		Jilin Huaide,China	
H1957004	H	Antiqua		Inner Mongolia Naimanqi,China	1957.10.09
H1959001	H	Antiqua		Inner Mongolia Kezuohouqi,China	1959.04.05
H1952006	H	Antiqua		Liaoning Heishui,China	1952.10.30
H1954007	H	Antiqua		Inner Mongolia Aohanqi,China	1954.10.07
H1954008	H	Antiqua		Inner Mongolia Alukeerqinqi,China	1954.10.07
H1970001	H	Antiqua		Inner Mongolia Zhengxiangbaiqi,China	1970.05.03
H1971001	H	Antiqua		Inner Mongolia Xisuniteyouqi,China	1971.05.16
H1955002	H	Antiqua		Inner Mongolia Mingtaiqi,China	1955.05.15
H1955004	H	Antiqua		Inner Mongolia Mingtaiqi,China	1955.06.18
H1955005	H	Antiqua		Inner Mongolia Mingtaiqi,China	1955.07.22
H1970003	H	Antiqua	2.ANT3	Inner Mongolia Zhengxiangbaiqi,China	1970
I1970008	I	Antiqua		Inner Mongolia Zhengxiangbaiqi,China	1970.06.20
H1970004	H	Antiqua		Inner Mongolia Zhenglanqi,China	1970.06.21
H1957005	H	Antiqua		Inner Mongolia Zhenglanqi,China	1957.10.13
H1988001	H	Antiqua		Inner Mongolia Zhenglanqi,China	1988.08.30
H1990001	H	Antiqua		Inner Mongolia Zhenglanqi,China	1990.05.28
H1989004	H	Antiqua		Inner Mongolia Zhenglanqi,China	1989.06.10
H1994002	H	Antiqua		Inner Mongolia Zhenglanqi,China	1994.06.07
G1994003	G	Antiqua		Tibet Zhongba,China	1994.07.19
G1994004	G	Antiqua		Tibet Langxian,China	1994.07.23
G1994005	G	Antiqua		Tibet Gaer,China	1994.09.20
G1998002	G	Antiqua		Tibet Naidong,China	1998.05.21
G1998003	G	Antiqua		Tibet Nanmulin,China	1998.06.27
G1996010	G	Antiqua	2.ANT2	Tibet Longzi,China	1996
C1994001	C	Antiqua		Tibet Biru,China	1994.07.19
G1991002	G	Antiqua		Tibet Qusong,China	1991.09.02
G1992001	G	Antiqua		Tibet Jiacha,China	1992.08.25
G1993002	G	Antiqua		Tibet Longzi,China	1993.07.05
G1994001	G	Antiqua		Tibet Duilongdeqing,China	
G1994002	G	Antiqua		Tibet Qusong,China	1994.06.16
G1996003	G	Antiqua		Tibet Sangri,China	1996.07.12
G1996006	G	Antiqua	2.ANT2	Tibet Cuona,China	1996
G1997001	G	Antiqua		Tibet Mozhugongka,China	1997.05.13
G1997002	G	Antiqua		Tibet Qushui,China	1997.05.14
G1997003	G	Antiqua		Tibet Dazi,China	1997.06.06
G1997004	G	Antiqua		Tibet Nimu,China	1997.07.07
G1997005	G	Antiqua		Tibet Langkazi,China	1997.07.23
[illegible]				[illegible]	[illegible]

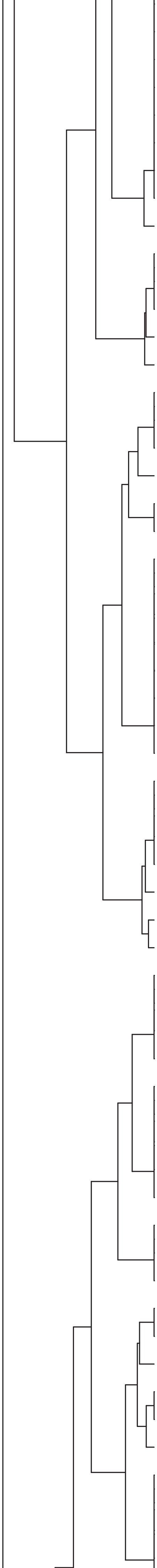

G1997004	G	Antiqua		Tibet Nimu,China	1997.07.07
G1997005	G	Antiqua		Tibet Langkazi,China	1997.07.23
G1997006	G	Antiqua		Tibet Duilongdeqing,China	1997.08.04
G1997007	G	Antiqua		Tibet Lasa,China	1997.08.30
G1998001	G	Antiqua		Tibet Qushui,China	1998.05.21
G1998004	G	Antiqua		Tibet Sangri,China	1998.07.27
G1998005	G	Antiqua		Tibet Jiacha,China	1998.08.08
G1998006	G	Antiqua		Tibet Jiacha,China	1998.08.10
G1993001	G	Antiqua		Tibet Duilongdeqing,China	1993.05.20
G1996004	G	Antiqua		Tibet Lasa,China	1996.07.15
G1996005	G	Antiqua		Tibet Lasa,China	1996.07.30
G1996007	G	Antiqua		Tibet Mozhugongka,China	1996.08.22
34202	G	Antiqua	2.ANT1	Tibet Pulan,China	1990
G1996002	G	Antiqua		Tibet Dazi,China	1996.07.07
G1995001	G	Antiqua		Tibet Pulan,China	1995.07.05
G1995003	G	Antiqua		Tibet Cuona,China	1995.10.08
G1996008	G	Antiqua		Tibet Linzhou,China	1996.08.22
Nepal516		Antiqua	2.ANT1	Nepal	1967?
G1966001	G	Antiqua		Tibet Zhongba,China	1966.10.10
34008	G	Antiqua	2.ANT1	Tibet Zhongba,China	1968
C2004001	C	Antiqua		Qinghai Nangqian,China	2004
C2004002	C	Antiqua		Qinghai Nangqian,China	2004
C2004003	C	Antiqua		Qinghai Nangqian,China	2004
C2004004	C	Antiqua		Qinghai Nangqian,China	2004
C2004006	C	Antiqua		Qinghai Nangqian,China	2004
C2004008	C	Antiqua		Qinghai Nangqian,China	2004
C2004009	C	Antiqua		Qinghai Nangqian,China	2004
C2004011	C	Antiqua		Qinghai Nangqian,China	2004
G1989002	G	Antiqua		Tibet Nimu,China	1989.09.01
G1991001	G	Antiqua		Tibet Duilongdeqing,China	1991.07.20
G1995002	G	Antiqua		Tibet Nanmulin,China	1995.07.24
G1996009	G	Antiqua		Tibet Langkazi,China	1996.09.25
351001	G	Antiqua	2.ANT2	Tibet Qushui,China	1996
G1989001	G	Antiqua		Tibet Nimu,China	1989.07.08
G1990002	G	Antiqua		Tibet Pulan,China	1990.09.26
I1997001	I	Medievalis	2.MED3	Inner Mongolia Duolun,China	1997
I2003001	I	Medievalis		Inner Mongolia Huade,China	2003.05.04
I2003002	I	Medievalis		Inner Mongolia Zhengxiangbaiqi,China	2003.05.10
I2003003	I	Medievalis		Inner Mongolia Huade,China	2003.05.22
I1980001	I	Medievalis		Inner Mongolia abagaqi,China	1980
I1987001	I	Medievalis		Inner Mongolia Eqianqi,China	1987.05.01
I1987003	I	Medievalis		Ningxia Yanchi,China	
I1987004	I	Medievalis	2.MED3	Ningxia Yanchi,China	1987
CMCC90027	L	Medievalis	2.MED3	Inner Mongolia sunitezuoqi,China	1970
I1978001	I	Medievalis		Inner Mongolia Zhonghouqi,China	1978.05.05
I1978003	I	Medievalis		Inner Mongolia Daerhanmaominganqi,Chi.	1978.10.
I1986002	I	Medievalis	2.MED3	Inner Mongolia Wulateqianqi,China	1986
I1991001	I	Medievalis	2.MED3	Inner Mongolia Wulatezhongqi,China	1991
I1992002	I	Medievalis	2.MED3	Inner Mongolia Chayouhouqi,China	1992
K10000004	K1	Medievalis		Xinjiang Hetian,China	1985
I1982001	I	Medievalis		Inner Mongolia Baotou,China	1982.11.20
I1995002	I	Medievalis		Inner Mongolia Erlianhaote,China	1995.03.26
J1978002	J	Medievalis	2.MED3	Ningxia Haiyuan,China	1978
I1987002	I	Medievalis		Inner Mongolia Wulateqianqi,China	1987.06.20
I1989001	I	Medievalis		Inner Mongolia Eqianqi,China	1989.03.31
I1990001	I	Medievalis		Ningxia Yanchi,China	1990.06.05
I1994001	I	Medievalis		Inner Mongolia Wulatezhongqi,China	1994.05.20

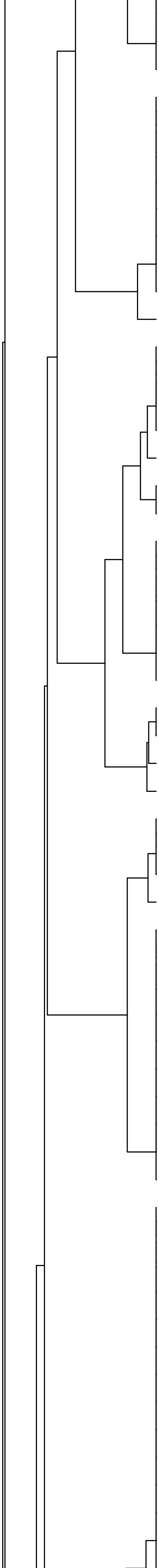

I1990001	I	Medievalis		Ningxia Yanchi,China	1990.06.05
I1994001	I	Medievalis		Inner Mongolia Wulatezhongqi,China	1994.05.20
I1996001	I	Medievalis		Inner Mongolia Daerhanmaominganqi,Chi.	1996.05.07
I1974002	I	Medievalis		Inner Mongolia Siwangziqi,China	1974
I1979002	I	Medievalis		Inner Mongolia Zhonghouqi,China	1979.12.06
I1985001	I	Medievalis		Inner Mongolia Baotou,China	1985.12.17
I1992003	I	Medievalis		Inner Mongolia Xisuniteyouqi,China	1992.09.22
I1997003	I	Medievalis	2.MED3	Inner Mongolia Siwangziqi,China	1997
I2000001	I	Medievalis		Inner Mongolia Siwangziqi,China	2000.10.11
I2001004	I	Medievalis		Inner Mongolia Eqianqi,China	2001.05.20
I2003004	I	Medievalis		Inner Mongolia Wulatezhongqi,China	2003.05.22
I1997002	I	Medievalis		Shanxi Dingbian,China	1997.04.24
J1962002	J	Medievalis		Ningxia Haiyuan,China	1962.08.19
J1964001	J	Medievalis		Ningxia Xiji,China	1964.06.01
J1977004	J	Medievalis		Gansu Jingyuan,China	1977.07.03
J1977006	J	Medievalis		Ningxia Haiyuan,China	1977.10.29
J1977005	J	Medievalis		Ningxia Guyuan,China	1977.07.25
J1964002	J	Medievalis		Ningxia Xiji,China	1964.08.01
J1978001	J	Medievalis		Ningxia Guyuan,China	1978.04.20
I1969003	I	Medievalis	2.MED3	Ningxia Lingwu,China	1969
J1962001	J	Medievalis		Ningxia Haiyuan,China	1962.08.19
J1963001	J	Medievalis		Gansu Tianzhu,China	1963.05.06
J1965001	J	Medievalis		Ningxia Haiyuan,China	1965.06.25
J1977002	J	Medievalis		Gansu Jingyuan,China	1977.06.02
J1977003	J	Medievalis	2.MED3	Gansu Jingyuan,China	1977
I1969002	I	Medievalis		Ningxia Lingwu,China	1969.06.29
J1963002	J	Medievalis	2.MED3	Gansu Huining,China	1963
J1963003	J	Medievalis		Gansu Huining,China	1963.07.09
J1963004	J	Medievalis		Gansu Huining,China	1963.08.15
I1970002	I	Medievalis		Inner Mongolia Etuokeqi,China	1970.04.29
I1970010	I	Medievalis		Ningxia Yanchi,China	1970.06.22
I1976001	I	Medievalis		Ningxia Yinchuan,China	1976.07.07
I1970004	I	Medievalis		Inner Mongolia Etuokeqi,China	1970.05.18
I1969001	I	Medievalis		Inner Mongolia Etuokeqi,China	1969.04.20
I1970001	I	Medievalis		Inner Mongolia Etuokeqi,China	1970.04.20
I1970003	I	Medievalis		Inner Mongolia Etuokeqi,China	1970.05.16
I1970005	I	Medievalis	2.MED3	Inner Mongolia Etuokeqi,China	1970
I1970006	I	Medievalis		Ningxia Taole,China	1970.06.09
I1970007	I	Medievalis		Ningxia Taole,China	1970.06.09
I1970011	I	Medievalis		Inner Mongolia Etuokeqi,China	1970.06.30
I1972002	I	Medievalis		Inner Mongolia Guyang,China	1972.01.18
I1974001	I	Medievalis		Ningxia Lingwu,China	1974.08.21
I1975001	I	Medievalis	2.MED3	Ningxia Taole,China	1975
H1972001	H	Medievalis	2.MED3	Inner Mongolia Baochang,China	1972
I1971002	I	Medievalis		Hebei Kangbao,China	
I1979001	I	Medievalis		Inner Mongolia Xisuniteyouqi,China	1979.06.18
I1986001	I	Medievalis		Inner Mongolia Wulateqianqi,China	1986.07.11
I1989002	I	Medievalis		Inner Mongolia Etuokeqi,China	1989.12.09
I1991002	I	Medievalis		Inner Mongolia Daerhanmaominganqi,Chi.	1991.10.28
I1994005	I	Medievalis		Hebei Kangbao,China	1994.11.06
I1995001	I	Medievalis		Hebei Kangbao,China	1995.01.07
I1995003	I	Medievalis		Inner Mongolia Shangdu,China	1995.03.26
I1995005	I	Medievalis		Inner Mongolia Zhenghuangqi,China	1995.05.25
I1995006	I	Medievalis		Hebei Kangbao,China	1995.11.04
I1998001	I	Medievalis	2.MED3	Inner Mongolia Wushenqi,China	1998
I2000002	I	Medievalis		Shanxi Dingbian,China	2000.12.08
I2001001	I	Medievalis	2.MED3	Shanxi Dingbian,China	2001

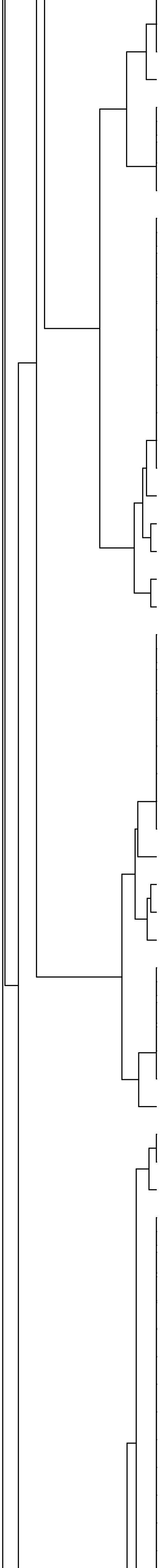

[illegible]	[illegible]	[illegible]	[illegible]	[illegible]	[illegible]
I2000002	I	Medievalis		Shanxi Dingbian,China	2000.12.08
I2001001	I	Medievalis	2.MED3	Shanxi Dingbian,China	2001
I1992001	I	Medievalis		Inner Mongolia Zhenghuangqi,China	1992.05.30
H1956003	H	Medievalis		Jilin Daan,China	1956.06.17
H1957003	H	Medievalis		Jilin Yushu,China	1957.07.10
H1958004	H	Medievalis	2.MED3	Jilin Guoqianqi,China	1958
I1957001	I	Medievalis	2.MED3	Inner Mongolia Erlianhaote,China	1957
H1957001	H	Medievalis		Inner Mongolia Xisuniteyouqi,China	1957.04.11
I1970012	I	Medievalis		Inner Mongolia Xianghuangqi,China	1970.09.05
I1970013	I	Medievalis		Inner Mongolia Xianghuangqi,China	1970.09.08
I1972001	I	Medievalis		Hebei Kangbao,China	1972.01.10
I1994002	I	Medievalis		Hebei Kangbao,China	1994.11.01
I1994003	I	Medievalis		Hebei Kangbao,China	1994.11.01
I1994006	I	Medievalis		Hebei Kangbao,China	1994.11.25
I1995004	I	Medievalis		Hebei Kangbao,China	1995.05.06
I2001002	I	Medievalis		Shanxi Dingbian,China	2001.04.25
I2001003	I	Medievalis		Shanxi Dingbian,China	2001.04.29
I1994004	I	Medievalis		Hebei Kangbao,China	1994.11.02
I1978002	I	Medievalis		Inner Mongolia Siwangziqi,China	1978.07.21
I1978004	I	Medievalis		Inner Mongolia Siwangziqi,China	1978.10.11
H1971002	H	Medievalis		Inner Mongolia Huade,China	1971
I1971001	I	Medievalis		Inner Mongolia Shangdu,China	1971
43028	B2	Medievalis		Xingjiang Hutubi,China	
A1973001	A	Medievalis	2.MED2	Xinjiang Wuqia,China	1973
A0041005	A	Medievalis		Xinjiang Wuqia,China	1973.08.19
K10000002	K1	Medievalis		Xinjiang Luopu,China	
K11972003	K1	Medievalis	2.MED2	Xinjiang Luopu,China	1972
7338	K1	Medievalis	2.MED2	Xinjiang Hetian,China	1973
K21987001	K2	Medievalis		Xinjiang Qiemo,China	1987
K21987002	K2	Medievalis		Xinjiang Qiemo,China	1987
43027	B2	Medievalis		Xingjiang Hutubi,China	
K11973001	K1	Medievalis	2.MED2	Xinjiang Hetian,China	1973
K11973004	K1	Medievalis		Xinjiang Hetian,China	1973.08.09
K11973002	K1	Medievalis	2.MED2	China	1973
K11972001	K1	Medievalis		Xinjiang Luopu,China	1972.07.23
K11972002	K1	Medievalis		Xinjiang Luopu,China	1972.09.10
K11973005	K1	Medievalis		Xinjiang Hetian,China	1973.08.09
K11973006	K1	Medievalis		Xinjiang Hetian,China	1973.08.12
K11979001	K1	Medievalis		Xinjiang Hetian,China	1979
K10000001	K1	Medievalis		Xinjiang Luopu,China	
2602	O	Medievalis		Xingjiang Mosuowan,China	2006.05.03
2604	O	Medievalis		Xingjiang Mosuowan,China	2006.05.06
O2005018	O	Medievalis		Xingjiang Hutubi,China	2000
2601	O	Medievalis		Xingjiang Mosuowan,China	2006.05.02
2603	O	Medievalis		Xingjiang Mosuowan,China	2006.05.05
2649	O	Medievalis		Xinjiang Qitai,China	2006.10.9
2653	O	Medievalis		Xinjiang Jimusaer,China	2006.10.10
2654	O	Medievalis		Xingjiang Mosuowan,China	2006.10.11
O2005001	O	Medievalis		Xinjiang Hutubi,China	2005.5.20
O2005002	O	Medievalis		Xinjiang Hutubi,China	2005.6.4
O2005003	O	Medievalis		Xinjiang Hutubi,China	2005.6.6
O2005004	O	Medievalis		Xingjiang Mosuowan,China	2005.5.18
O2005005	O	Medievalis		Xingjiang Mosuowan,China	2005.5.18
2506	O	Medievalis	2.MED1	Xingjiang Mosuowan,China	2005.6.5
O2005007	O	Medievalis		Xingjiang Mosuowan,China	2005.6.5
O2005008	O	Medievalis		Xingjiang Mosuowan,China	2005.6.5

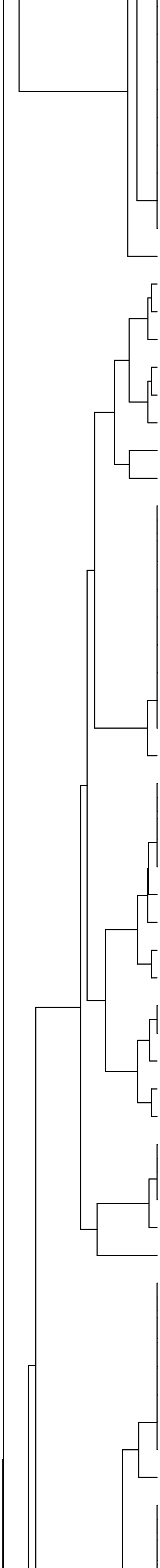

O2005007	O	Medievalis		Xingjiang Mosuowan,China	2005.6.5
O2005008	O	Medievalis		Xingjiang Mosuowan,China	2005.6.5
O2005010	O	Medievalis		Xingjiang Alashankou,China	2005.5.23
O2005011	O	Medievalis		Xingjiang Alashankou,China	2005.5.30
O2005012	O	Medievalis		Xingjiang Alashankou,China	2005.5.30
O2005013	O	Medievalis		Xingjiang Alashankou,China	2005.5.30
O2005014	O	Medievalis		Xingjiang Alashankou,China	2005.5.30
O2005015	O	Medievalis		Xingjiang Alashankou,China	2005.6.1
O2005066	O	Medievalis		Xingjiang Mosuowan,China	2005.6.5
KIM		Medievalis	2.MED1	Kurdistan	1968
41022	A	Antiqua		Xinjiang Wuqia,China	
B11995002	B1	Antiqua		Xinjiang Atushi,China	1995.08.08
B20003001	B2	Antiqua		Xinjiang Wulumuqi,China	2000
B11979001	B1	Antiqua		Xinjiang Atushi,China	1979
B11992002	B1	Antiqua		Xinjiang Atushi,China	1992.08.26
B11995001	B1	Antiqua		Xinjiang Atushi,China	1995.08.08
757		Antiqua		Hentiy Aymag,Mongolia	1981
3056		Antiqua		Bayan-Uul,Mongolia	2003
A0041019	A	Antiqua		Xinjiang Wuqia,China	
A1996002	A	Antiqua		Xinjiang Wuqia,China	1996.08.04
A1996003	A	Antiqua		Xinjiang Wuqia,China	1996.08.08
B11989001	B1	Antiqua		Xinjiang Wuqia,China	1989.08.14
B11989002	B1	Antiqua		Xinjiang Atushi,China	1989.09.11
B11989003	B1	Antiqua		Xinjiang Wuqia,China	1989.09.11
B11997001	B1	Antiqua		Xinjiang Wuqia,China	1997.06.29
B12001001	B1	Antiqua		Xinjiang Wuqia,China	2001.07.18
B12002001	B1	Antiqua		Xinjiang Wuqia,China	2002.07.16
A1992001	A	Antiqua	0.ANT3	Xinjiang Wuqia,China	1992
A1995001	A	Antiqua		Xinjiang Wuqia,China	1995.07.06
A1996001	A	Antiqua		Xinjiang Wuqia,China	1996.07.07
A1998001	A	Antiqua		Xinjiang Wuqia,China	1998.07.18
A1998002	A	Antiqua		Xinjiang Wuqia,China	1998.07.18
A1997001	A	Antiqua		Xinjiang Wuqia,China	1997.03.31
A1983001	A	Antiqua		Xinjiang Wuqia,China	1983.09.17
A1956001	A	Antiqua	0.ANT3	Xinjiang Wuqia,China	1956
A1960001	A	Antiqua		Xinjiang Wuqia,China	1960.08.28
B30000001	B4	Antiqua		Xinjiang Wenquan,China	
B42003002	B4	Antiqua		Xinjiang Wenquan,China	2003.07.24
B42003001	B4	Antiqua		Xinjiang Wenquan,China	2003.07.15
2330	B2	Antiqua	0.ANT2	Xinjiang Wenquan,China	2003
B42003004	B4	Antiqua	0.ANT2	China	2003
B11965001	B1	Antiqua		Xinjiang Aheqi,China	1965.07.01
B11992001	B1	Antiqua		Xinjiang Aheqi,China	1992.07.18
B11996002	B1	Antiqua		Xinjiang Aheqi,China	1996.08.17
B11996001	B1	Antiqua		Xinjiang Aheqi,China	1996.08.17
PestoidesF		Microtus	0.PE2	USSR	<1984
B31978001	B3	Antiqua		Xinjiang Wusu,China	1978.09.08
B31989001	B3	Antiqua		Xinjiang Wusu,China	1989.07.24
B31989003	B3	Antiqua		Xinjiang Shawan,China	1989.09.18
B31994001	B3	Antiqua		Xinjiang Wusu,China	1994.06.30
B32002006	B3	Antiqua		Xinjiang Wusu,China	2002.09.05
B32002008	B3	Antiqua		Xinjiang Shawan,China	2002.08.23
B32003001	B3	Antiqua		Xinjiang Wusu,China	2003.06.09
B32002002	B3	Antiqua		Xinjiang Wusu,China	2002
2655	O	Antiqua		Xinjiang Kelamayi,China	2006.10.9
B21992001	B2	Antiqua		Xinjiang Hutubi,China	1992.08.26
B31988002	B3	Antiqua		Xinjiang Shawan,China	1988.08.10

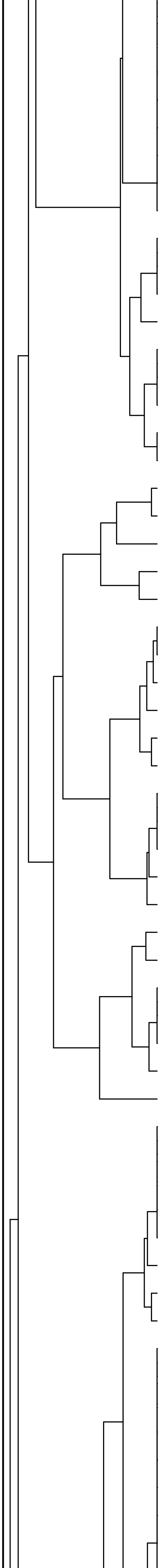

B21992001	B2	Antiqua		Xinjiang Hutubi,China	1992.08.26
B31988002	B3	Antiqua		Xinjiang Shawan,China	1988.08.10
B31988003	B3	Antiqua		Xinjiang Shawan,China	1988.08.12
B31989004	B3	Antiqua		Xinjiang Shawan,China	1993.09.01
945	B3	Antiqua	0.ANT1	Xinjiang Shawan,China	1994
B32002001	B3	Antiqua		Xinjiang Wusu,China	2002.07.02
B32002003	B3	Antiqua		Xinjiang Shawan,China	2002.08.08
B32002004	B3	Antiqua		Xinjiang Shawan,China	2002.08.08
B22002005	B2	Antiqua		Xinjiang Manasi,China	2002.09.05
B32001006	B3	Antiqua		Xinjiang Shawan,China	2001.08.17
B32002005	B3	Antiqua		Xinjiang Shawan,China	2002.08.08
B32001005	B3	Antiqua		Xinjiang Shawan,China	2001.08.17
B31988004	B3	Antiqua		Xinjiang Shawan,China	1988
B31989005	B3	Antiqua		Xinjiang Shawan,China	1989
B32002016	B3	Antiqua		Xinjiang Shawan,China	2002.08.08
B22002006	B2	Antiqua		Xinjiang Manasi,China	2002.09.05
B32001007	B3	Antiqua		Xinjiang Shawan,China	2001.08.17
C1961001	C	Antiqua		Qinghai Xinghai,China	1961.05.11
620024	C	Antiqua	0.PE7	Qinghai Xinghai,China	1962
PestoidesA		Microtus	0.PE4	FSU	NA
Antiqua		Aantiqua	1.ANT1	Congo	1965
UG05-0454		Aantiqua	1.ANT1	Uganda	2004
342		Antiqua		South-Gobi Aymag,Mongolia	1977
629		Antiqua		Hovd Aymag,Mongolia	1980
2873		Antiqua		Bayan-Uul,Mongolia	2000
1387		Microtus		South-Gobi Aymag,Mongolia	1986
956		Antiqua		Hentiy Aymag,Mongolia	1981
988		Antiqua		Hentiy Aymag,Mongolia	1983
621		Antiqua		Govi-Altay Aymag,Mongolia	1980
2641		Antiqua		East Goby Aymag,Mongolia	1997
3086		Antiqua		Govi-Altay Aymag,Mongolia	2003
2669		Antiqua		Govi-Altay Aymag,Mongolia	1998
2642		Antiqua		East Goby Aymag,Mongolia	1997
C1972001	C	Antiqua	3.ANT1	Qinghai Lenghu,China	1972
C1972002	C	Antiqua		Qinghai Lenghu,China	1972
C1961006	C	Antiqua		Gansu Akesai,China	1961.07.20
C1976001	C	Antiqua		Gansu Akesai,China	1976.06.12
C1989002	C	Antiqua		Gansu Akesai,China	1989.08.14
C1972003	C	Antiqua		Qinghai Lenghu,China	1972.06.22
Angola		Microtus	0.PE3	Angola	<1984
B2040040	B2	Antiqua		Xinjiang Manasi,China	
B21972003	B2	Antiqua		Xinjiang Manasi,China	1972.08.02
B21984001	B2	Antiqua		Xinjiang Changji,China	1984.08.08
B21997001	B2	Antiqua		Xinjiang Manasi,China	1997.07.26
B31967002	B3	Antiqua		Xinjiang Wusu,China	1967.08.08
B31972001	B3	Antiqua		Xinjiang Jinghe,China	1972.07.06
B41999002	B4	Antiqua		Xinjiang Yining,China	1999.07.12
K21985001	K2	Antiqua		Xinjiang Ruoqiang,China	1985.07.06
B21959002	B2	Antiqua		Xinjiang Hutubi,China	1959.09.12
B21960001	B2	Antiqua		Xinjiang Changji,China	1960.08.15
B21980003	B2	Antiqua		Xinjiang Hutubi,China	1980.08.28
B21983004	B2	Antiqua		Xinjiang Wulumuqi,China	1983.08.20
B21984002	B2	Antiqua		Xinjiang Changji,China	1984.08.08
B21989001	B2	Antiqua		Xinjiang Changji,China	1989.09.28
B21990001	B2	Antiqua		Xinjiang Wulumuqi,China	1990.05.23
B22000002	B2	Antiqua		Xinjiang Hutubi,China	2000.07.29
B31999002	B3	Antiqua		Xinjiang Wusu,China	1999.08.06

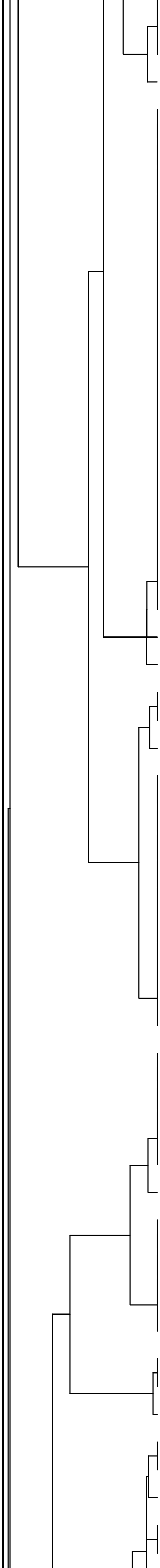

[illegible]	B2	Antiqua		Xinjiang Wulumuqi,China	[illegible]
B22000002	B2	Antiqua		Xinjiang Hutubi,China	2000.07.29
B31999002	B3	Antiqua		Xinjiang Wusu,China	1999.08.06
43013	B2	Antiqua		Xingjiang Hutubi,China	
44034	B2	Antiqua		Xinjiang Changji,China	
B2040060	B2	Antiqua		Xinjiang Manasi,China	
B2040061	B2	Antiqua		Xinjiang Manasi,China	
B21959001	B2	Antiqua	0.ANT1	Xinjiang Manasi,China	1959
B21961001	B2	Antiqua		Xinjiang Manasi,China	1961.08.19
B21972001	B2	Antiqua		Xinjiang Hutubi,China	1972.07.21
B21979001	B2	Antiqua		Xinjiang Changji,China	1979.08.10
B21980001	B2	Antiqua		Xinjiang Hutubi,China	1980.08.03
B21980002	B2	Antiqua		Xinjiang Hutubi,China	1980.08.07
B21983001	B2	Antiqua		Xinjiang Changji,China	1983
B21983002	B2	Antiqua		Xinjiang Wulumuqi,China	1983.07.16
B21983003	B2	Antiqua		Xinjiang Wulumuqi,China	1983.07.16
B21984003	B2	Antiqua	0.ANT1	Xinjiang Changji,China	1984
B21987001	B2	Antiqua		Xinjiang Manasi,China	1987.06.12
B21987002	B2	Antiqua		Xinjiang Manasi,China	1987.06.18
B21990002	B2	Antiqua		Xinjiang Wulumuqi,China	1990.05.30
CMCC8211	B3	Antiqua	0.ANT1	Xinjiang Jinghe,China	1982
B41999001	B4	Antiqua		Xinjiang Yining,China	1999.07.12
K21985005	K2	Antiqua		Xinjiang Ruoqiang,China	1985
B32001003	B3	Antiqua		Xinjiang Wusu,China	2001.08.01
B21965001	B2	Antiqua		Xinjiang Manasi,China	1965.08.01
44036	B2	Antiqua		Xinjiang Changji,China	
B22002004	B2	Antiqua		Xinjiang Changji,China	2002.07.22
B22002002	B2	Antiqua		Xinjiang Changji,China	2002.07.22
B20003002	B2	Antiqua		Xinjiang Wulumuqi,China	2000
B20003003	B2	Antiqua		Xinjiang Wulumuqi,China	2000
B21999001	B2	Antiqua		Xinjiang Changji,China	1999.06.22
B22000003	B2	Antiqua		Xinjiang Changji,China	2000.09.23
B22002001	B2	Antiqua		Xinjiang Changji,China	2002.07.10
B22002003	B2	Antiqua		Xinjiang Changji,China	2002.07.22
B22002007	B2	Antiqua		Xinjiang Wulumuqi,China	2002.09.12
B22003003	B2	Antiqua		Xinjiang Wulumuqi,China	2003.09.08
B22003004	B2	Antiqua		Xinjiang Changji,China	2003.09.15
B22003005	B2	Antiqua		Xinjiang Wulumuqi,China	2003.09.18
B41975002	B4	Antiqua		Xinjiang Nileke,China	1975.08.20
B41975003	B4	Antiqua		Xinjiang Yining,China	1975
B41976001	B4	Antiqua	0.ANT1	Xinjiang Nileke,China	1976
B41976003	B4	Antiqua	0.ANT1	Xinjiang Nileke,China	1976
B41989002	B4	Antiqua		Xinjiang Nileke,China	1989.09.13
B32001004	B3	Antiqua		Xinjiang Wusu,China	2001.08.01
B21970001	B2	Antiqua		Xinjiang Manasi,China	1970.08.09
B41975001	B4	Antiqua		Xinjiang Nileke,China	1975.07.29
B41976002	B4	Antiqua		Xinjiang Nileke,China	1976.07.24
B41983001	B4	Antiqua		Xinjiang Nileke,China	1983.07.26
B41989001	B4	Antiqua		Xinjiang Nileke,China	1989.09.13
C2004010	C	Antiqua		Qinghai Qumalai,China	2004
D2004001	D	Antiqua		Qinghai Qilian,China	2004
M2004001	M	Antiqua		Qinghai Chengduo,China	2004
B32003002	B3	Antiqua		Xinjiang Wusu,China	2003.06.28
B42003003	B3	Antiqua		Xinjiang Wusu,China	2003.08.07
B32002015	B3	Antiqua		Xinjiang Wusu,China	2002
B31964001	B3	Antiqua		Xinjiang Jinghe,China	1964.07.28
B31970001	B3	Antiqua		Xinjiang Jinghe,China	1970.08.02

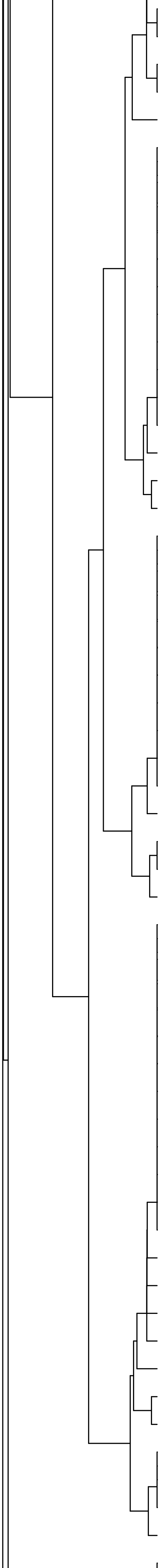

B31964001	B3	Antiqua		Xinjiang Jinghe,China	1964.07.28
B31970001	B3	Antiqua		Xinjiang Jinghe,China	1970.08.02
B30000005	B3	Antiqua		Xinjiang Wusu,China	
B30000006	B3	Antiqua		Xinjiang Wusu,China	
B2005017	B2	Antiqua		Xinjiang Wusu,China	2005
B21955001	B2	Antiqua		Xinjiang Manasi,China	1955.07.21
B21967001	B2	Antiqua		Xinjiang Hutubi,China	1967.08.21
B30000013	B3	Antiqua		Xinjiang Wusu,China	
B31964002	B3	Antiqua		Xinjiang Wusu,China	1964.08.29
B31966001	B3	Antiqua		Xinjiang Jinghe,China	1966
B31971001	B3	Antiqua		Xinjiang Wusu,China	1971.07.31
B31984001	B3	Antiqua		Xinjiang Wusu,China	1984
B31989002	B3	Antiqua		Xinjiang Wusu,China	1989.07.28
B31994002	B3	Antiqua		Xinjiang Wusu,China	1994.07.19
B31997001	B3	Antiqua		Xinjiang Jinghe,China	1997.06.29
B31999001	B3	Antiqua		Xinjiang Jinghe,China	1999.07.12
B31986001	B3	Antiqua		Xinjiang Jinghe,China	1986.06.15
B31980001	B3	Antiqua		Xinjiang Jinghe,China	1980.07.12
B32001002	B3	Antiqua		Xinjiang Wusu,China	2001.07.22
2547	B4	Antiqua		Xingjiang Nileke,China	2005.7.26
2548	B4	Antiqua		Xingjiang Nileke,China	2005.9.20
2652	O	Antiqua		Xinjiang Kelamayi,China	2006.10.10
42084	B3	Antiqua		Xinjiang Wusu,China	
B31968001	B3	Antiqua		Xinjiang Jinghe,China	1968.07.26
B31983001	B3	Antiqua		Xinjiang Wusu,China	1983.05.23
B31983002	B3	Antiqua	0.ANT1	Xinjiang Wusu,China	1983
B32001001	B3	Antiqua		Xinjiang Wusu,China	2001.07.07
K10000003	K1	Antiqua		Xinjiang Hetian,China	
K21987003	K2	Antiqua	0.ANT1	Xinjiang Qiemo,China	1987
42026	B3	Antiqua		Xinjiang Wusu,China	
45113	B3	Antiqua		Xinjiang Jinghe,China	
45115	B3	Antiqua		Xinjiang Jinghe,China	
45114	B3	Antiqua		Xinjiang Jinghe,China	
B21972002	B2	Antiqua	0.ANT1	Xinjiang Hutubi,China	1972
B30000002	B3	Antiqua		Xinjiang Wusu,China	
B30000004	B3	Antiqua		Xinjiang Wusu,China	
B30000010	B3	Antiqua		Xinjiang Wusu,China	
B30000011	B3	Antiqua		Xinjiang Wusu,China	
B30000012	B3	Antiqua		Xinjiang Wusu,China	
B31969001	B3	Antiqua		Xinjiang Jinghe,China	1969.08.01
B31981001	B3	Antiqua	0.ANT1	Xinjiang Jinghe,China	1981
B31982002	B3	Antiqua		Xinjiang Jinghe,China	1982.10.03
B32002007	B3	Antiqua		Xinjiang Wusu,China	2002.08.19
B32002014	B3	Antiqua		Xinjiang Wusu,China	2002.08.25
B32005017	B3	Antiqua		Xinjiang Wusu,China	2005
A1966001	A	Antiqua	0.ANT1	Xinjiang Wuqia,China	1966
B31967001	B3	Antiqua		Xinjiang Wusu,China	1967.08.01
B31984002	B3	Antiqua		Xinjiang Wusu,China	1984.08.21
B31987001	B3	Antiqua		Xinjiang Wusu,China	1987.07.21
B30000003	B3	Antiqua		Xinjiang Wusu,China	
B31965001	B3	Antiqua	0.ANT1	Xinjiang Jinghe,China	1965
B41976004	B4	Antiqua		Xinjiang Nileke,China	1976.08.07
B30000007	B3	Antiqua		Xinjiang Wusu,China	
B30000008	B3	Antiqua		Xinjiang Wusu,China	
B30000009	B3	Antiqua		Xinjiang Wusu,China	
B22001001	B2	Antiqua		Xinjiang Changji,China	2001.08.29
M0000002	M	Microtus	0.PE4	Qinghai Chengduo,China	2001

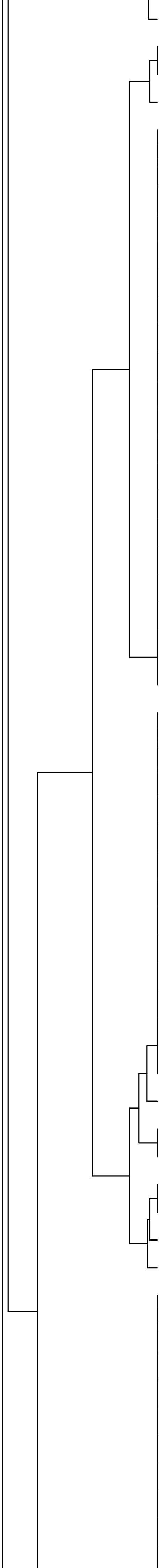

B22001001	B2	Antiqua		Xinjiang Changji,China	2001.08.29
M0000002	M	Microtus	0.PE4	Qinghai Chengduo,China	2001
M2001009	M	Microtus	0.PE4	Qinghai Chengduo,China	2001
M1997001	M	Microtus	0.PE4	Sichuan Shiqu,China	1997
M0000001	M	Antiqua		Qinghai Chengduo,China	
M0000003	M	Antiqua		Qinghai Chengduo,China	
M0000004	M	Antiqua		Qinghai Chengduo,China	
M0000005	M	Microtus		Qinghai Chengduo,China	
M0000006	M	Microtus		Qinghai Chengduo,China	
M0000007	M	Microtus		Qinghai Chengduo,China	
M0000008	M	Microtus		Qinghai Chengduo,China	
M0000009	M	Microtus		Qinghai Chengduo,China	
M0000010	M	Microtus		Qinghai Chengduo,China	
M1997005	M	Microtus		Sichuan Shiqu,China	1997.07.14
M2000003	M	Microtus		Qinghai Chengduo,China	2000.06.21
M2000005	M	Microtus		Sichuan Shiqu,China	2000.06.29
M2000006	M	Microtus		Sichuan Shiqu,China	2000.06.29
M2001001	M	Microtus		Qinghai Chengduo,China	2001.06.21
M2001002	M	Microtus		Qinghai Chengduo,China	2001.06.27
M2001003	M	Microtus		Qinghai Chengduo,China	2001.06.30
M2001004	M	Microtus		Qinghai Chengduo,China	2001.07.11
M2001005	M	Microtus		Qinghai Chengduo,China	2001.07.15
M2001006	M	Microtus		Qinghai Chengduo,China	2001.07.15
M2001008	M	Microtus		Qinghai Chengduo,China	2001.07.15
M2001010	M	Microtus		Qinghai Chengduo,China	2001.07.15
M1997002	M	Microtus		Sichuan Shiqu,China	1997.07.11
M1997003	M	Microtus		Sichuan Shiqu,China	1997.07.11
M1997004	M	Microtus		Sichuan Shiqu,China	1997.07.13
M1997006	M	Microtus		Sichuan Shiqu,China	1997.07.16
M1997009	M	Microtus		Sichuan Shiqu,China	1997.07.07
M1997012	M	Microtus		Sichuan Shiqu,China	1997.07.09
M2000001	M	Microtus		Sichuan Shiqu,China	2000.06.18
M2000002	M	Microtus		Sichuan Shiqu,China	2000.06.20
M2000004	M	Microtus		Sichuan Shiqu,China	2000.06.22
M2001011	M	Microtus		Sichuan Shiqu,China	2001.08.10
M2001012	M	Microtus		Sichuan Shiqu,China	2001.08.17
M2001013	M	Microtus		Sichuan Shiqu,China	2001.08.24
M2002001	M	Microtus		Sichuan Shiqu,China	2002.07.18
M2002002	M	Microtus		Sichuan Shiqu,China	2002.08.20
M2000008	M	Microtus		Sichuan Shiqu,China	2000.07.31
M1997007	M	Microtus		Sichuan Shiqu,China	1997.07.27
M2000007	M	Microtus		Sichuan Shiqu,China	2000.07.19
M1997008	M	Microtus		Sichuan Shiqu,China	1997.07.06
M1997011	M	Microtus		Sichuan Shiqu,China	1997.07.09
M1997010	M	Microtus		Sichuan Shiqu,China	1997.07.09
M2001007	M	Microtus		Qinghai Chengduo,China	2001.07.15
3136		Microtus		inner Mongolia Erlianhaotei,China	2007
3137		Microtus		Inner Mongolia Erlianhaotei,China	2007
L1970001	L	Microtus		Inner Mongolia abagaqi,China	1970.04.21
L1970002	L	Microtus		Inner Mongolia sunitezuoqi,China	1970.05.16
L1970005	L	Microtus		Inner Mongolia abahanaerqi,China	1970.06.03
L1970006	L	Microtus		Inner Mongolia abahanaerqi,China	1970.06.07
L1970007	L	Microtus		Inner Mongolia abahanaerqi,China	1970.06.08
L1970008	L	Microtus		Inner Mongolia abahanaerqi,China	1970.06.16
L1970009	L	Microtus		Inner Mongolia abagaqi,China	1970.06.21
L1970011	L	Microtus		Inner Mongolia abagaqi,China	1970.06.23
[illegible]	[illegible]	[illegible]		[illegible]	[illegible]

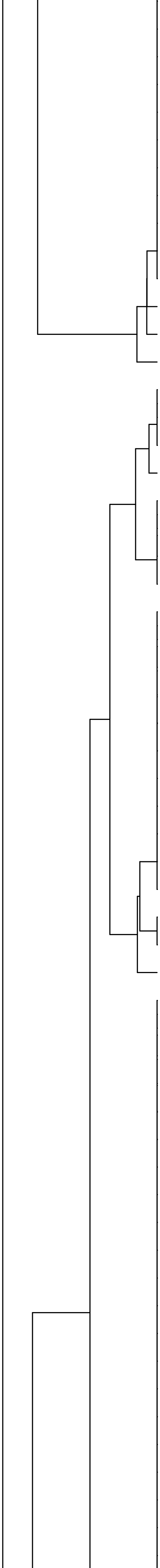

L1970009	L	Microtus		Inner Mongolia abagaqi,China	1970.06.21
L1970011	L	Microtus		Inner Mongolia abagaqi,China	1970.06.23
L1970012	L	Microtus		Inner Mongolia sunitezuoqi,China	1970.06.25
L1970013	L	Microtus		Inner Mongolia sunitezuoqi,China	1970.06.28
L1970014	L	Microtus		Inner Mongolia abagaqi,China	1970.06.28
L1970015	L	Microtus		Inner Mongolia abahanaerqi,China	1970.07.10
L1970017	L	Microtus		Inner Mongolia abahanaerqi,China	1970.07.15
L1976001	L	Microtus		Inner Mongolia Xiwuzhumuqinqi,China	1976.05.24
L1976002	L	Microtus		Inner Mongolia Xiwuzhumuqinqi,China	1976.06.19
L1987001	L	Microtus		Inner Mongolia sunitezuoqi,China	1987.12.05
L1989001	L	Microtus		Inner Mongolia Dongwuzhumuqinqi,China	1989.05.19
L1989002	L	Microtus		Inner Mongolia Dongwuzhumuqinqi,China	1989.05.25
91001		Microtus	0.PE4	China	1970
L1970004	L	Microtus		Inner Mongolia Dongwuzhumuqinqi,China	1970.05.16
C1988002	C	Antiqua		Qinghai Xinghai,China	1988.03.10
C2004005	C	Antiqua		Qinghai Wulan,China	2004
D1990001	D	Antiqua		Qinghai Qilian,China	1990.06.08
C2000001	C	Antiqua	1.IN2	Qinghai Delingha,China	2000
C1988001	C	Antiqua		Qinghai Xinghai,China	1988.03.08
C1993002	C	Antiqua		Gansu Yumen,China	1993
D0000002	D	Antiqua		Qinghai Qilian,China	
D0000003	D	Antiqua		Qinghai Qilian,China	
C1970005	C	Antiqua	1.IN2	Qinghai Gonghe,China	1970
C1977001	C	Antiqua		Gansu Yumen,China	1977.09.30
C1979002	C	Antiqua	1.IN2	Qinghai Mangya,China	1979
C1983006	C	Antiqua		Qinghai Haiyan,China	1983.08.26
C1985001	C	Antiqua		Qinghai Gonghe,China	1985.07.06
C1996001	C	Antiqua		Qinghai Delingha,China	1996.07.24
C2000002	C	Antiqua		Qinghai Tianjun,China	2000.09.11
C2002001	C	Antiqua		Qinghai Delingha,China	2002.08.22
C2003002	C	Antiqua		Qinghai Wulan,China	2003.08.16
D1963001	D	Antiqua		Qinghai Qilian,China	1963.06.02
D2001003	D	Antiqua		Qinghai Tongde,China	2001.06.04
C1961007	C	Antiqua		Qinghai Xinghai,China	1961.6.11
F1952001	F	Antiqua	1.IN2	Yunnan Midu,China	1952
C1978001	C	Antiqua		Qinghai Maduo,China	1978.07.24
C0000002	C	Antiqua		Qinghai Gonghe,China	
C1960002	C	Antiqua		Qinghai Haiyan,China	1960.06.25
C1961002	C	Antiqua		Qinghai Gonghe,China	1961.06.02
C1961003	C	Antiqua		Qinghai Gonghe,China	1961.06.15
C1961004	C	Antiqua		Gansu Sunan,China	1961.06.28
C1964002	C	Antiqua		Qinghai Dulan,China	1964.08.11
C1964003	C	Antiqua		Qinghai Haiyan,China	1964.08.15
C1966001	C	Antiqua		Qinghai Haiyan,China	1966.08.12
C1966002	C	Antiqua		Qinghai Tianjun,China	1966.09.01
C1967002	C	Antiqua		Qinghai Gonghe,China	1967.06.19
C1968001	C	Antiqua		Qinghai Gangcha,China	1968.05.30
C1969002	C	Antiqua		Qinghai Wulan,China	1969.08.25
C1970002	C	Antiqua		Qinghai Xinghai,China	1970.06.17
C1970006	C	Antiqua		Qinghai Gonghe,China	1970.07.29
C1970007	C	Antiqua		Qinghai Guide,China	1970.08.09
C1970008	C	Antiqua		Qinghai Dulan,China	1970.09.15
C1971004	C	Antiqua		Gansu Subei,China	1971.08.05
C1972004	C	Antiqua		Gansu Yumen,China	1972.07.26
C1973001	C	Antiqua		Qinghai Gangcha,China	1973.08.17
C1974001	C	Antiqua		Qinghai Gangcha,China	1974.07.13
C1974002	C	Antiqua		Qinghai Tianjun,China	1974.08.01

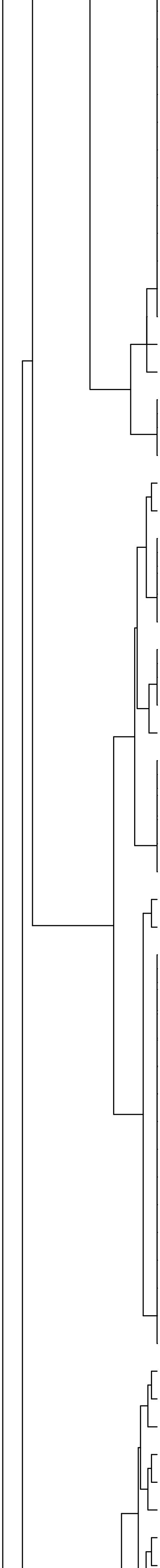

C1974001	C	Antiqua		Qinghai Gangcha,China	1974.07.13
C1974002	C	Antiqua		Qinghai Tianjun,China	1974.08.01
C1974003	C	Antiqua		Qinghai Gangcha,China	1974.08.24
C1975001	C	Antiqua		Qinghai Zhiduo,China	1975.08.01
C1982001	C	Antiqua		Gansu Subei,China	1982.07.17
C1983007	C	Antiqua		Gansu Subei,China	1983.09.13
D0000001	D	Antiqua		Qinghai Qilian,China	
D1963002	D	Antiqua		Qinghai Menyuan,China	1963.07.02
D1963003	D	Antiqua		Qinghai Menyuan,China	1963.07.23
D1975001	D	Antiqua		Gansu Sunan,China	1975.06.
D2001001	D	Antiqua		Qinghai Tongde,China	2001.05.27
D2001002	D	Antiqua		Qinghai Tongde,China	2001.06.04
C1971003	C	Antiqua		Qinghai Huangyuan,China	1971.06.19
C1967003	C	Antiqua		Qinghai Gonghe,China	1967.09.20
C1964006	C	Antiqua		Qinghai Haiyan,China	1964.10.10
C1970001	C	Antiqua		Qinghai Guide,China	1970.06.14
C1971001	C	Antiqua		Qinghai Huangyuan,China	1971.05.14
E1977003	E	Antiqua		Yunnan Jianchuan,China	1977
F1954005	F	Orientalis		Yunnan Xiangyun,China	1954
E1954003	E	Orientalis		Yunnan Jianchuan,China	1954
E1957003	E	Antiqua		Yunnan Jianchuan,China	1957
E1983001	E	Antiqua		Yunnan Jianchuan,China	1983
E1985001	E	Antiqua		Yunnan Jianchuan,China	1985
E1982001	E	Antiqua		Yunnan Jianchuan,China	1982.12.23
E1984003	E	Antiqua		Yunnan Jianchuan,China	1984.04.27
F1984005	F	Antiqua		Yunnan Longchuan,China	1984
E1957004	E	Antiqua		Yunnan Jianchuan,China	1957
E1977002	E	Antiqua		Yunnan Jianchuan,China	1977.07.31
E1979003	E	Antiqua		Yunnan Jianchuan,China	1979.12.31
E1982002	E	Antiqua		Yunnan Jianchuan,China	1982.12.28
E1983004	E	Antiqua		Yunnan Jianchuan,China	1983.10.03
E1984002	E	Antiqua		Yunnan Jianchuan,China	1984.04.27
C1985002	C	Antiqua		Qinghai Guinan,China	1985.07.29
E1979001	E	Antiqua	1.IN3	China	1979
E1954001	E	Antiqua	1.IN3	Yunnan Jianchuan,China	1954
E1955001	E	Antiqua		Yunnan Jianchuan,China	1955.06.12
E1957001	E	Antiqua		Yunnan Jianchuan,China	1957
E1957002	E	Antiqua		Yunnan Jianchuan,China	1957
E1977001	E	Antiqua	1.IN3	Yunnan Jianchuan,China	1977
E1977004	E	Antiqua		Yunnan Jianchuan,China	1977.08.03
E1979004	E	Antiqua		Yunnan Jianchuan,China	1979
E1980001	E	Antiqua		Yunnan Jianchuan,China	1980
E1984004	E	Antiqua		Yunnan Jianchuan,China	1984
E1984006	E	Antiqua		Yunnan Jianchuan,China	1984.08.13
E1990001	E	Antiqua		Yunnan Jianchuan,China	1990
F1954001	F	Antiqua		Yunnan Xiangyun,China	1954.11.08
F1954003	F	Antiqua		Yunnan Xiangyun,China	1954.12.09
F1990004	F	Antiqua		Yunnan Yingjiang,China	1990
F2000001	F	Antiqua		Yunnan Yingjiang,China	2000
180001	C	Antiqua		Qinghai Luhuo,China	1990.5.28
H1954003	H	Antiqua	1.IN2	Inner Mongolia Baochang,China	1954
D1982001	D	Antiqua	1.IN2	Gansu Sunan,China	1982
C1959001	C	Antiqua		Gansu Xiahe,China	1959.08.01
C1994002	C	Antiqua	1.IN2	Tibet Biru,China	1994
C1993001	C	Antiqua		Qinghai Chengduo,China	1993.08.05
C1964005	C	Antiqua		Qinghai Gonghe,China	1964.09.12
C1990001	C	Antiqua		Qinghai Chengduo,China	1990.07.05

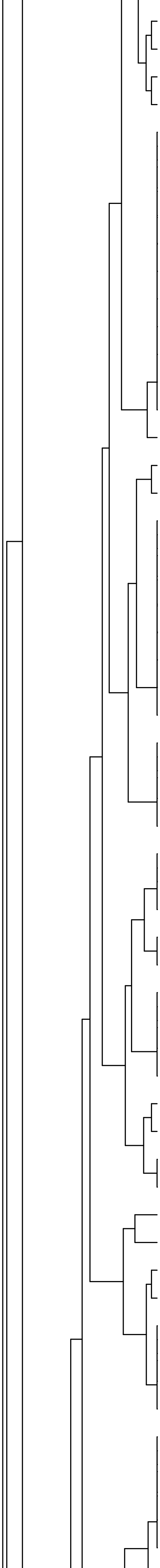

[illegible]	[illegible]	[illegible]		[illegible]	
C1964005	C	Antiqua		Qinghai Gonghe,China	1964.09.12
C1990001	C	Antiqua		Qinghai Chengduo,China	1990.07.05
C1962003	C	Antiqua	1.IN2	Qinghai Xinghai,China	1962
D1991005	D	Antiqua	1.IN2	Qinghai Zeku,China	1991
C1954001	C	Antiqua	1.IN2	Qinghai Guinan,China	1954
C1960001	C	Antiqua	1.IN2	Qinghai Tongren,China	1960
C1969001	C	Antiqua		Qinghai Zhaduo,China	1969.07.23
C1973002	C	Antiqua		Qinghai Nangqian,China	1973.09.09
C1974004	C	Antiqua		Qinghai Zhaduo,China	1974.09.06
C1974005	C	Antiqua		Qinghai Zhaduo,China	1974.09.23
C1983008	C	Antiqua		Qinghai Maduo,China	1983.11.06
C1986001	C	Antiqua		Qinghai Tongren,China	1986.06.24
C1986002	C	Antiqua		Qinghai Tongren,China	1986.07.26
C1986003	C	Antiqua		Tibe Chaya,China	1986.08.21
C1994003	C	Antiqua		Qinghai Dulan,China	1994.09.24
C1980004	C	Antiqua		Qinghai Maduo,China	1980.11.07
C1985003	C	Antiqua		Qinghai Guinan,China	1985.07.31
D1985002	D	Antiqua		Qinghai Qilian,China	1985.10.07
C0000001	C	Antiqua		Qinghai Zhunhua,China	
C1960003	C	Antiqua		Gansu Xiahe,China	1960.09.10
C1960004	C	Antiqua		Gansu Xiahe,China	1960.10.05
C1964004	C	Antiqua		Gansu Sunan,China	1964.08.20
C1970009	C	Antiqua		Qinghai Zhunhua,China	1970.09.23
C1979003	C	Antiqua		Qinghai Maqin,China	1979.08.03
C1980001	C	Antiqua		Qinghai Maqin,China	1980.10.18
C1983004	C	Antiqua		Tibe Chaya,China	1983.08.11
C1983001	C	Antiqua		Tibet Changdu,China	1983.07.03
C1983002	C	Antiqua		Tibet Changdu,China	1983.07.04
C1998001	C	Antiqua		Tibet Changdu,China	1998.05.29
C1998002	C	Antiqua		Tibet Dangxiong,China	1998.07.28
D1991002	D	Antiqua		Qinghai Zeku,China	1991.09.05
D1991003	D	Antiqua		Qinghai Tongde,China	1991.09.05
D1991006	D	Antiqua		Qinghai Zeku,China	1991.09.10
C1978009	C	Antiqua		Tibet Dingqing,China	1978.09.07
C1990002	C	Antiqua		Tibe Baqing,China	1990.08.04
C1985004	C	Antiqua		Qinghai Guinan,China	1985.08.19
C1997001	C	Antiqua		Qinghai Nangqian,China	1997.11.15
D1991001	D	Antiqua		Qinghai Tongde,China	1991.09.05
D1991004	D	Antiqua	1.IN2	Qinghai Zeku,China	1991
C1975002	C	Antiqua		Qinghai Yushu,China	1975.09.01
C1975003	C	Antiqua	1.IN2	Qinghai Yushu,China	1975
C1964001	C	Antiqua		Qinghai Yushu,China	1964.07.11
C1964007	C	Antiqua		Qinghai Yushu,China	1964.10.17
C1967001	C	Antiqua		Qinghai Wulan,China	1967
D1984001	D	Antiqua	1.IN2	Gansu Sunan,China	1984
C1960005	C	Antiqua		Gansu Xiahe,China	1960.11.05
C1978011	C	Antiqua		Qinghai Nangqian,China	1978.10.27
C1978003	C	Antiqua		Qinghai Yushu,China	1978.07.28
C1978010	C	Antiqua		Qinghai Nangqian,China	1978.10.27
C1982002	C	Antiqua		Qinghai Yushu,China	1982.07.28
C1990004	C	Antiqua		Qinghai Nangqian,China	1990.08.16
C1954003	C	Antiqua		Qinghai Heinan,China	1954
D1960001	D	Antiqua		Qinghai Qilian,China	1960.05.26
D1964001	D	Antiqua	1.IN2	Qinghai Menyuan,China	1964
D1964002	D	Antiqua	1.IN2	Qinghai Menyuan,China	1964
D1983001	D	Antiqua		Qinghai Menyuan,China	1983.04.04
[illegible]	[illegible]	[illegible]		[illegible]	[illegible]

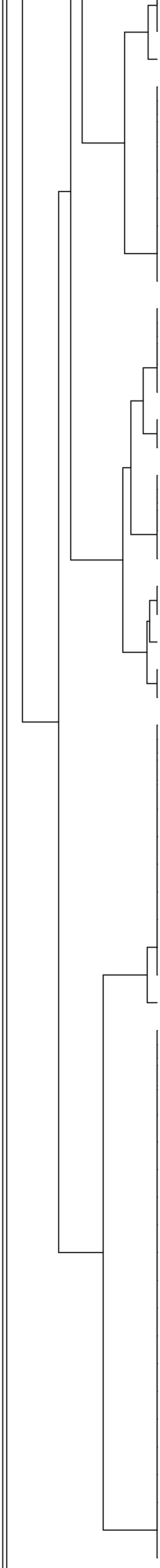

D1964002	D	Antiqua	1.IN2	Qinghai Menyuan,China	1964
D1983001	D	Antiqua		Qinghai Menyuan,China	1983.04.04
D1967001	D	Antiqua		Qinghai Menyuan,China	1967.08.23
C1954002	C	Antiqua		Qinghai Heinan,China	1954
C1961005	C	Antiqua		Gansu Sunan,China	1961.07.16
C1983003	C	Antiqua		Gansu Akesai,China	1983.07.11
D0000004	D	Antiqua		Qinghai Menyuan,China	
D1958001	D	Antiqua		Qinghai Qilian,China	1958
D1965001	D	Antiqua		Qinghai Qilian,China	1965.09.21
D1977001	D	Antiqua		Qinghai Qilian,China	1977.09.21
D1985001	D	Antiqua		Gansu Sunan,China	1985.06.08
2650	O	Antiqua		Xinjiang Kelamayi,China	2006.10.9
2651	O	Antiqua		Xingjiang Mosuowan,China	2006.10.9
2656	O	Antiqua		Xinjiang Qitai,China	2006.10.06
K22005001	K2	Antiqua		Xinjiang Ruoqiang,China	2005.10.9
2638	K2	Antiqua		Xinjiang Qiemo,China	2006.08.09
2640	K2	Antiqua		Xinjiang Qiemo,China	2006.08.09
K21985002	K2	Antiqua	1.IN1	Xinjiang Ruoqiang,China	1985
K21985004	K2	Antiqua		Xinjiang Ruoqiang,China	1985.07.25
K21985006	K2	Antiqua	1.IN1	Xinjiang Ruoqiang,China	1985
K21985007	K2	Antiqua		Xinjiang Ruoqiang,China	1985.08.11
C1979001	C	Antiqua		Qinghai Mangya,China	1979.07.19
K21985003	K2	Antiqua		Xinjiang Ruoqiang,China	1985.07.21
K21985008	K2	Antiqua		Xinjiang Ruoqiang,China	1985.08.27
C1978007	C	Antiqua		Qinghai Zhiduo,China	1978.08.25
C1978008	C	Antiqua		Qinghai Zhiduo,China	1978.08.25
C1976002	C	Antiqua	1.IN2	Tibet Naqu,China	1976
C1976005	C	Antiqua		Tibet Naqu,China	1976.07.14
C1976006	C	Antiqua		Tibet Naqu,China	1976.07.31
C1980002	C	Antiqua	1.IN2	Qinghai Qumalai,China	1980
C1987001	C	Antiqua		Tibe Aanduo,China	1987.07.21
C1988003	C	Antiqua		Tibe Aanduo,China	1988.06.10
C1988004	C	Antiqua		Tibe Nierong,China	1988.09.13
C1989001	C	Antiqua	1.IN2	Qinghai Zhaduo,China	1989
C1992003	C	Antiqua		Qinghai Zhiduo,China	1992.11.03
C2003001	C	Antiqua		Qinghai Geermu,China	2003.06.27
C1998003	C	Antiqua		Qinghai Qumalai,China	1998.09.15
C1962001	C	Antiqua		Qinghai Huangyuan,China	1962.08.13
C1965001	C	Antiqua		Qinghai Yushu,China	1965.06.03
C1970003	C	Antiqua		Qinghai Qumalai,China	1970.06.30
C1970004	C	Antiqua		Qinghai Qumalai,China	1970.06.30
C1971002	C	Antiqua		Qinghai Guide,China	1971.06.04
C1976003	C	Antiqua		Tibet Naqu,China	1976.06.24
C1976004	C	Antiqua		Tibet Naqu,China	1976.07.12
C1976007	C	Antiqua		Tibet Naqu,China	1976.08.07
C1978002	C	Antiqua		Tibet Naqu,China	1978.07.27
C1978004	C	Antiqua		Tibet Naqu,China	1978.08.04
C1978005	C	Antiqua		Tibet Naqu,China	1978.08.04
C1978006	C	Antiqua		Tibet Naqu,China	1978.08.18
C1980003	C	Antiqua		Tibet Biru,China	1980.11.03
C1983005	C	Antiqua		Qinghai Zhiduo,China	1983.08.14
C1987002	C	Antiqua		Tibe Aanduo,China	1987.08.11
C1990003	C	Antiqua		Tibe Aanduo,China	1990.08.04
C1991001	C	Antiqua		Qinghai Geermu,China	1991.08.24
C1992001	C	Antiqua		Tibe Nierong,China	1992.07.14
C1992002	C	Antiqua		Tibe Aanduo,China	1992.08.05
C1994004	C	Antiqua		Tibet Dangxiong,China	1994.10.23

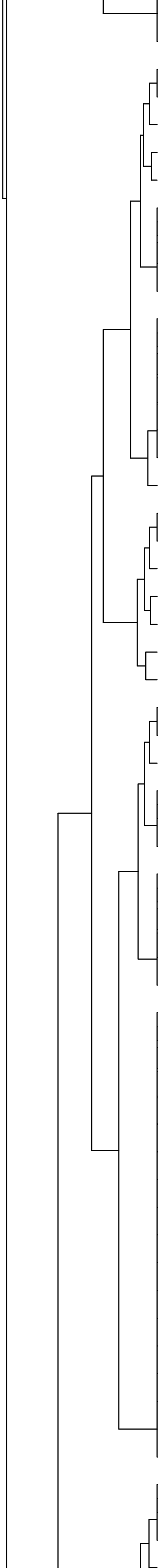

C1992002	C	Antiqua		Tibe Aanduo,China	1992.08.05
C1994004	C	Antiqua		Tibet Dangxiong,China	1994.10.23
F1987001	F	Orientalis		Yunnan Wanting,China	1987
F1996018	F	Orientalis		Yunnan Jianshui,China	1996
F1999009	F	Orientalis		Yunnan Honghe,China	1999
F1950001	F	Orientalis		Fujian Huaan,China	1950
F1995006	F	Orientalis		Yunnan Jinggu,China	1995
F1990011	F	Orientalis		Yunnan Lianghe,China	1990
F2001002	F	Orientalis		Yunnan Pingbian,China	2001
F1994006	F	Orientalis		Yunnan Shidian,China	1994
F1996014	F	Orientalis		Yunnan Lianghe,China	1996
F1982002	F	Orientalis		Yunnan Longchuan,China	1982.07.28
F1982003	F	Orientalis		Yunnan Ruili,China	1982.08.13
F1983006	F	Orientalis		Yunnan Baoshan,China	1983.09.06
F1990013	F	Orientalis		Yunnan Yingjiang,China	1990.09.18
F1990015	F	Orientalis		Yunnan Lianghe,China	1990.11.03
F1991002	F	Orientalis		Yunnan Wanting,China	1991.06.07
F1943002	F	Orientalis		Fujian Fuzhou,China	1943
F1991016	F	Orientalis	1.ORI2	China	1991
India195		Orientalis	1.ORI1	India	>1898
CA88-4125		Orientalis	1.ORI1	USA	1988
YN663	F	Orientalis	1.ORI2	Yunnan Longchuan,China	1982
FV-I		Orientalis	1.ORI1	USA	2001
CO92		Orientalis	1.ORI1	USA	1992
MG05-1020		Orientalis	1.ORI3	Madagascar	2005
F1991017	F	Orientalis		Yunnan Gengma,China	1991
F1997001	F	Orientalis		Yunnan Kaiyuan,China	1997
F1999004	F	Orientalis		Yunnan Luoxi,China	1999
F0000004	F	Orientalis		Yunnan Mengzi,China	
F1983012	F	Orientalis		Yunnan Ruili,China	1983
F1996009	F	Orientalis		Yunnan Mojiang,China	1996
F1990010	F	Orientalis		Yunnan Longchuan,China	1990.08.04
F1992001	F	Orientalis		Yunnan Lanchang,China	1992.01.22
F1992010	F	Orientalis		Yunnan Shuangjiang,China	1992
F1996021	F	Orientalis		Yunnan Gejiu,China	1996
F1999006	F	Orientalis		Yunnan Yunxian,China	1999
F1990001	F	Orientalis		Yunnan Ruili,China	1990
F2002003	F	Orientalis		Yunnan Yanshan,China	2002
F1990022	F	Orientalis		Yunnan Gengma,China	1990
F1990023	F	Orientalis		Yunnan Gengma,China	1990
F1991005	F	Orientalis		Yunnan Wanting,China	1991
F1991006	F	Orientalis		Yunnan Zhenkang,China	1991.07.08
F1991015	F	Orientalis		Yunnan Changyuan,China	1991
F1992012	F	Orientalis		Yunnan Changyuan,China	1992.11.12
F1994003	F	Orientalis		Yunnan Tengchong,China	1994
F1994007	F	Orientalis		Yunnan Shidian,China	1994
F1995002	F	Orientalis		Yunnan Puer,China	1995
F1995005	F	Orientalis		Yunnan Zhenkang,China	1995
F1995008	F	Orientalis		Myanmar	1995
F1995009	F	Orientalis		Yunnan Yingjiang,China	1995
F1996001	F	Orientalis		Yunnan Mojiang,China	1996.04.03
F1996007	F	Orientalis		Yunnan Mojiang,China	1996.08.26
F1996019	F	Orientalis		Yunnan Gejiu,China	1996
F1943001	F	Orientalis		Fujian Fuzhou,China	1943
F1952004	F	Orientalis		Fujian Putian,China	1952
F2002002	F	Orientalis		Yunnan Wenshan,China	2002
F1992002	F	Orientalis		Yunnan Linchang,China	1992

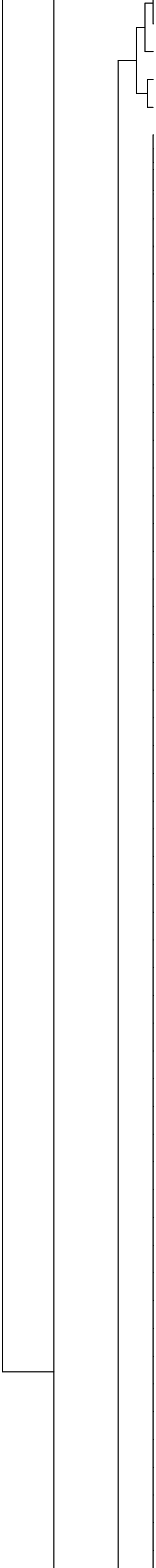

F2002002	F	Orientalis		Yunnan Wenshan,China	2002
F1992002	F	Orientalis		Yunnan Linchang,China	1992
F1946001	F	Orientalis	1.ORI2	Fujian Gutian,China	1946
F1986001	F	Orientalis		Yunnan Yingjiang,China	1986
E1983002	E	Orientalis		Yunnan Jianchuan,China	1983
E1983003	E	Orientalis		Yunnan Jianchuan,China	1983
F0000001	F	Orientalis		Yunnan Yanshan,China	
F0000002	F	Orientalis		Guangxi Longlin,China	
F1954002	F	Orientalis		Yunnan Xiangyun,China	1954
F1958001	F	Orientalis		Yunnan Dali,China	1958
F1964001	F	Orientalis		Yunnan Lianghe,China	1964
F1982001	F	Orientalis		Yunnan Longchuan,China	1982.07.28
F1982004	F	Orientalis		Yunnan Ruili,China	1982.08.15
F1982005	F	Orientalis		Yunnan Ruili,China	1982.08.30
F1982006	F	Orientalis		Yunnan Longchuan,China	1982
F1982007	F	Orientalis		Yunnan Ruili,China	1982
F1983001	F	Orientalis		Yunnan Ruili,China	1983
F1983002	F	Orientalis		Yunnan Ruili,China	1983.08.11
F1983003	F	Orientalis		Yunnan Baoshan,China	1983.08.24
F1983004	F	Orientalis		Yunnan Ruili,China	1983
F1983005	F	Orientalis		Yunnan Baoshan,China	1983.09.06
F1983007	F	Orientalis		Yunnan Baoshan,China	1983.09.07
F1983008	F	Orientalis		Yunnan Longyang,China	1983
F1983009	F	Orientalis		Yunnan Longyang,China	1983
F1983010	F	Orientalis		Yunnan Longyang,China	1983
F1983011	F	Orientalis		Yunnan Ruili,China	1983
F1984001	F	Orientalis	1.ORI2	Yunnan Yingjiang,China	1984
F1984004	F	Orientalis		Yunnan Yingjiang,China	1984
F1986003	F	Orientalis		Yunnan Yingjiang,China	1986
F1990002	F	Orientalis		Yunnan Ruili,China	1990
F1990005	F	Orientalis		Yunnan Yingjiang,China	1990
F1990006	F	Orientalis		Yunnan Yingjiang,China	1990
F1990007	F	Orientalis		Yunnan Yingjiang,China	1990
F1990008	F	Orientalis		Yunnan Dali,China	1990
F1990009	F	Orientalis		Yunnan Dali,China	1990
F1990012	F	Orientalis		Yunnan Lianghe,China	1990
F1990014	F	Orientalis		Yunnan Yingjiang,China	1990.10.05
F1990016	F	Orientalis		Yunnan Longchuan,China	1990.11.08
F1990017	F	Orientalis		Yunnan Lianghe,China	1990.11.15
F1990018	F	Orientalis		Yunnan Lianghe,China	1990.11.19
F1990019	F	Orientalis		Yunnan Lianghe,China	1990.11.19
F1990021	F	Orientalis		Yunnan Gengma,China	1990
F1990025	F	Orientalis		Yunnan Gengma,China	1990
F1991001	F	Orientalis		Yunnan Luoxi,China	1991
F1991003	F	Orientalis		Yunnan Wanting,China	1991.06.10
F1991004	F	Orientalis		Yunnan Wanting,China	1991.06.12
F1991007	F	Orientalis		Myanmar	1991
F1991008	F	Orientalis		Yunnan Zhenkang,China	1991
F1991009	F	Orientalis		Myanmar	1991
F1991010	F	Orientalis		Yunnan Yingjiang,China	1991
F1991011	F	Orientalis		Yunnan Gengma,China	1991.08.18
F1991012	F	Orientalis		Yunnan Gengma,China	1991
F1991013	F	Orientalis		Yunnan Longchuan,China	1991
F1991014	F	Orientalis		Yunnan Changyuan,China	1991
F1991018	F	Orientalis		Yunnan Gengma,China	1991
F1992003	F	Orientalis		Yunnan Linchang,China	1992

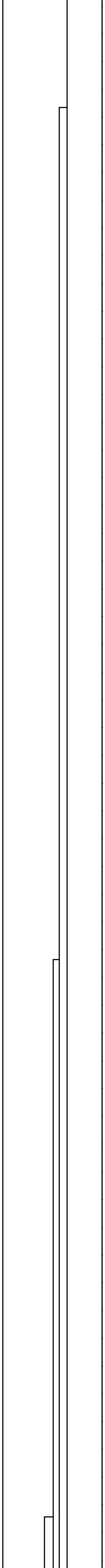

F1991018	F	Orientalis		Yunnan Gengma,China	1991
F1992003	F	Orientalis		Yunnan Linchang,China	1992
F1992004	F	Orientalis		Yunnan Lanchang,China	1992.02.20
F1992005	F	Orientalis		Yunnan Menghai,China	1992
F1992008	F	Orientalis		Yunnan Yuanjiang,China	1992
F1992009	F	Orientalis		Yunnan Shuangjiang,China	1992
F1992014	F	Orientalis		Yunnan Changyuan,China	1992.11.26
F1993001	F	Orientalis		Yunnan Lanchang,China	1993.01.31
F1993002	F	Orientalis		Yunnan Jinghong,China	1993
F1993003	F	Orientalis		Yunnan Jinghong,China	1993
F1994002	F	Orientalis		Yunnan Tengchong,China	1994
F1994004	F	Orientalis		Yunnan Shidian,China	1994
F1994005	F	Orientalis		Yunnan Shidian,China	1994
F1995001	F	Orientalis		Yunnan Puer,China	1995
F1995004	F	Orientalis		Yunnan Lanchang,China	1995
F1995007	F	Orientalis		Myanmar	1995
F1996003	F	Orientalis		Yunnan Yunxian,China	1996
F1996004	F	Orientalis		Yunnan Gengma,China	1996.05.11
F1996005	F	Orientalis		Yunnan Gengma,China	1996.05.13
F1996006	F	Orientalis		Yunnan Gengma,China	1996
F1996008	F	Orientalis		Yunnan Mojiang,China	1996
F1996010	F	Orientalis		Yunnan Mojiang,China	1996
F1996011	F	Orientalis		Yunnan Mojiang,China	1996
F1996015	F	Orientalis		Yunnan Lianghe,China	1996
F1996016	F	Orientalis		Yunnan Jianshui,China	1996
F1996017	F	Orientalis		Yunnan Jianshui,China	1996
F1996020	F	Orientalis		Yunnan Gejiu,China	1996
F1996022	F	Orientalis		Yunnan Wenshan,China	1996
F1996023	F	Orientalis		Yunnan Yanshan,China	1996
F1996024	F	Orientalis		Yunnan Shiping,China	1996
F1996025	F	Orientalis		Yunnan Shiping,China	1996
F1996026	F	Orientalis		Yunnan Shiping,China	1996
F1996027	F	Orientalis		Yunnan Shiping,China	1996
F1997002	F	Orientalis		Yunnan Yanshan,China	1997
F1997003	F	Orientalis		Yunnan Yiliang,China	1997
F1997004	F	Orientalis		Yunnan Fumin,China	1997
F1997005	F	Orientalis		Yunnan Fumin,China	1997
F1999001	F	Orientalis		Yunnan Jinghong,China	1999
F1999002	F	Orientalis		Yunnan Mengla,China	1999
F1999003	F	Orientalis		Yunnan Mengla,China	1999
F1999007	F	Orientalis		Yunnan Zhenyuan,China	1999
F1999011	F	Orientalis		Yunnan Zhenyuan,China	1999
F2000002	F	Orientalis		Guizhou Yixing,China	2000.08.01
F2000003	F	Orientalis		Guizhou Yixing,China	2000.08.07
F2000004	F	Orientalis	1.ORI2	Guizhou Yixing,China	2000
F2000005	F	Orientalis		Guizhou Yixing,China	2000.08.20
F2000006	F	Orientalis		Guangxi Longlin,China	2000
F2000007	F	Orientalis		Guangxi Longlin,China	2000
F2000008	F	Orientalis		Guangxi Longlin,China	2000
F2000009	F	Orientalis		Guangxi Longlin,China	2000
F2000010	F	Orientalis		Guangxi Longlin,China	2000
F2000011	F	Orientalis		Guangxi Longlin,China	2000
F2001001	F	Orientalis		Yunnan Simao,China	2001
F2001005	F	Orientalis		Yunnan Yanshan,China	2001
F2001006	F	Orientalis		Yunnan Linchang,China	2001
F2001008	F	Orientalis		Yunnan Fengqing,China	2001
F2002001	F	Orientalis		Yunnan Honghe,China	2002

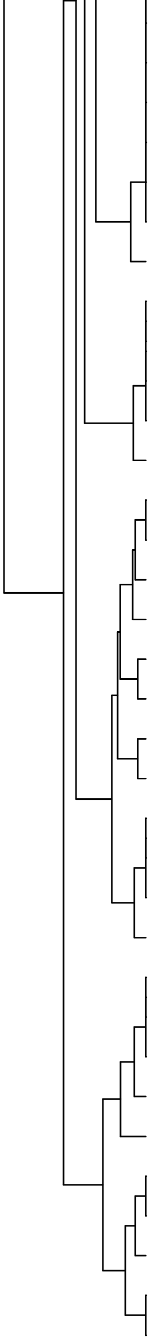

F2001008	F	Orientalis		Yunnan Fengqing,China	2001
F2002001	F	Orientalis		Yunnan Honghe,China	2002
F2002004	F	Orientalis		Yunnan Mile,China	2002
F2002005	F	Orientalis		Yunnan Mile,China	2002
F2002006	F	Orientalis		Guizhou Yixing,China	2002.10.08
F2002007	F	Orientalis		Guizhou Yixing,China	2002.10.12
F1996002	F	Orientalis		Yunnan Mojiang,China	1996.04.03
F1992013	F	Orientalis		Yunnan Changyuan,China	1992.11.16
F1996012	F	Orientalis		Yunnan Menglian,China	1996
F1999010	F	Orientalis		Yunnan Zhenyuan,China	1999
F2001004	F	Orientalis		Yunnan Mengzi,China	2001
F1996013	F	Orientalis		Yunnan Menglian,China	1996
F1992011	F	Orientalis		Yunnan Shuangjiang,China	1992
F1994001	F	Orientalis		Yunnan Tengchong,China	1994
F2001003	F	Orientalis		Yunnan Pingbian,China	2001
F1986002	F	Orientalis		Yunnan Yingjiang,China	1986
F0000003	F	Orientalis		Yunnan Lanchang,China	
F1985001	F	Orientalis		Yunnan Ruili,China	1985
F1999008	F	Orientalis		Yunnan Yunxian,China	1999
F2001007	F	Orientalis		Yunnan Yunxian,China	2001
F1990003	F	Orientalis		Yunnan Ruili,China	1990
F1990024	F	Orientalis		Yunnan Gengma,China	1990
F1995003	F	Orientalis		Yunnan Jiangcheng,China	1995
F1952003	F	Orientalis		Yunnan Midu,China	1952
F1992006	F	Orientalis		Yunnan Menghai,China	1992
F1999005	F	Orientalis		Yunnan Lianghe,China	1999
F1999012	F	Orientalis		Yunnan Luoxi,China	1999
F1992007	F	Orientalis		Yunnan Yuanjiang,China	1992
IP275		Orientalis	1.ORI3	Madagascar	1995
F1952002	F	Orientalis		Yunnan Midu,China	1952.11.27
F1954004	F	Orientalis		Yunnan Xiangyun,China	1954
F1953003	F	Orientalis	1.ORI2	Yunnan Midu,China	1953
F1953002	F	Orientalis		Yunnan Baoshan,China	1953
F1955001	F	Orientalis		Yunnan Xiangyun,China	1955.02.18
