# Supplementary material for: Features of Variable Number of Tandem Repeats in Yersinia pestis and the Development of a Hierarchical Genotyping Scheme
Source: PLoS One. 2013 Jun 21;8(6):e66567. doi: 10.1371/journal.pone.0066567 (PMC3689786; doi:10.1371/journal.pone.0066567)
Supplement: Figure S7 — Dendrogram of Y. pestis strains clustered with 0.ANT population based on 14+3 VNTR loci. A total of 186 strains were analyzed according to the profiles of 14 primary VNTRs and the loci M23, N2577, and M43 (PDF) [file pone.0066567.s007.pdf]

Categorical
186 0.ANT strains with 17 loci

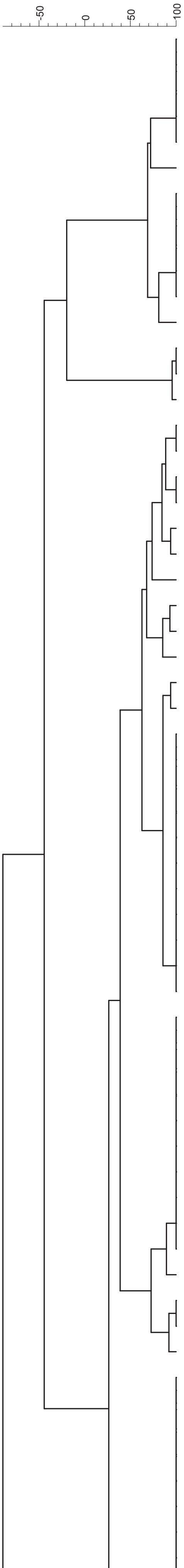

Strain ID	Focus	Biovar	Sub-pop	Location	Time
B21970001	B2	Antiqua		Xinjiang Manasi,China	1970.08.09
B41975001	B4	Antiqua		Xinjiang Nileke,China	1975.07.29
B41976002	B4	Antiqua		Xinjiang Nileke,China	1976.07.24
B41983001	B4	Antiqua		Xinjiang Nileke,China	1983.07.26
B41989001	B4	Antiqua		Xinjiang Nileke,China	1989.09.13
B41976004	B4	Antiqua		Xinjiang Nileke,China	1976.08.07
B41975002	B4	Antiqua		Xinjiang Nileke,China	1975.08.20
B41975003	B4	Antiqua		Xinjiang Yining,China	1975
B41976001	B4	Antiqua	0.ANT1	Xinjiang Nileke,China	1976
B41976003	B4	Antiqua	0.ANT1	Xinjiang Nileke,China	1976
B41989002	B4	Antiqua		Xinjiang Nileke,China	1989.09.13
B32001004	B3	Antiqua		Xinjiang Wusu,China	2001.08.01
C2004010	C	Antiqua		Qinghai Qumalai,China	2004
D2004001	D	Antiqua		Qinghai Qilian,China	2004
M2004001	M	Antiqua		Qinghai Chengduo,China	2004
B32003002	B3	Antiqua		Xinjiang Wusu,China	2003.06.28
B42003003	B3	Antiqua		Xinjiang Wusu,China	2003.08.07
B30000005	B3	Antiqua		Xinjiang Wusu,China	
B30000006	B3	Antiqua		Xinjiang Wusu,China	
B31964001	B3	Antiqua		Xinjiang Jinghe,China	1964.07.28
B31970001	B3	Antiqua		Xinjiang Jinghe,China	1970.08.02
B2005017	B2	Antiqua		Xinjiang Wusu,China	2005
B32002007	B3	Antiqua		Xinjiang Wusu,China	2002.08.19
B32002015	B3	Antiqua		Xinjiang Wusu,China	2002
B31986001	B3	Antiqua		Xinjiang Jinghe,China	1986.06.15
B31980001	B3	Antiqua		Xinjiang Jinghe,China	1980.07.12
B32001002	B3	Antiqua		Xinjiang Wusu,China	2001.07.22
B21955001	B2	Antiqua		Xinjiang Manasi,China	1955.07.21
B21967001	B2	Antiqua		Xinjiang Hutubi,China	1967.08.21
B30000013	B3	Antiqua		Xinjiang Wusu,China	
B31964002	B3	Antiqua		Xinjiang Wusu,China	1964.08.29
B31966001	B3	Antiqua		Xinjiang Jinghe,China	1966
B31971001	B3	Antiqua		Xinjiang Wusu,China	1971.07.31
B31984001	B3	Antiqua		Xinjiang Wusu,China	1984
B31989002	B3	Antiqua		Xinjiang Wusu,China	1989.07.28
B31994002	B3	Antiqua		Xinjiang Wusu,China	1994.07.19
B31997001	B3	Antiqua		Xinjiang Jinghe,China	1997.06.29
B31999001	B3	Antiqua		Xinjiang Jinghe,China	1999.07.12
2547	B4	Antiqua		Xingjiang Nileke,China	2005.7.26
2548	B4	Antiqua		Xingjiang Nileke,China	2005.9.20
2652	O	Antiqua		Xinjiang Kelamayi,China	2006.10.10
42084	B3	Antiqua		Xinjiang Wusu,China	
B31968001	B3	Antiqua		Xinjiang Jinghe,China	1968.07.26
B31983001	B3	Antiqua		Xinjiang Wusu,China	1983.05.23
B31983002	B3	Antiqua	0.ANT1	Xinjiang Wusu,China	1983
B32001001	B3	Antiqua		Xinjiang Wusu,China	2001.07.07
K10000003	K1	Antiqua		Xinjiang Hetian,China	
K21987003	K2	Antiqua	0.ANT1	Xinjiang Qiemo,China	1987
42026	B3	Antiqua		Xinjiang Wusu,China	
45113	B3	Antiqua		Xinjiang Jinghe,China	
45115	B3	Antiqua		Xinjiang Jinghe,China	
45114	B3	Antiqua		Xinjiang Jinghe,China	
B21972002	B2	Antiqua	0.ANT1	Xinjiang Hutubi,China	1972
B30000002	B3	Antiqua		Xinjiang Wusu,China	
B30000004	B3	Antiqua		Xinjiang Wusu,China	
B30000010	B3	Antiqua		Xinjiang Wusu,China	
B30000011	B3	Antiqua		Xinjiang Wusu,China	
B30000012	B3	Antiqua		Xinjiang Wusu,China	
B31969001	B3	Antiqua		Xinjiang Jinghe,China	1969.08.01
B31981001	B3	Antiqua	0.ANT1	Xinjiang Jinghe,China	1981

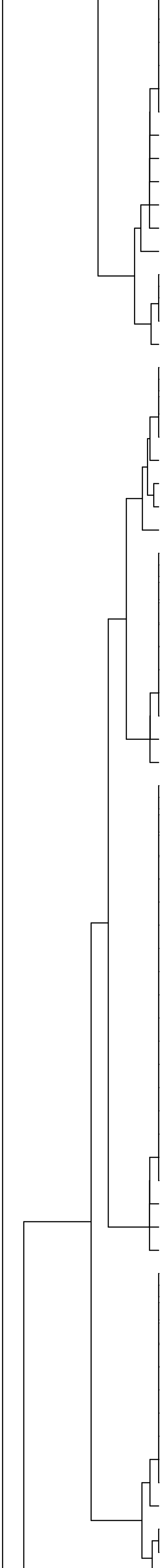

B31969001	B3	Antiqua		Xinjiang Jinghe,China	1969.08.01
B31981001	B3	Antiqua	0.ANT1	Xinjiang Jinghe,China	1981
B31982002	B3	Antiqua		Xinjiang Jinghe,China	1982.10.03
B32002014	B3	Antiqua		Xinjiang Wusu,China	2002.08.25
B32005017	B3	Antiqua		Xinjiang Wusu,China	2005
A1966001	A	Antiqua	0.ANT1	Xinjiang Wuqia,China	1966
B31965001	B3	Antiqua	0.ANT1	Xinjiang Jinghe,China	1965
B31967001	B3	Antiqua		Xinjiang Wusu,China	1967.08.01
B31984002	B3	Antiqua		Xinjiang Wusu,China	1984.08.21
B31987001	B3	Antiqua		Xinjiang Wusu,China	1987.07.21
B30000003	B3	Antiqua		Xinjiang Wusu,China	
B30000007	B3	Antiqua		Xinjiang Wusu,China	
B30000008	B3	Antiqua		Xinjiang Wusu,China	
B30000009	B3	Antiqua		Xinjiang Wusu,China	
B22001001	B2	Antiqua		Xinjiang Changji,China	2001.08.29
B21972003	B2	Antiqua		Xinjiang Manasi,China	1972.08.02
B21984001	B2	Antiqua		Xinjiang Changji,China	1984.08.08
B21997001	B2	Antiqua		Xinjiang Manasi,China	1997.07.26
B31967002	B3	Antiqua		Xinjiang Wusu,China	1967.08.08
B31972001	B3	Antiqua		Xinjiang Jinghe,China	1972.07.06
B41999002	B4	Antiqua		Xinjiang Yining,China	1999.07.12
K21985001	K2	Antiqua		Xinjiang Ruoqiang,China	1985.07.06
B2040040	B2	Antiqua		Xinjiang Manasi,China	
B21959002	B2	Antiqua		Xinjiang Hutubi,China	1959.09.12
B21960001	B2	Antiqua		Xinjiang Changji,China	1960.08.15
B21980003	B2	Antiqua		Xinjiang Hutubi,China	1980.08.28
B21983004	B2	Antiqua		Xinjiang Wulumuqi,China	1983.08.20
B21984002	B2	Antiqua		Xinjiang Changji,China	1984.08.08
B21989001	B2	Antiqua		Xinjiang Changji,China	1989.09.28
B21990001	B2	Antiqua		Xinjiang Wulumuqi,China	1990.05.23
B31999002	B3	Antiqua		Xinjiang Wusu,China	1999.08.06
43013	B2	Antiqua		Xingjiang Hutubi,China	
B22000002	B2	Antiqua		Xinjiang Hutubi,China	2000.07.29
44034	B2	Antiqua		Xinjiang Changji,China	
B2040060	B2	Antiqua		Xinjiang Manasi,China	
B2040061	B2	Antiqua		Xinjiang Manasi,China	
B21959001	B2	Antiqua	0.ANT1	Xinjiang Manasi,China	1959
B21961001	B2	Antiqua		Xinjiang Manasi,China	1961.08.19
B21972001	B2	Antiqua		Xinjiang Hutubi,China	1972.07.21
B21979001	B2	Antiqua		Xinjiang Changji,China	1979.08.10
B21980001	B2	Antiqua		Xinjiang Hutubi,China	1980.08.03
B21980002	B2	Antiqua		Xinjiang Hutubi,China	1980.08.07
B21983001	B2	Antiqua		Xinjiang Changji,China	1983
B21983002	B2	Antiqua		Xinjiang Wulumuqi,China	1983.07.16
B21983003	B2	Antiqua		Xinjiang Wulumuqi,China	1983.07.16
B21984003	B2	Antiqua	0.ANT1	Xinjiang Changji,China	1984
B21987001	B2	Antiqua		Xinjiang Manasi,China	1987.06.12
B21987002	B2	Antiqua		Xinjiang Manasi,China	1987.06.18
B21990002	B2	Antiqua		Xinjiang Wulumuqi,China	1990.05.30
CMCC8211	B3	Antiqua	0.ANT1	Xinjiang Jinghe,China	1982
K21985005	K2	Antiqua		Xinjiang Ruoqiang,China	1985
B41999001	B4	Antiqua		Xinjiang Yining,China	1999.07.12
B21965001	B2	Antiqua		Xinjiang Manasi,China	1965.08.01
B32001003	B3	Antiqua		Xinjiang Wusu,China	2001.08.01
B20003002	B2	Antiqua		Xinjiang Wulumuqi,China	2000
B20003003	B2	Antiqua		Xinjiang Wulumuqi,China	2000
B21999001	B2	Antiqua		Xinjiang Changji,China	1999.06.22
B22000003	B2	Antiqua		Xinjiang Changji,China	2000.09.23
B22002001	B2	Antiqua		Xinjiang Changji,China	2002.07.10
B22002003	B2	Antiqua		Xinjiang Changji,China	2002.07.22
B22002007	B2	Antiqua		Xinjiang Wulumuqi,China	2002.09.12
B22003003	B2	Antiqua		Xinjiang Wulumuqi,China	2003.09.08
B22003004	B2	Antiqua		Xinjiang Changji,China	2003.09.15
B22003005	B2	Antiqua		Xinjiang Wulumuqi,China	2003.09.18
B20003001	B2	Antiqua		Xinjiang Wulumuqi,China	2000
44036	B2	Antiqua		Xinjiang Changji,China	
B22002004	B2	Antiqua		Xinjiang Changji,China	2002.07.22

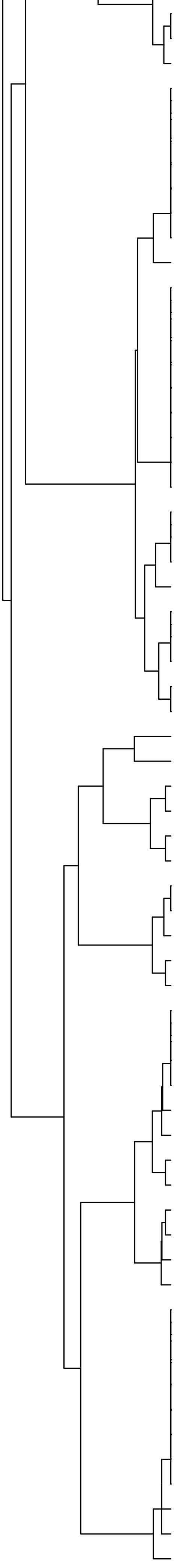

44036	B2	Antiqua		Xinjiang Changji,China	
B22002004	B2	Antiqua		Xinjiang Changji,China	2002.07.22
B22002002	B2	Antiqua		Xinjiang Changji,China	2002.07.22
B31978001	B3	Antiqua		Xinjiang Wusu,China	1978.09.08
B31989001	B3	Antiqua		Xinjiang Wusu,China	1989.07.24
B31989003	B3	Antiqua		Xinjiang Shawan,China	1989.09.18
B31994001	B3	Antiqua		Xinjiang Wusu,China	1994.06.30
B32002006	B3	Antiqua		Xinjiang Wusu,China	2002.09.05
B32002008	B3	Antiqua		Xinjiang Shawan,China	2002.08.23
B32003001	B3	Antiqua		Xinjiang Wusu,China	2003.06.09
B32002002	B3	Antiqua		Xinjiang Wusu,China	2002
2655	O	Antiqua		Xinjiang Kelamayi,China	2006.10.9
B21992001	B2	Antiqua		Xinjiang Hutubi,China	1992.08.26
B31988002	B3	Antiqua		Xinjiang Shawan,China	1988.08.10
B31988003	B3	Antiqua		Xinjiang Shawan,China	1988.08.12
B31989004	B3	Antiqua		Xinjiang Shawan,China	1993.09.01
945	B3	Antiqua	0.ANT1	Xinjiang Shawan,China	1994
B32002001	B3	Antiqua		Xinjiang Wusu,China	2002.07.02
B32002003	B3	Antiqua		Xinjiang Shawan,China	2002.08.08
B32002004	B3	Antiqua		Xinjiang Shawan,China	2002.08.08
B22002005	B2	Antiqua		Xinjiang Manasi,China	2002.09.05
B32001006	B3	Antiqua		Xinjiang Shawan,China	2001.08.17
B32002005	B3	Antiqua		Xinjiang Shawan,China	2002.08.08
B32001005	B3	Antiqua		Xinjiang Shawan,China	2001.08.17
B31988004	B3	Antiqua		Xinjiang Shawan,China	1988
B31989005	B3	Antiqua		Xinjiang Shawan,China	1989
B32002016	B3	Antiqua		Xinjiang Shawan,China	2002.08.08
B22002006	B2	Antiqua		Xinjiang Manasi,China	2002.09.05
B32001007	B3	Antiqua		Xinjiang Shawan,China	2001.08.17
757		Antiqua		Hentiy Aymag,Mongolia	1981
3056		Antiqua		Bayan-Uul,Mongolia	2003
B11996001	B1	Antiqua		Xinjiang Aheqi,China	1996.08.17
B11996002	B1	Antiqua		Xinjiang Aheqi,China	1996.08.17
B11965001	B1	Antiqua		Xinjiang Aheqi,China	1965.07.01
B11992001	B1	Antiqua		Xinjiang Aheqi,China	1992.07.18
B30000001	B4	Antiqua		Xinjiang Wenquan,China	
B42003002	B4	Antiqua		Xinjiang Wenquan,China	2003.07.24
B42003001	B4	Antiqua		Xinjiang Wenquan,China	2003.07.15
2330	B2	Antiqua	0.ANT2	Xinjiang Wenquan,China	2003
B42003004	B4	Antiqua	0.ANT2	China	2003
A1995001	A	Antiqua		Xinjiang Wuqia,China	1995.07.06
A1996001	A	Antiqua		Xinjiang Wuqia,China	1996.07.07
A1998001	A	Antiqua		Xinjiang Wuqia,China	1998.07.18
A1998002	A	Antiqua		Xinjiang Wuqia,China	1998.07.18
A1997001	A	Antiqua		Xinjiang Wuqia,China	1997.03.31
A1983001	A	Antiqua		Xinjiang Wuqia,China	1983.09.17
A1956001	A	Antiqua	0.ANT3	Xinjiang Wuqia,China	1956
A1960001	A	Antiqua		Xinjiang Wuqia,China	1960.08.28
B11979001	B1	Antiqua		Xinjiang Atushi,China	1979
B11992002	B1	Antiqua		Xinjiang Atushi,China	1992.08.26
B11995001	B1	Antiqua		Xinjiang Atushi,China	1995.08.08
B11995002	B1	Antiqua		Xinjiang Atushi,China	1995.08.08
A0041019	A	Antiqua		Xinjiang Wuqia,China	
A1996002	A	Antiqua		Xinjiang Wuqia,China	1996.08.04
A1996003	A	Antiqua		Xinjiang Wuqia,China	1996.08.08
B11989001	B1	Antiqua		Xinjiang Wuqia,China	1989.08.14
B11989002	B1	Antiqua		Xinjiang Atushi,China	1989.09.11
B11989003	B1	Antiqua		Xinjiang Wuqia,China	1989.09.11
B11997001	B1	Antiqua		Xinjiang Wuqia,China	1997.06.29
B12001001	B1	Antiqua		Xinjiang Wuqia,China	2001.07.18
A1992001	A	Antiqua	0.ANT3	Xinjiang Wuqia,China	1992
B12002001	B1	Antiqua		Xinjiang Wuqia,China	2002.07.16
41022	A	Antiqua		Xinjiang Wuqia,China	
