# Supplementary material for: Features of Variable Number of Tandem Repeats in Yersinia pestis and the Development of a Hierarchical Genotyping Scheme
Source: PLoS One. 2013 Jun 21;8(6):e66567. doi: 10.1371/journal.pone.0066567 (PMC3689786; doi:10.1371/journal.pone.0066567)
Supplement: Figure S8 — Dendrogram of Y. pestis strains clustered with 1.IN population based on 14+5 VNTR loci. A total of 207 strains were analyzed according to the profiles of 14 primary VNTRs and the loci M34, N3773, M33, M25, and M22. (PDF) [file pone.0066567.s008.pdf]

Categorical

207 1.IN strains with 19 loci

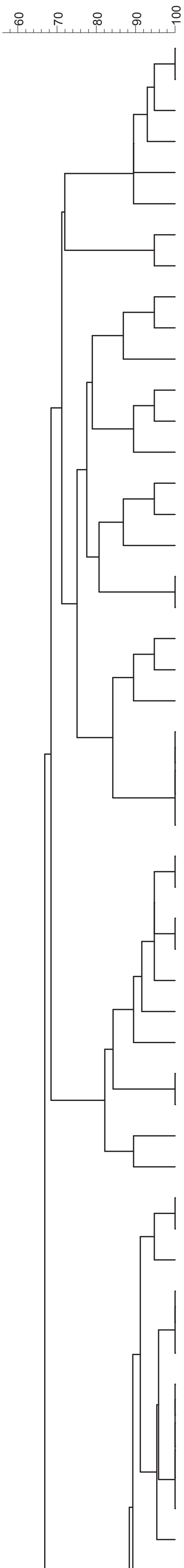

Strain ID	Focus	Biovar	Sub-pop	Location	Time
C1978003	C	Antiqua		Qinghai Yushu,China	1978.07.28
C1982002	C	Antiqua		Qinghai Yushu,China	1982.07.28
C1978010	C	Antiqua		Qinghai Nangqian,China	1978.10.27
C1985002	C	Antiqua		Qinghai Guinan,China	1985.07.29
C1978011	C	Antiqua		Qinghai Nangqian,China	1978.10.27
C1990004	C	Antiqua		Qinghai Nangqian,China	1990.08.16
C1964001	C	Antiqua		Qinghai Yushu,China	1964.07.11
C1964007	C	Antiqua		Qinghai Yushu,China	1964.10.17
C1985003	C	Antiqua		Qinghai Guinan,China	1985.07.31
D1985002	D	Antiqua		Qinghai Qilian,China	1985.10.07
C2003001	C	Antiqua		Qinghai Geermu,China	2003.06.27
C1974004	C	Antiqua		Qinghai Zhaduo,China	1974.09.06
C1974005	C	Antiqua		Qinghai Zhaduo,China	1974.09.23
C1969001	C	Antiqua		Qinghai Zhaduo,China	1969.07.23
C1983004	C	Antiqua		Tibe Chaya,China	1983.08.11
C1986003	C	Antiqua		Tibe Chaya,China	1986.08.21
C1980002	C	Antiqua	1.IN2	Qinghai Qumalai,China	1980
C1954001	C	Antiqua	1.IN2	Qinghai Guinan,China	1954
C1973002	C	Antiqua		Qinghai Nangqian,China	1973.09.09
C1962003	C	Antiqua	1.IN2	Qinghai Xinghai,China	1962
C1964005	C	Antiqua		Qinghai Gonghe,China	1964.09.12
C1990001	C	Antiqua		Qinghai Chengduo,China	1990.07.05
C1983001	C	Antiqua		Tibet Changdu,China	1983.07.03
C1983002	C	Antiqua		Tibet Changdu,China	1983.07.04
C1998001	C	Antiqua		Tibet Changdu,China	1998.05.29
C1998002	C	Antiqua		Tibet Dangxiong,China	1998.07.28
C1960003	C	Antiqua		Gansu Xiahe,China	1960.09.10
C1960004	C	Antiqua		Gansu Xiahe,China	1960.10.05
C0000001	C	Antiqua		Qinghai Zhunhua,China	
C1970009	C	Antiqua		Qinghai Zhunhua,China	1970.09.23
C1964004	C	Antiqua		Gansu Sunan,China	1964.08.20
C1960005	C	Antiqua		Gansu Xiahe,China	1960.11.05
C1960001	C	Antiqua	1.IN2	Qinghai Tongren,China	1960
C1986001	C	Antiqua		Qinghai Tongren,China	1986.06.24
C1986002	C	Antiqua		Qinghai Tongren,China	1986.07.26
C1959001	C	Antiqua		Gansu Xiahe,China	1959.08.01
H1954003	H	Antiqua	1.IN2	Inner Mongolia Baochang,China	1954
C1964006	C	Antiqua		Qinghai Haiyan,China	1964.10.10
C1971001	C	Antiqua		Qinghai Huangyuan,China	1971.05.14
C1970001	C	Antiqua		Qinghai Guide,China	1970.06.14
C1961002	C	Antiqua		Qinghai Gonghe,China	1961.06.02
C1968001	C	Antiqua		Qinghai Gangcha,China	1968.05.30
C1974002	C	Antiqua		Qinghai Tianjun,China	1974.08.01
C0000002	C	Antiqua		Qinghai Gonghe,China	
C1961003	C	Antiqua		Qinghai Gonghe,China	1961.06.15
C1966001	C	Antiqua		Qinghai Haiyan,China	1966.08.12
D1963002	D	Antiqua		Qinghai Menyuan,China	1963.07.02
D1963003	D	Antiqua		Qinghai Menyuan,China	1963.07.23
C1964003	C	Antiqua		Qinghai Haiyan,China	1964.08.15
C1964002	C	Antiqua		Qinghai Dulan,China	1964.08.11

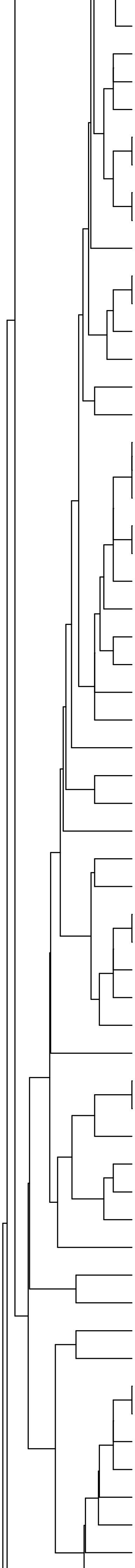

[illegible]	D	Antiqua		Qinghai [illegible],China	[illegible]
C1964003	C	Antiqua		Qinghai Haiyan,China	1964.08.15
C1964002	C	Antiqua		Qinghai Dulan,China	1964.08.11
C1975001	C	Antiqua		Qinghai Zhiduo,China	1975.08.01
C1970008	C	Antiqua		Qinghai Dulan,China	1970.09.15
D2001001	D	Antiqua		Qinghai Tongde,China	2001.05.27
D2001002	D	Antiqua		Qinghai Tongde,China	2001.06.04
C1996001	C	Antiqua		Qinghai Delingha,China	1996.07.24
D2001003	D	Antiqua		Qinghai Tongde,China	2001.06.04
C1973001	C	Antiqua		Qinghai Gangcha,China	1973.08.17
C1979002	C	Antiqua	1.IN2	Qinghai Mangya,China	1979
D1963001	D	Antiqua		Qinghai Qilian,China	1963.06.02
C1961007	C	Antiqua		Qinghai Xinghai,China	1961.6.11
C2002001	C	Antiqua		Qinghai Delingha,China	2002.08.22
C1970002	C	Antiqua		Qinghai Xinghai,China	1970.06.17
F1952001	F	Antiqua	1.IN2	Yunnan Midu,China	1952
C1967002	C	Antiqua		Qinghai Gonghe,China	1967.06.19
C1970006	C	Antiqua		Qinghai Gonghe,China	1970.07.29
C1970007	C	Antiqua		Qinghai Guide,China	1970.08.09
C1966002	C	Antiqua		Qinghai Tianjun,China	1966.09.01
C1974001	C	Antiqua		Qinghai Gangcha,China	1974.07.13
C1974003	C	Antiqua		Qinghai Gangcha,China	1974.08.24
C1967003	C	Antiqua		Qinghai Gonghe,China	1967.09.20
C1970005	C	Antiqua	1.IN2	Qinghai Gonghe,China	1970
C1985001	C	Antiqua		Qinghai Gonghe,China	1985.07.06
C1971003	C	Antiqua		Qinghai Huangyuan,China	1971.06.19
D0000001	D	Antiqua		Qinghai Qilian,China	
C2000002	C	Antiqua		Qinghai Tianjun,China	2000.09.11
C1983006	C	Antiqua		Qinghai Haiyan,China	1983.08.26
C2000001	C	Antiqua	1.IN2	Qinghai Delingha,China	2000
C1960002	C	Antiqua		Qinghai Haiyan,China	1960.06.25
C1961004	C	Antiqua		Gansu Sunan,China	1961.06.28
C1972004	C	Antiqua		Gansu Yumen,China	1972.07.26
C1971004	C	Antiqua		Gansu Subei,China	1971.08.05
C1983007	C	Antiqua		Gansu Subei,China	1983.09.13
C1982001	C	Antiqua		Gansu Subei,China	1982.07.17
D1975001	D	Antiqua		Gansu Sunan,China	1975.06.
C1977001	C	Antiqua		Gansu Yumen,China	1977.09.30
C1969002	C	Antiqua		Qinghai Wulan,China	1969.08.25
C1988001	C	Antiqua		Qinghai Xinghai,China	1988.03.08
D0000002	D	Antiqua		Qinghai Qilian,China	
C1988002	C	Antiqua		Qinghai Xinghai,China	1988.03.10
C2004005	C	Antiqua		Qinghai Wulan,China	2004
D1990001	D	Antiqua		Qinghai Qilian,China	1990.06.08
D0000003	D	Antiqua		Qinghai Qilian,China	
C1993002	C	Antiqua		Gansu Yumen,China	1993
C1967001	C	Antiqua		Qinghai Wulan,China	1967
C2003002	C	Antiqua		Qinghai Wulan,China	2003.08.16
C1978009	C	Antiqua		Tibet Dingqing,China	1978.09.07
C1990002	C	Antiqua		Tibe Baqing,China	1990.08.04
C1985004	C	Antiqua		Qinghai Guinan,China	1985.08.19
C1997001	C	Antiqua		Qinghai Nangqian,China	1997.11.15
D1991001	D	Antiqua		Qinghai Tongde,China	1991.09.05
D1991004	D	Antiqua	1.IN2	Qinghai Zeku,China	1991
D1991003	D	Antiqua		Qinghai Tongde,China	1991.09.05
D1991005	D	Antiqua	1.IN2	Qinghai Zeku,China	1991
D1991006	D	Antiqua		Qinghai Zeku,China	1991.09.10

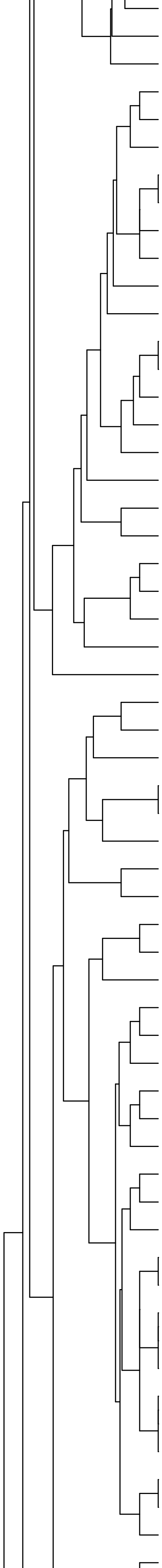

D1991005	D	Antiqua	1.IN2	Qinghai Zeku,China	1991
D1991006	D	Antiqua		Qinghai Zeku,China	1991.09.10
D1991002	D	Antiqua		Qinghai Zeku,China	1991.09.05
D1964002	D	Antiqua	1.IN2	Qinghai Menyuan,China	1964
D1967001	D	Antiqua		Qinghai Menyuan,China	1967.08.23
D1983001	D	Antiqua		Qinghai Menyuan,China	1983.04.04
D1965001	D	Antiqua		Qinghai Qilian,China	1965.09.21
D1977001	D	Antiqua		Qinghai Qilian,China	1977.09.21
D0000004	D	Antiqua		Qinghai Menyuan,China	
D1958001	D	Antiqua		Qinghai Qilian,China	1958
D1960001	D	Antiqua		Qinghai Qilian,China	1960.05.26
D1964001	D	Antiqua	1.IN2	Qinghai Menyuan,China	1964
C1961005	C	Antiqua		Gansu Sunan,China	1961.07.16
D1985001	D	Antiqua		Gansu Sunan,China	1985.06.08
C1954002	C	Antiqua		Qinghai Heinan,China	1954
C1954003	C	Antiqua		Qinghai Heinan,China	1954
C1983003	C	Antiqua		Gansu Akesai,China	1983.07.11
D1982001	D	Antiqua	1.IN2	Gansu Sunan,China	1982
C1979003	C	Antiqua		Qinghai Maqin,China	1979.08.03
C1980001	C	Antiqua		Qinghai Maqin,China	1980.10.18
C1980004	C	Antiqua		Qinghai Maduo,China	1980.11.07
C1994003	C	Antiqua		Qinghai Dulan,China	1994.09.24
C1983008	C	Antiqua		Qinghai Maduo,China	1983.11.06
C1978001	C	Antiqua		Qinghai Maduo,China	1978.07.24
D1984001	D	Antiqua	1.IN2	Gansu Sunan,China	1984
C1983005	C	Antiqua		Qinghai Zhiduo,China	1983.08.14
C1993001	C	Antiqua		Qinghai Chengduo,China	1993.08.05
C1971002	C	Antiqua		Qinghai Guide,China	1971.06.04
C1978007	C	Antiqua		Qinghai Zhiduo,China	1978.08.25
C1978008	C	Antiqua		Qinghai Zhiduo,China	1978.08.25
C1965001	C	Antiqua		Qinghai Yushu,China	1965.06.03
C1989001	C	Antiqua	1.IN2	Qinghai Zhaduo,China	1989
C1998003	C	Antiqua		Qinghai Qumalai,China	1998.09.15
C1970003	C	Antiqua		Qinghai Qumalai,China	1970.06.30
C1970004	C	Antiqua		Qinghai Qumalai,China	1970.06.30
C1980003	C	Antiqua		Tibet Biru,China	1980.11.03
C1976002	C	Antiqua	1.IN2	Tibet Naqu,China	1976
C1978002	C	Antiqua		Tibet Naqu,China	1978.07.27
C1992001	C	Antiqua		Tibe Nierong,China	1992.07.14
C1987001	C	Antiqua		Tibe Aanduo,China	1987.07.21
C1988003	C	Antiqua		Tibe Aanduo,China	1988.06.10
C1990003	C	Antiqua		Tibe Aanduo,China	1990.08.04
C1976007	C	Antiqua		Tibet Naqu,China	1976.08.07
C1994004	C	Antiqua		Tibet Dangxiong,China	1994.10.23
C1994002	C	Antiqua	1.IN2	Tibet Biru,China	1994
C1987002	C	Antiqua		Tibe Aanduo,China	1987.08.11
C1992002	C	Antiqua		Tibe Aanduo,China	1992.08.05
C1978004	C	Antiqua		Tibet Naqu,China	1978.08.04
C1978005	C	Antiqua		Tibet Naqu,China	1978.08.04
C1978006	C	Antiqua		Tibet Naqu,China	1978.08.18
C1962001	C	Antiqua		Qinghai Huangyuan,China	1962.08.13
C1976003	C	Antiqua		Tibet Naqu,China	1976.06.24
C1976004	C	Antiqua		Tibet Naqu,China	1976.07.12
C1976005	C	Antiqua		Tibet Naqu,China	1976.07.14
C1976006	C	Antiqua		Tibet Naqu,China	1976.07.31
C1988004	C	Antiqua		Tibe Nierong,China	1988.09.13
180001	C	Antiqua		Qinghai Luhuo,China	1990.5.28

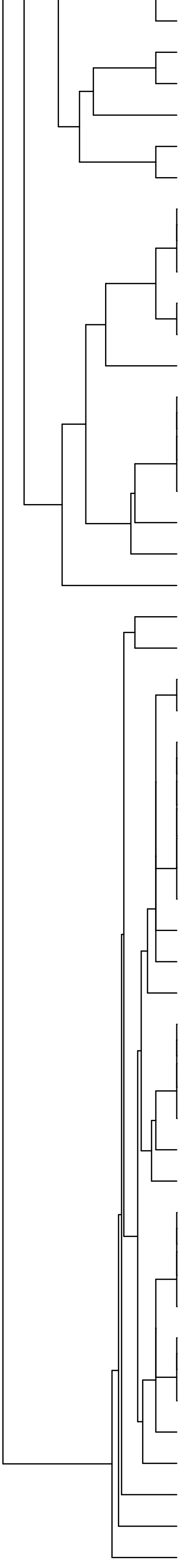

C1988004	C	Antiqua		Tibe Nierong,China	1988.09.13
180001	C	Antiqua		Qinghai Luhuo,China	1990.5.28
C1991001	C	Antiqua		Qinghai Geermu,China	1991.08.24
C1992003	C	Antiqua		Qinghai Zhiduo,China	1992.11.03
C1975002	C	Antiqua		Qinghai Yushu,China	1975.09.01
C1975003	C	Antiqua	1.IN2	Qinghai Yushu,China	1975
2650	O	Antiqua		Xinjiang Kelamayi,China	2006.10.9
2651	O	Antiqua		Xingjiang Mosuowan,China	2006.10.9
2656	O	Antiqua		Xinjiang Qitai,China	2006.10.06
2638	K2	Antiqua		Xinjiang Qiemo,China	2006.08.09
2640	K2	Antiqua		Xinjiang Qiemo,China	2006.08.09
C1979001	C	Antiqua		Qinghai Mangya,China	1979.07.19
K21985002	K2	Antiqua	1.IN1	Xinjiang Ruoqiang,China	1985
K21985004	K2	Antiqua		Xinjiang Ruoqiang,China	1985.07.25
K21985006	K2	Antiqua	1.IN1	Xinjiang Ruoqiang,China	1985
K21985007	K2	Antiqua		Xinjiang Ruoqiang,China	1985.08.11
K21985003	K2	Antiqua		Xinjiang Ruoqiang,China	1985.07.21
K22005001	K2	Antiqua		Xinjiang Ruoqiang,China	2005.10.9
K21985008	K2	Antiqua		Xinjiang Ruoqiang,China	1985.08.27
E1957004	E	Antiqua		Yunnan Jianchuan,China	1957
E1982001	E	Antiqua		Yunnan Jianchuan,China	1982.12.23
E1957003	E	Antiqua		Yunnan Jianchuan,China	1957
E1985001	E	Antiqua		Yunnan Jianchuan,China	1985
E1957002	E	Antiqua		Yunnan Jianchuan,China	1957
E1977001	E	Antiqua	1.IN3	Yunnan Jianchuan,China	1977
E1977004	E	Antiqua		Yunnan Jianchuan,China	1977.08.03
E1980001	E	Antiqua		Yunnan Jianchuan,China	1980
E1984004	E	Antiqua		Yunnan Jianchuan,China	1984
F2000001	F	Antiqua		Yunnan Yingjiang,China	2000
E1977002	E	Antiqua		Yunnan Jianchuan,China	1977.07.31
F1984005	F	Antiqua		Yunnan Longchuan,China	1984
E1979001	E	Antiqua	1.IN3	China	1979
E1954001	E	Antiqua	1.IN3	Yunnan Jianchuan,China	1954
E1955001	E	Antiqua		Yunnan Jianchuan,China	1955.06.12
E1979004	E	Antiqua		Yunnan Jianchuan,China	1979
F1954003	F	Antiqua		Yunnan Xiangyun,China	1954.12.09
F1954001	F	Antiqua		Yunnan Xiangyun,China	1954.11.08
E1983001	E	Antiqua		Yunnan Jianchuan,China	1983
E1979003	E	Antiqua		Yunnan Jianchuan,China	1979.12.31
E1982002	E	Antiqua		Yunnan Jianchuan,China	1982.12.28
E1983004	E	Antiqua		Yunnan Jianchuan,China	1983.10.03
E1984002	E	Antiqua		Yunnan Jianchuan,China	1984.04.27
E1957001	E	Antiqua		Yunnan Jianchuan,China	1957
E1990001	E	Antiqua		Yunnan Jianchuan,China	1990
F1990004	F	Antiqua		Yunnan Yingjiang,China	1990
E1984003	E	Antiqua		Yunnan Jianchuan,China	1984.04.27
E1984006	E	Antiqua		Yunnan Jianchuan,China	1984.08.13
E1954003	E	Orientalis		Yunnan Jianchuan,China	1954
E1977003	E	Antiqua		Yunnan Jianchuan,China	1977
F1954005	F	Orientalis		Yunnan Xiangyun,China	1954
