# Supplementary material for: Features of Variable Number of Tandem Repeats in Yersinia pestis and the Development of a Hierarchical Genotyping Scheme
Source: PLoS One. 2013 Jun 21;8(6):e66567. doi: 10.1371/journal.pone.0066567 (PMC3689786; doi:10.1371/journal.pone.0066567)
Supplement: Figure S9 — Dendrogram of Y. pestis strains clustered with 1.ORI population based on 14+4 VNTR loci. A total of 196 strains were analyzed according to the profiles of 14 primary VNTRs and the loci M29, M28, N1606, and N2117. (PDF) [file pone.0066567.s009.pdf]

Categorical

196 1.ORI strains with 18 loci

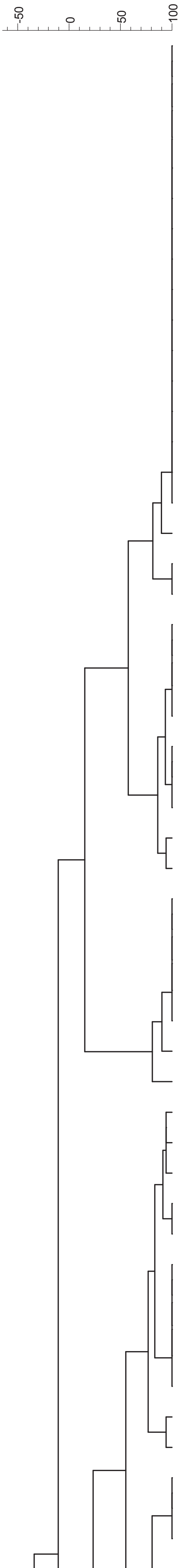

Strain ID	Focus	Biovar	Sub-pop	Location	Time
F1990021	F	Orientalis		Yunnan Gengma,China	1990
F1990025	F	Orientalis		Yunnan Gengma,China	1990
F1991001	F	Orientalis		Yunnan Luoxi,China	1991
F1991007	F	Orientalis		Myanmar	1991
F1991008	F	Orientalis		Yunnan Zhenkang,China	1991
F1991009	F	Orientalis		Myanmar	1991
F1991012	F	Orientalis		Yunnan Gengma,China	1991
F1991018	F	Orientalis		Yunnan Gengma,China	1991
F1992003	F	Orientalis		Yunnan Linchang,China	1992
F1992004	F	Orientalis		Yunnan Lanchang,China	1992.02.20
F1992005	F	Orientalis		Yunnan Menghai,China	1992
F1993001	F	Orientalis		Yunnan Lanchang,China	1993.01.31
F1996004	F	Orientalis		Yunnan Gengma,China	1996.05.11
F1996005	F	Orientalis		Yunnan Gengma,China	1996.05.13
F2001006	F	Orientalis		Yunnan Linchang,China	2001
F2002007	F	Orientalis		Guizhou Yixing,China	2002.10.12
F1992002	F	Orientalis		Yunnan Linchang,China	1992
F2000004	F	Orientalis	1.ORI2	Guizhou Yixing,China	2000
F2001008	F	Orientalis		Yunnan Fengqing,China	2001
F1990022	F	Orientalis		Yunnan Gengma,China	1990
F1990023	F	Orientalis		Yunnan Gengma,China	1990
F1991006	F	Orientalis		Yunnan Zhenkang,China	1991.07.08
F1995005	F	Orientalis		Yunnan Zhenkang,China	1995
F1987001	F	Orientalis		Yunnan Wanting,China	1987
F1991011	F	Orientalis		Yunnan Gengma,China	1991.08.18
F1996006	F	Orientalis		Yunnan Gengma,China	1996
F1992012	F	Orientalis		Yunnan Changyuan,China	1992.11.12
F1996001	F	Orientalis		Yunnan Mojiang,China	1996.04.03
F1996025	F	Orientalis		Yunnan Shiping,China	1996
F1999001	F	Orientalis		Yunnan Jinghong,China	1999
F1999007	F	Orientalis		Yunnan Zhenyuan,China	1999
F1999011	F	Orientalis		Yunnan Zhenyuan,China	1999
F2001001	F	Orientalis		Yunnan Simao,China	2001
F1999010	F	Orientalis		Yunnan Zhenyuan,China	1999
F0000003	F	Orientalis		Yunnan Lanchang,China	
F1983012	F	Orientalis		Yunnan Ruili,China	1983
F1999004	F	Orientalis		Yunnan Luoxi,China	1999
F1992010	F	Orientalis		Yunnan Shuangjiang,China	1992
F1991017	F	Orientalis		Yunnan Gengma,China	1991
F1997001	F	Orientalis		Yunnan Kaiyuan,China	1997
F1990001	F	Orientalis		Yunnan Ruili,China	1990
F1995009	F	Orientalis		Yunnan Yingjiang,China	1995
F1991015	F	Orientalis		Yunnan Changyuan,China	1991
F1994003	F	Orientalis		Yunnan Tengchong,China	1994
F1994007	F	Orientalis		Yunnan Shidian,China	1994
F1990010	F	Orientalis		Yunnan Longchuan,China	1990.08.04
F1992001	F	Orientalis		Yunnan Lanchang,China	1992.01.22
F1990011	F	Orientalis		Yunnan Lianghe,China	1990
F1996014	F	Orientalis		Yunnan Lianghe,China	1996
F1994006	F	Orientalis		Yunnan Shidian,China	1994
F1982002	F	Orientalis		Yunnan Longchuan,China	1982.07.28

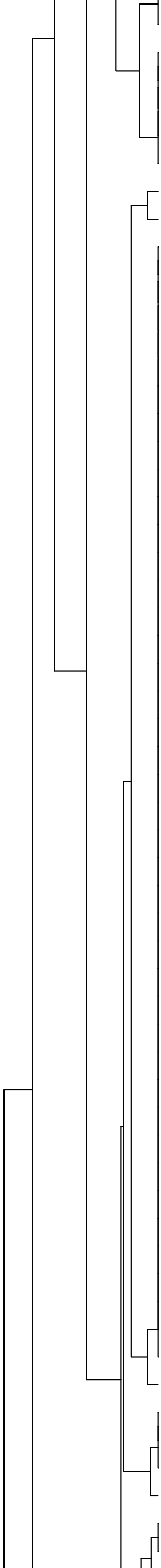

[illegible]	F	[illegible]		[illegible]	[illegible]
F1994006	F	Orientalis		Yunnan Shidian,China	1994
F1982002	F	Orientalis		Yunnan Longchuan,China	1982.07.28
F1982003	F	Orientalis		Yunnan Ruili,China	1982.08.13
F1983006	F	Orientalis		Yunnan Baoshan,China	1983.09.06
F1990013	F	Orientalis		Yunnan Yingjiang,China	1990.09.18
F1990015	F	Orientalis		Yunnan Lianghe,China	1990.11.03
YN663	F	Orientalis	1.ORI2	Yunnan Longchuan,China	1982
F1991016	F	Orientalis	1.ORI2	China	1991
F1982001	F	Orientalis		Yunnan Longchuan,China	1982.07.28
F1982004	F	Orientalis		Yunnan Ruili,China	1982.08.15
F1982005	F	Orientalis		Yunnan Ruili,China	1982.08.30
F1982006	F	Orientalis		Yunnan Longchuan,China	1982
F1982007	F	Orientalis		Yunnan Ruili,China	1982
F1983001	F	Orientalis		Yunnan Ruili,China	1983
F1983002	F	Orientalis		Yunnan Ruili,China	1983.08.11
F1983003	F	Orientalis		Yunnan Baoshan,China	1983.08.24
F1983004	F	Orientalis		Yunnan Ruili,China	1983
F1983005	F	Orientalis		Yunnan Baoshan,China	1983.09.06
F1983007	F	Orientalis		Yunnan Baoshan,China	1983.09.07
F1983008	F	Orientalis		Yunnan Longyang,China	1983
F1983009	F	Orientalis		Yunnan Longyang,China	1983
F1983010	F	Orientalis		Yunnan Longyang,China	1983
F1983011	F	Orientalis		Yunnan Ruili,China	1983
F1984001	F	Orientalis	1.ORI2	Yunnan Yingjiang,China	1984
F1984004	F	Orientalis		Yunnan Yingjiang,China	1984
F1986003	F	Orientalis		Yunnan Yingjiang,China	1986
F1990002	F	Orientalis		Yunnan Ruili,China	1990
F1990005	F	Orientalis		Yunnan Yingjiang,China	1990
F1990006	F	Orientalis		Yunnan Yingjiang,China	1990
F1990007	F	Orientalis		Yunnan Yingjiang,China	1990
F1990008	F	Orientalis		Yunnan Dali,China	1990
F1990009	F	Orientalis		Yunnan Dali,China	1990
F1990012	F	Orientalis		Yunnan Lianghe,China	1990
F1990014	F	Orientalis		Yunnan Yingjiang,China	1990.10.05
F1990016	F	Orientalis		Yunnan Longchuan,China	1990.11.08
F1990017	F	Orientalis		Yunnan Lianghe,China	1990.11.15
F1990018	F	Orientalis		Yunnan Lianghe,China	1990.11.19
F1990019	F	Orientalis		Yunnan Lianghe,China	1990.11.19
F1991010	F	Orientalis		Yunnan Yingjiang,China	1991
F1991013	F	Orientalis		Yunnan Longchuan,China	1991
F1991014	F	Orientalis		Yunnan Changyuan,China	1991
F1992008	F	Orientalis		Yunnan Yuanjiang,China	1992
F1992009	F	Orientalis		Yunnan Shuangjiang,China	1992
F1992014	F	Orientalis		Yunnan Changyuan,China	1992.11.26
F1994002	F	Orientalis		Yunnan Tengchong,China	1994
F1994004	F	Orientalis		Yunnan Shidian,China	1994
F1994005	F	Orientalis		Yunnan Shidian,China	1994
F1996015	F	Orientalis		Yunnan Lianghe,China	1996
F1997002	F	Orientalis		Yunnan Yanshan,China	1997
F1992013	F	Orientalis		Yunnan Changyuan,China	1992.11.16
F1992006	F	Orientalis		Yunnan Menghai,China	1992
F1999005	F	Orientalis		Yunnan Lianghe,China	1999
F1999012	F	Orientalis		Yunnan Luoxi,China	1999
F1992007	F	Orientalis		Yunnan Yuanjiang,China	1992
F1992011	F	Orientalis		Yunnan Shuangjiang,China	1992
F1994001	F	Orientalis		Yunnan Tengchong,China	1994

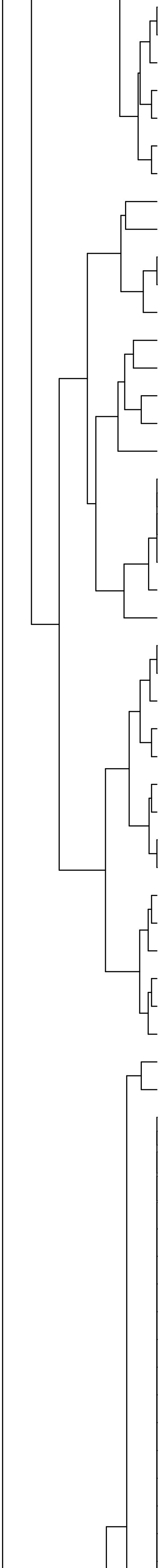

F1992011	F	Orientalis		Yunnan Shuangjiang,China	1992
F1994001	F	Orientalis		Yunnan Tengchong,China	1994
F1985001	F	Orientalis		Yunnan Ruili,China	1985
F1986001	F	Orientalis		Yunnan Yingjiang,China	1986
F1986002	F	Orientalis		Yunnan Yingjiang,China	1986
F1990003	F	Orientalis		Yunnan Ruili,China	1990
F1990024	F	Orientalis		Yunnan Gengma,China	1990
F1946001	F	Orientalis	1.ORI2	Fujian Gutian,China	1946
IP275		Orientalis	1.ORI3	Madagascar	1995
F1943001	F	Orientalis		Fujian Fuzhou,China	1943
F1952004	F	Orientalis		Fujian Putian,China	1952
F1950001	F	Orientalis		Fujian Huaan,China	1950
CO92		Orientalis	1.ORI1	USA	1992
FV-I		Orientalis	1.ORI1	USA	2001
CA88-4125		Orientalis	1.ORI1	USA	1988
India195		Orientalis	1.ORI1	India	>1898
MG05-1020		Orientalis	1.ORI3	Madagascar	2005
E1983002	E	Orientalis		Yunnan Jianchuan,China	1983
F1954002	F	Orientalis		Yunnan Xiangyun,China	1954
F1958001	F	Orientalis		Yunnan Dali,China	1958
F1964001	F	Orientalis		Yunnan Lianghe,China	1964
E1983003	E	Orientalis		Yunnan Jianchuan,China	1983
F1943002	F	Orientalis		Fujian Fuzhou,China	1943
F1995007	F	Orientalis		Myanmar	1995
F2001005	F	Orientalis		Yunnan Yanshan,China	2001
F1995008	F	Orientalis		Myanmar	1995
F1996012	F	Orientalis		Yunnan Menglian,China	1996
F1996013	F	Orientalis		Yunnan Menglian,China	1996
F1991002	F	Orientalis		Yunnan Wanting,China	1991.06.07
F1991005	F	Orientalis		Yunnan Wanting,China	1991
F1991003	F	Orientalis		Yunnan Wanting,China	1991.06.10
F1991004	F	Orientalis		Yunnan Wanting,China	1991.06.12
F1952002	F	Orientalis		Yunnan Midu,China	1952.11.27
F1952003	F	Orientalis		Yunnan Midu,China	1952
F1953003	F	Orientalis	1.ORI2	Yunnan Midu,China	1953
F1953002	F	Orientalis		Yunnan Baoshan,China	1953
F1955001	F	Orientalis		Yunnan Xiangyun,China	1955.02.18
F1954004	F	Orientalis		Yunnan Xiangyun,China	1954
F1995003	F	Orientalis		Yunnan Jiangcheng,China	1995
F2001007	F	Orientalis		Yunnan Yunxian,China	2001
F0000001	F	Orientalis		Yunnan Yanshan,China	
F0000002	F	Orientalis		Guangxi Longlin,China	
F1995001	F	Orientalis		Yunnan Puer,China	1995
F1995004	F	Orientalis		Yunnan Lanchang,China	1995
F1996008	F	Orientalis		Yunnan Mojiang,China	1996
F1996010	F	Orientalis		Yunnan Mojiang,China	1996
F1996011	F	Orientalis		Yunnan Mojiang,China	1996
F1996016	F	Orientalis		Yunnan Jianshui,China	1996
F1996017	F	Orientalis		Yunnan Jianshui,China	1996
F1996020	F	Orientalis		Yunnan Gejiu,China	1996
F1996022	F	Orientalis		Yunnan Wenshan,China	1996
F1996023	F	Orientalis		Yunnan Yanshan,China	1996
F1996024	F	Orientalis		Yunnan Shiping,China	1996
F1996026	F	Orientalis		Yunnan Shiping,China	1996
F1996027	F	Orientalis		Yunnan Shiping,China	1996
F1997003	F	Orientalis		Yunnan Yiliang,China	1997
F1997004	F	Orientalis		Yunnan Fumin,China	1997

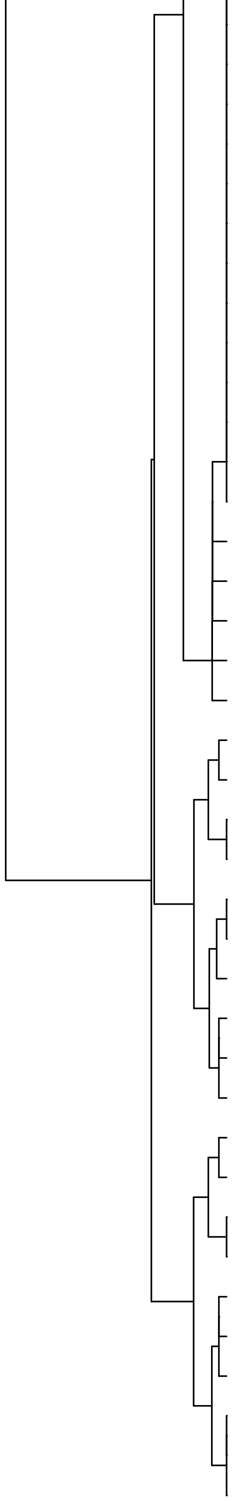

F1997003	F	Orientalis	Yunnan Yiliang,China	1997
F1997004	F	Orientalis	Yunnan Fumin,China	1997
F1997005	F	Orientalis	Yunnan Fumin,China	1997
F2000002	F	Orientalis	Guizhou Yixing,China	2000.08.01
F2000003	F	Orientalis	Guizhou Yixing,China	2000.08.07
F2000006	F	Orientalis	Guangxi Longlin,China	2000
F2000007	F	Orientalis	Guangxi Longlin,China	2000
F2000008	F	Orientalis	Guangxi Longlin,China	2000
F2000009	F	Orientalis	Guangxi Longlin,China	2000
F2000010	F	Orientalis	Guangxi Longlin,China	2000
F2000011	F	Orientalis	Guangxi Longlin,China	2000
F2002004	F	Orientalis	Yunnan Mile,China	2002
F2002005	F	Orientalis	Yunnan Mile,China	2002
F2002002	F	Orientalis	Yunnan Wenshan,China	2002
F1996002	F	Orientalis	Yunnan Mojiang,China	1996.04.03
F1999008	F	Orientalis	Yunnan Yunxian,China	1999
F2001003	F	Orientalis	Yunnan Pingbian,China	2001
F2001004	F	Orientalis	Yunnan Mengzi,China	2001
F1996003	F	Orientalis	Yunnan Yunxian,China	1996
F2001002	F	Orientalis	Yunnan Pingbian,China	2001
F1993002	F	Orientalis	Yunnan Jinghong,China	1993
F1993003	F	Orientalis	Yunnan Jinghong,China	1993
F2000005	F	Orientalis	Guizhou Yixing,China	2000.08.20
F2002006	F	Orientalis	Guizhou Yixing,China	2002.10.08
F2002003	F	Orientalis	Yunnan Yanshan,China	2002
F1999002	F	Orientalis	Yunnan Mengla,China	1999
F1999003	F	Orientalis	Yunnan Mengla,China	1999
F2002001	F	Orientalis	Yunnan Honghe,China	2002
F1996021	F	Orientalis	Yunnan Gejiu,China	1996
F1999006	F	Orientalis	Yunnan Yunxian,China	1999
F0000004	F	Orientalis	Yunnan Mengzi,China	
F1996009	F	Orientalis	Yunnan Mojiang,China	1996
F1995006	F	Orientalis	Yunnan Jinggu,China	1995
F1999009	F	Orientalis	Yunnan Honghe,China	1999
F1996018	F	Orientalis	Yunnan Jianshui,China	1996
F1995002	F	Orientalis	Yunnan Puer,China	1995
F1996007	F	Orientalis	Yunnan Mojiang,China	1996.08.26
F1996019	F	Orientalis	Yunnan Gejiu,China	1996
